# Non-POU Domain-Containing Octomer-Binding (NONO) protein expression and stability promotes the tumorigenicity and activation of Akt/MAPK/β-catenin pathways in human breast cancer cells

**DOI:** 10.1186/s12964-023-01179-0

**Published:** 2023-06-27

**Authors:** Bilal Ahmad Lone, Fouzia Siraj, Ira Sharma, Shweta Verma, Shibendra Kumar Lal Karna, Faiz Ahmad, Preeti Nagar, Chetana Sachidanandan, Yuba Raj Pokharel

**Affiliations:** 1 Cancer Biology Laboratory, Faculty of Life Science and Biotechnology, South Asian University, Rajpur Road, Maidangarhi, New Delhi, 110068 India; 2 National Institute of Pathology, Safdarjung Hospital Campus, Room No.610, 6th Floor, Ansari Nagar, New Delhi, 110029 India; 3 CSIR-Institute of Genomics and Integrative Biology (CSIR-IGIB), New Delhi, 110025 India; 4 Academy of Scientific and Innovative Research (AcSIR), Gaziabad, 201002 India

**Keywords:** Breast cancer, NONO, PIN1, MAPK/β-catenin, P53, Metastasis, Apoptosis, Cancer stem cells

## Abstract

Breast cancer is one of the most common cancers with a high mortality rate, underscoring the need to identify new therapeutic targets. Here we report that non-POU domain-containing octamer-binding (NONO) protein is overexpressed in breast cancer and validated the interaction of the WW domain of PIN1 with c-terminal threonine-proline (thr-pro) motifs of NONO. The interaction of NONO with PIN1 increases the stability of NONO by inhibiting its proteasomal degradation, and this identifies PIN1 as a positive regulator of NONO in promoting breast tumor development. Functionally, silencing of NONO inhibits the growth, survival, migration, invasion, epithelial to mesenchymal transition (EMT), and stemness of breast cancer cells in vitro. A human metastatic breast cancer cell xenograft was established in transparent zebrafish (*Danio rerio*) embryos to study the metastatic inability of NONO-silenced breast cancer cells in vivo. Mechanistically, NONO depletion promotes the expression of the PDL1 cell-surface protein in breast cancer cells. The identification of novel interactions of NONO with c-Jun and β-catenin proteins and activation of the Akt/MAPK/β-catenin signaling suggests that NONO is a novel regulator of Akt/MAPK/β-catenin signaling pathways. Taken together, our results indicated an essential role of NONO in the tumorigenicity of breast cancer and could be a potential target for anti-cancerous drugs.

Video Abstract

**Supplementary Information:**

The online version contains supplementary material available at 10.1186/s12964-023-01179-0.

## Background

With an estimated 2.3 million new cases, breast cancer is the most frequently diagnosed type of cancer worldwide [1]. It has a high recurrence rate and a tendency to metastasize, which is consistent with the pathological conditions [2]. Once metastasized, the cancer cells develop stemness and become more resistant to apoptosis, making treatment more challenging [3, 4]. Therapeutic strategies for breast cancer have improved significantly; however, patients with distant metastatic breast cancer are less responsive to standard therapies. Although targets like Her2, ER, or PR are promising molecules in the treatment of certain breast cancers, there is still a need to explore the role of other proteins and to analyze their molecular mechanism in breast cancer development in order to understand the disease and develop effective targeted therapies for breast cancer patients.

NONO, also known as 54 kD nuclear RNA and DNA binding protein (p54nrb), is a multifunctional DBHS (Drosophila behavior/human splicing) protein defined by N-terminal RNA recognition motifs (RRMs), protein-protein-interaction NONA/paraspeckle domain (NOPS) and a C-terminal coiled-coil domain [5, 6] binds DNA, RNA and proteins [5]. The NONO protein is predominantly localized in the cell’s nucleus, especially in paraspeckles [7]. It is involved in every step of gene regulation: transcriptional activation and repression [8], transcription termination [8], pre-mRNA splicing [8], RNA transport [9] as well as nuclear retention of defective RNA for editing [10]. The interaction of NONO with splicing factor proline and glutamine-rich (SFPQ) is critical for the recruitment of complex to the sites of DNA damage and subsequent activation of DNA repair pathways [11]. Since NONO plays an important role in several processes, it is dysregulated in many types of cancer. NONO has been shown to play an important role in the regulation of lipid metabolism in breast cancer cells. Specifically, NONO interacts with and stabilizes sterol regulatory element-binding protein 1 (SREBP1), a transcription factor that functions as a master regulator of lipid metabolism [12]. Modulation of EGFR and STAT3 stabilization by NONO exerts oncogenic behavior, chemotherapy resistance and poor prognosis in patients with triple-negative breast cancer (TNBC) [13, 14]. In addition, NONO promotes breast cancer cell growth by regulating the post-transcriptional expression of S-phase associated kinase 2 (SKP2) and E2F transcription factor [15]. In liver cancer, NONO contributes to carcinogenesis through oncogenic splicing of the BIN1 pre-mRNA [16]. NONO is highly expressed in prostate cancer and promotes the development of castration-resistant prostate cancer by causing differential splicing of EPHA6 [16, 17]. Furthermore, NONO expression is significantly increased in melanoma [18], gastric cancer cells [19], esophageal squamous cell carcinoma [20], and found to be associated with cancer aggressiveness. Proline (Pro)-directed serine or threonine (Ser/Thr-Pro) phosphorylation (pSer/Thr-Pro) is a typical signaling mechanism in cell proliferation and transformation [21]. The peptidyl-prolyl cis-trans isomerase NIMA-interacting 1 (PIN1) regulates the conformational changes of pSer/Thr-Pro motifs and causes alteration of the structure, function, and stability of numerous proteins [22, 23]. PIN1 is often dysregulated in various cancer types, and overexpression and/or overactivation is associated with a poor clinical prognosis [24, 25]. PIN1 overexpression has been shown to accelerate genomic instability and promote tumorigenesis by disrupting cell cycle coordination [26]. Ablation of PIN1 effectively suppresses tumor development in Neu or Ras transgenic mice [27]. Overactivation of PIN1 disrupts the balance between oncogenic and tumor-suppressing proteins, shifting it toward oncogenesis. In multiple cancers, more than 40 oncogenes are activated, and above 20 tumor suppressors are inactivated [21]. In our previous study, we used a web-based protein interaction network analysis platform called Relevance Rank Platform (RRP) to predict the functional relevance between the PIN1 oncoprotein and NONO [28]. However, the detailed mechanism by which PIN1 modulates NONO activity and promotes NONO-induced cell proliferation and transformation remains unclear. Many gene regulators driving oncogenesis are regulated by PIN1 [29], and NONO has recently emerged as a critical regulator in carcinogenesis [15]. NONO contains several Thr-Pro motifs and the PIN1 is known to bind phosphorylated Ser/Thr-Pro motifs in the target protein, this prompted us to identify specific Thr-Pro motifs in NONO protein that binds to PIN1. Our study showed that Thr-428–Pro-429 and Thr-450–Pro-451 of NONO specifically binds to the WW domain of PIN1. Binding of PIN1 promotes NONO stability and abundance of NONO and the activation of NONO-induced downstream signaling pathways involved in carcinogenesis. Our results unveil the role and molecular mechanism by which NONO contributes to the development of breast cancer. Given its crucial role in regulating the Akt/MAPK/β-catenin signaling and other cellular processes, NONO might be a potential therapeutic target for breast cancer.

## Materials and methods

### Cell culture, antibodies, and reagents

MDA-MB-231 and MCF-7 cells were purchased from NCCS Cell Repository (Pune, India) and in DMEM medium supplemented with 10% FBS, 100 units/ml penicillin (all from Gibco, ThermoFisher Scientific, USA) at 37 °C and maintained 5% CO_2_. Antibodies against NONO, PIN-1, β-catenin, Bax, Bcl-xl, Caspase-3, pP38, Cyclin E1, eIF4E, RACK1, NF-kB, and pNF-kB were obtained from Santa Cruz Biotechnology (Dallas, Texas, USA). The antibodies against PCNA, Vimentin, MMP-2, MMP-9, P53, and E-cadherin were purchased from Cloud-Clone Corp (Houston, USA). Anti-c-Jun and pc-Jun antibodies were obtained from Cell Signaling Technology (Danvers, Massachusetts, USA). Antibodies against β-actin and p-P53 and MG132 were obtained from Sigma-Aldrich (St. Louis, Missouri, USA). Matrigel was purchased from Corning Incorporated Lifescience (Tewksbury, MA, USA).

### Zebrafish lines and maintenance

Zebrafish (Danio rerio) were bred, reared, and maintained at 28.5 °C under standard conditions as described [30]. Embryos older than 24 hpf were reared in an embryo medium containing 0.003% phenylthiourea to prevent pigment formation for fluorescence imaging. Handling of Zebrafish was in strict accordance with the good animal practices outlined by the Committee for the Purpose of Control and Supervision of Experiments on Animals (CPCSEA), the Government of India. All experiments were approved by the Institutional Animal Ethics Committee (IAEC) of the Council for scientific and industrial research (CSIR) Institute of Genomics and Integrative Biology, New Delhi, India.

### Transfection of siRNAs

Pre-designed siRNAs (FlexiTube siRNA) were purchased from Qiagen, and the siRNA sequences used are listed in Table 1. The siRNA transfection experiments were conducted using forward and reverse transfection methods for MDA-MB-231 and MCF-7 cells, respectively. Briefly, MDA-MB-231 cells were seeded in a 6-well plate at a density of 2–3 × 10^5^ cells/well with complete DMEM medium. Once the cells reached 50–70% confluency, the media was replaced with Opti-MEM reduced serum media before transfection. A mixture of 4 µl of 20 µM siRNAs and 5 µl of Lipofectamine RNAiMAX (ThermoFisher Scientific, Waltham, MA, USA) in 100 µl of Opti-MEM media was incubated at room temperature for 30 min to form the complex. The resulting complex was added dropwise in each well containing cells with 1900 µl Opti-MEM reduced serum media at a final concentration of 40 nM siRNA. After 24 h of transfection, the medium was replaced with a fresh complete DMEM medium, and cells were collected for RNA and protein isolation 72 h post-transfection.

**Table 1:** Sequence of siRNAs

**siRNA**	**Scramble (scr)**	**NONO**
**Target (5ʹ-3ʹ)**	AATTCTCCGAACGTGTCACGT	AGGCTTGACTATTGACCTGAA
**sense (5ʹ-3ʹ) **	UUCUCCGAACGUGUCACGUdTdT	GCUUGACUAUUGACCUGAATT
**Antisense (5ʹ-3ʹ)**	ACGUGAGACACGUUCGGAGAAdTdT	UUCAGGUCAAUAGUCAAGCCT

In a reverse transfection, the transfection complex was added dropwise into each well of 6-well plate and 4 × 10^5^ MCF-7 cells in 2 ml of 10% FBS-contained DMEM medium was added. After 24 h of transfection the medium was replaced with fresh complete DMEM, and the cells were harvested after 72 h of transfection. Table 1 lists the siRNA sequences used in this study.

### Cell cycle

The distribution of the cell population in the different stages of the cell cycle was studied using siRNA-scr and siRNA-NONO-transfected MDA-MB-231 and MCF-7 cells. The cells were collected and washed with PBS after a 72 h transfection. Thereafter, the cells were fixed in 70% chilled ethanol and kept at 4 °C overnight. The next day the cells were centrifuged, ethanol removed, and two PBS washes performed. Thereafter, the cells were incubated with RNase (10 µg/mL) and propidium iodide (25 µg/mL), and kept at 37 °C for 30 min. FACS verse was used to evaluate the samples. A total of 10,000 events were acquired and the data were analyzed using ModFit LT software.

### Colony formation assay

MDA-MB-231 and MCF-7 cells were transfected with siRNA-scr and siRNA-NONO and their ability to form colonies was evaluated. Briefly, 5 × 10^2^ cells from the scramble and NONO knockdown cells were plated in each well of 6-well plates after the cells had been transfected for 24 h. The culture was kept for 12 days and the medium was replaced every 48 h. The cells were fixed with methanol and stained with 0.4 percent crystal violet. Image J software was used to count colonies.

### Wound healing assay

In each well of a 6-well plate, 4 × 10^5^ MDA-MB-231 and MCF-7 cells were seeded and transfected with siRNA-scr and siRNA-NONO. Once the monolayer had formed, a scratch was made with a 200 µl pipette tip. Detached cells were removed after gentle washing with PBS and the wells were replenished with fresh medium. The ability of the cells to migrate and close the wound area over time was monitored and the images were taken at different time points such 0 h, 12 h, 24, h and 48 h after creating the scratch. The analysis was performed with ImageJ. After normalizing the wound area, the % wound healing area was calculated.

### Migration and invasion assay

Migration and invasion potential of breast cancer cells was determined by using 8 µm pore size transwell chambers (Corning, NY, USA) placed in a 24 well plate. Briefly, 4 × 10^4^ siRNA transfected cells in 200 µl of serum-free medium were seeded into the upper chamber. 700 µl of DMEM supplemented with 10% FBS was added to the lower chamber to serve as a source of chemoattractant. The MDA-MB-231 and MCF-7 cells were allowed to migrate for 48 h and 72 h, respectively, at 37 °C and 5% CO_2_ and the non-migrated cells were gently scrapped off with wetted cotton swab. Cells that migrated to the lower surface of the upper chamber were fixed using methanol and stained with 0.4 percent crystal violet followed by washing to remove excess dye. The migrated cells were photographed using Nikon EclipseTi computerized image analyzing system. For invasion assay, the transwell chambers were pre-adhered with 80 µg Matrigel (Corning, NY, USA) diluted in 100 µl serum-free DMEM and incubated at 37 °C for 2 h. The other procedures were performed similarly as the migration assay.

### In vivo assay for analysis of metastasis in zebrafish embryos

2dpf (days post-fertilization) Tubingen, wild-type zebrafish embryos were dechorionated and anesthetized using 0.015 M tricaine (Sigma-Aldrich, Cat. No. A5040). The embryos were then oriented in a lateral position on a flat 2% agarose plate. The effect of NONO knockdown on the metastatic potential of MDA-MB-231 cells was evaluated by injecting embryos with an approximately equal number of (45–150) Green CM-FDA-labeled cells into the perivitelline space. Embryos were then collected and maintained in embryo media containing 0.003% in 1-phenyl-2-thiourea (Sigma-Aldrich, Cat. No. P7629) at 28 °C. Embryos were imaged at 72 hpi (h post-injections) using Nikon Eclipse Ti or Zeiss AxioScope A1 microscopes.

### RNA isolation and quantitative PCR

Total RNA was isolated from the siRNA-scr and siRNA-NONO-transfected MDA-MB-231 and MCF-7 cells using TRIzol Reagent (ThermoFisher Scientific, Inc.). The cDNA was prepared from 2 µg of total RNA according to the manufacturer’s instructions. The primers used to examine the expression of specific genes are listed in Table 2. According to the manufacturer’s instructions, cDNA was prepared from 2 µg of total RNA (ThermoFisher Scientific, Inc.). Table 2 lists the primers used against the genes examined. The PCR cycling schedule was 10 min at 95 °C, followed by 40 cycles of 15 s at 95 °C, 30 s at 60 °C, and a melting curve with a single reaction cycle at 95 °C for 15 s, 60 °C for 1 min and dissociation at 95 °C for 15 s. The resulting Ct values were then normalized using quantification of the housekeeping gene GAPDH. The relative expression of genes was determined using the 2^−ΔΔCt^ method.

**Table 2:** List of primers used for Real-Time PCR

**Gene**	**Forward primer sequence**	**Reverse primer sequence**
*NONO*	5ʹ- GGAGCCCATGGACCAGTTAG-3ʹ	5ʹ- AAATCTGGGTGGCTGCTCTC-3ʹ
*PIN1*	5ʹ- TTTGAAGACGCCTCGTTTGC-3ʹ	5ʹ-GTGCGGAGGATGATGTGGAT-3ʹ
*p21*	5ʹ-CTGCCCAAGCTCTACCTTCC-3ʹ	5ʹ-CGAGGCACAAAGGGTACAAGA-3ʹ
*GAPDH*	5ʹ- GTGAACCATGAGAAGTATGACAAC -3ʹ	5ʹ- CATGAGTCCTTCCACGATACC -3ʹ
*OCT4*	5ʹ-GAGAACCGAGTGAGAGGCAAC-3ʹ	5ʹ- CTGATCTGCTGCAGTGTGGGT-3ʹ
*SOX2*	5ʹ-TTTGTCGGAGACGGAGAAGC-3ʹ	5ʹ-TAACTGTCCATGCGCTGGTT -3ʹ
*Slug*	5ʹ- ACGCCTCCAAAAAGCCAAAC -3ʹ	5ʹ- ACTCACTCGCCCCAAAGATG -3ʹ
*Twist*	5ʹ-CTCGGACAAGCTGAGCAAGA-3ʹ	5ʹ-GCTCTGGAGGACCTGGTAGA-3ʹ
*Cyclin E1*	5ʹ-ATACTTGCTGCTTCGGCCTT-3ʹ	5ʹ- TCAGTTTTGAGCTCCCCGTC-3ʹ

### Crystal violet assay

The crystal violet assay was used to determine the viability of NONO-depleted breast cancer cells. MDA-MB-231 and MCF-7 cells were transfected with a Scramble and NONO-siRNAs in a 6-well plate and incubated for 24 h. The cells were then harvested and counted, and 5 X10^3^ cells were seeded into each well of a 96-well plate and incubated at 37 ˚C, 5% CO_2_ for the next 48 h. Thereafter, the medium was removed and the cells were stained with 0.4% crystal violet (prepared in 50% methanol) for 30 min. The wells were then cleaned with water to remove excess dye and air dried for 12 h. The next day, the dye was dissolved in 100 µL of methanol and the absorbance of the dissolved dye was measured at 570 nm. The viability of siRNA NONO w.r.t. siRNA scramble was determined as a fold change absorbance value.

### JC1 staining and flow cytometry

The effect of silencing of NONO on mitochondrial membrane potential was assessed with the JC-1fluorescent dye using a flow cytometer. MDA-MB-231 and MCF-7 cells were transfected with siRNA-scr and siRNA-NONO and incubated for 48 h at 37˚C and 5% CO_2_. Cells were harvested and stained with JC-1 dye for 30 min in the dark. The cells were then collected and washed with PBS and fluorescence was analyzed by flow cytometry (FACS Verse, BD).

### Flow cytometry-based detection of the intracellular ROS

MDA-MB-231 and MCF-7 cells were transfected with siRNA-scr and siRNA-NONO, incubated for 48 h before being trypsinized and resuspended in PBS with MitoSOX™ Red at a final concentration of 5 µM. The cells were then subjected to flow cytometry analysis to measure the intracellular ROS levels.

### Preparation of total cell lysate and western blot analysis

Preparation of whole cell lysates and the analysis of Western blots were performed as previously described [31]. An equivalent amount of cell lysate (30 µg) was electrophoresed on 8 ~ 15% SDS-PAGE gels, followed by electrotransfer to a PVDF membrane at 100 V for 1 h. The membrane was further blocked with 5% skim milk and incubated with primary and secondary antibodies in the dilutions suggested by manufacturers. ECL Western Blotting substrate Kit (Bio-Rad, Hercules, CA, USA) was used for the detection of horseradish peroxidase (HRP) and the signal was captured on X-ray film. ImageJ software was used for densitometry and the density of the protein of interest was normalized to that of β-actin using arbitrary densitometric units.

### Flow cytometric analysis of apoptosis

Annexin V-FITC and propidium iodide staining assay and FACS-verse analysis (BD, New Jersey, USA) were used to study the effect of NONO silencing on apoptosis in breast cancer cells. Breast cancer cells were transfected with the siRNA-Scramble and siRNA-NONO and incubated for 72 h at 37˚C, 5% CO_2_. The cells were then harvested and incubated for 30 min with a 1X binding buffer containing Annexin V and propidium iodide., The samples were then collected for FACS analysis (BD) to quantify the percentage of cells undergoing apoptosis.

### Lentiviral CRISPR construct generation

The guide sequences specific for targeting two different regions of the human *NONO* gene were designed as previously described [32]. Guide oligonucleotides containing the overhangs of the BsmBI restriction site overhangs were annealed for the current study according to the previously published protocol [33] and cloned into BsmBI linearized LentiCRIPRv2 vector (Addgene #52961). Correct insertion of guide sequence was confirmed using Sanger sequencing. The oligo sequences for sgRNA cloning are listed in Table 3.

**Table 3:** The oligo sequencing for sgRNA cloning used in the study

gRNA oligonucleotides		
sgRNAs	gRNA oligos	Sequence (5ʹ-3ʹ)
NONO gRNA1	F	CACCGCAATCCGTTCGACGACGACG
	R	AAACCGTCGTCGTCGAACGGATTGC
NONO gRNA2	F	CACCGCCTAGCGGAGATTGCCAAAG
	R	AAACCTTTGGCAATCTCCGCTAGGC
Control gRNA	F	CACCGAAACGGCGGATTGACCGTAA
	R	AAACTTACGGTCAATCCGCCGTTTC

### Lentivirus production and infection

Lentivirus particles were generated by co-transfection of 2 µg transfer plasmid LentiCRISPRv2 (Addgene #52961) or pLJM1-EGFP (Addgene #19319: positive control) with 1.5 µg packaging plasmid psPAX.2 (Addgene #12260) and 0.5 µg envelope plasmid pCMV-VSV-G (Addgene #8454) in a single well of a 6-well plate of 80% confluent HEK-293 T cells using 12 µl of Effectene transfection reagent (Qiagen, Germantown, USA). After 12 h of transfection, the medium was exchanged for complete DMEM. The virus-containing culture medium was harvested and after 48 h of transfection, centrifuged at 3,000 rpm at 4˚C for 10 min. The collected supernatant was filtered with a 0.45 µm filter membrane and used immediately.

Lentivirus infection was performed by mixing 1 ml cell suspension of 4 × 10^5^ cells and 1 ml polybrene (10 µg)/virus solution at a multiplicity of infection (MOI) <1. The plate was centrifuged at 931 g for 45 min at 30˚C. After centrifugation, the cells were incubated at 37˚C overnight in a tissue culture incubator. The following day, cells were seeded again in 10 cm dishes at a density of 100–500 cells/plate. For antibiotic selection, the MDA-MB-231 and MCF-7 cells were treated with 2 µg/ml and 0.5 µg/ml puromycin after 48 h of incubation. Cells were cultured in puromycin medium for 4–5 days until all the control cells died. The cells were further grown until single colonies were visible. Colonies were then picked up manually for single-clone expansion. Some surviving colonies in both cell lines are negative for NONO expression. Knockout of the NONO gene was then verified by western blotting.

### Yeast two-hybrid (Y2H) assays

The yeast two-hybrid assays were performed using the methods previously described [31]. The full-length human NONO cDNA was cloned into the pGADT7 plasmid and transformed into a Y187 yeast strain. In contrast, the full-length Huma PIN1 cDNA, the WW domain (amino acid, 1–138) of PIN1, or the segments of the PPIase domains (amino acid, 118–492) were cloned into pGBKT7 plasmids and transformed into Y2H Gold yeast strain. The yeast strains containing the desired plasmids were mated to produce diploid cells. The diploid cells were first selected on synthetically defined (SD) media dropped out for leucine and tryptophan (SD/-Leu/-Trp), followed by selection on quadra-dropout media restricted by leucine (L), tryptophan (W), histidine (H), and adenine (A) (SD/-Leu/-Trp/-His/-Ade) for positive interaction.

### Generation of silent mutations and rescue of the knockdown effect

The three silent mutations were introduced into the siRNA targeting region of the wild-type human NONO expression plasmid using the methods previously described [31]. Briefly, the full-length NONO cDNA was cloned into pCDNA3.1(+) plasmid (Invitrogen, Waltham, MA), followed by the insertion of three silent mutations using QuikChange II Site-directed mutagenesis kit (Agilent, Santa Clara, USA) into the siRNA targeting region (Δ3) of the wild-type NONO expression plasmid to rescue the siRNA-induced phenotype. Silent mutations were verified by DNA sequence analysis.
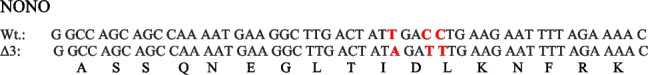


The MDA-MB-231 cells were transfected with siRNA for the knockdown-rescue experiment. The siRNA-resistant NONO overexpression plasmid was transfected using cells after 24 h of siRNA transfection by Lipofectamine LTX ((ThermoFisher Scientific, Inc.) The serum-free medium was replaced with complete medium for 6 h after transfection, and the cells were cultured for 48 h to assess NONO knockdown rescue. The level of protein was examined by Western blotting.

### FRET imaging

FRET analysis was performed to evaluate the interaction of PIN1 or its domains with NONO. First, the NONO sequence was amplified from pCDNA 3.1(+)-NONO (containing the complete human NONO sequence, codon 1 to 471) using Q5 polymerase (NEB, Ipswich, MA). The amplified NONO sequence was then cloned into mVenus-C1 plasmid (Addgene #27794) using Infusion HD cloning kit (Takara, Shiga, Japan). The same procedures were used to clone the full-length human PIN1 sequence (codon 1 to 163) or PIN1WW domain (codon 1 to 46) and PIN1PPIase domain (codon 40 to 163) done in pmTurquoise-C1 plasmid (Addgene #60558). The constructs were verified by Sanger sequencing.

FRET experiments were performed using PFA-fixed HEK293T cells mounted on a 24-well plate with 20 mm round coverslips and transfected with dual expression plasmids: (i) mVenusC1-NONO and pmTurquoiseC1-PIN1, (ii) mVenusC1-NONO and pmTurquoiseC1-PIN1-WW domain, (iii) mVenusC1-NONO and pmTurquoiseC1-PIN1-PPIase domain, (iv) two plasmids mVenusC1/pmTurquoiseC1 containing unfused mVenus (yellow) and Turquoise (cyan) expressing proteins. First, fluorescence intensities of mVenus and mTurquoise were observed. The region of interest (ROIs) was then determined and the acceptor photobleaching FRET assay between the fluorophores (mTurquoise and mVenus) was performed using inverted confocal laser scanning NIKON microscopy with *NIS* Elements software, objective lens, and filters for CFP (excitation 405 nm and emission 477 nm/27 nm bandwidth) and YFP (excitation 515 nm and emission 527/48 nm bandwidth). Images are initially acquired with both CFP and YFP channels for the acceptor photo-bleach FRET assay. The YFP partner is then photobleached using the high intensity laser at 514 nm for 20 s, after which the images were reacquired with both CFP and YFP channels. The FRET energy transfer efficiency (E) was calculated from the following equation: E = 1-F_pre_/F_post_, where F_pre_ and F_post_ are the donor fluorescence intensities within the ROI before and after bleaching, respectively.

### Immunoprecipitation

Magnetic dynabeads protein G (ThermoFisher Scientific) were washed and blocked with PBS supplemented with 5% BSA. The beads were resuspended in 200 µl PBS/BSA and relevant antibodies (mouse IgG control and anti-NONO) were added, followed by rotation at room temperature for 2 h. The bead-antibody complex was washed three times with PBS/BSA solution using the magnetic tube holder and the supernatant was removed. Cells were lysed with non-denaturing lysis buffer (50 mM HEPES, 150 mM NaCl, 1% NP-40, 2 mM EDTA, 0.2 mM PMSF, 1 mM DTT, 5% glycerol) at 4 ˚C for 10 min with gentle rolling. Cells were sonicated 3 times for a 5-s pulse each and centrifuged at 10,000 g for 20 min at 4˚C. The concentration of protein was determined using a BCA kit (ThermoFisher Scientific, Waltham, MA, USA). 40 µl of lysate was collected for input and 700 ug (300 µl) of lysate was added to the bead-IgG and bead-primary antibody complex and incubated overnight at 4 ˚C with gentle rotation. The next day, the bead-antibody with precipitated protein was washed 5 times with lysis buffer, rotating for 1 min at 4˚C for each wash. The bead-antibody complexes were resuspended in 2xSDS lysis buffer and boiled together with the input at 100 ˚C for 5 min. The supernatant was transferred to fresh tubes for immunoblotting analysis using a magnetic separator rack.

### Immunohistochemistry (IHC) analysis

After deparaffinization, hydration, and washing, tissue sections were immersed in Tris–EDTA buffer (pH 9) at boiling temperature for 15 min for antigen retrieval. The slides were immersed in 3% H_2_O_2_ for 20 min to block endogenous peroxidase reaction. Slides were washed with TBST buffer and blocked with 3% BSA for 20 min and then incubated with anti-NONO and anti-PIN1 primary antibodies diluted in blocking buffer at 4˚C overnight. Slides were processed with the EnVision+ Dual Link HRP System kit (DAKO, Agilent, CA) and counterstained with hematoxylin according to the manufacturer’s instructions. All IHC data were evaluated and scored from 1 to 3 based on the intensity of expression. The pathological scoring was performed using the quick score method [34] and the intensity of immunopositivity (stained) cells within the tissue section was scored in the range of 1 to 3. The expression intensity score was multiplied by the percentage of stained cells (denoted as 3 if the percentage of stained cells was >50%, 2 if the percentage of the stained cells was 25–50%, and 1 if the percentage of the stained cells was <25%) that defined the weak, intermediate and strong protein expression.

### Immunofluorescence (IF) analysis

The cells were seeded on the cover slips at an appropriate confluency of 60%. After 24 h of seeding, the cells were transfected with siRNA-scr or siRNA-NONO. 72 h after transfection, the cells were fixed with 4% para-formaldehyde (PFA) for 10 min at room temperature and washed three times with ice-cold PBS for 5 min each. Cells were permineralized with 0.2% Triton X-100 and blocked in 1% BSA, 22.52 mg/ml glycine in PBST for 1 h at RT and incubated in the diluted antibody in 1% BSA in PBST (1: 100) in a humidified chamber overnight at 4 ˚C. The cells were then incubated in goat anti-Mouse IgG-FITC secondary antibody solution (Thermo, 1:250 in PBST) for 30 min at room temperature and counter-stained with 1 µg/ml DAPI for 1 min. The fluorescence was then observed under a Nikon Eclipse Ti microscope.

### Bioinformatic analysis

OMICS data of breast tumors and normal tissues were analyzed using the Tumor Immune Estimation Resource TIMER (https://cistrome.shinyapps.io/timer/), UALCAN (http://ualcan.path.uab.edu/), and TNMplot (https://tnmplot.com/) web tools.

### Statistical analysis

Statistical analysis was performed using GraphPad Prism (GraphPad Software, San Diego, CA, USA) and Microsoft Excel; data were presented as mean ± SD. The unpaired two-tailed t-test or ANOVA test was used to compare different groups. The correlation between PIN1 and NONO was determined using Spearman’s rank correlation. The Mann-Whitney test was used to compare rapid scores between paired breast cancer and adjacent tissues and for tracking measurements. A *p*-value less than 0.05 was considered statistically significant.

### Availability of data and materials

All data generated or analyzed during this study are included in this article and its supplementary information files.

## Results

### Breast cancers have the elevated expression of NONO

**Fig. 1:**
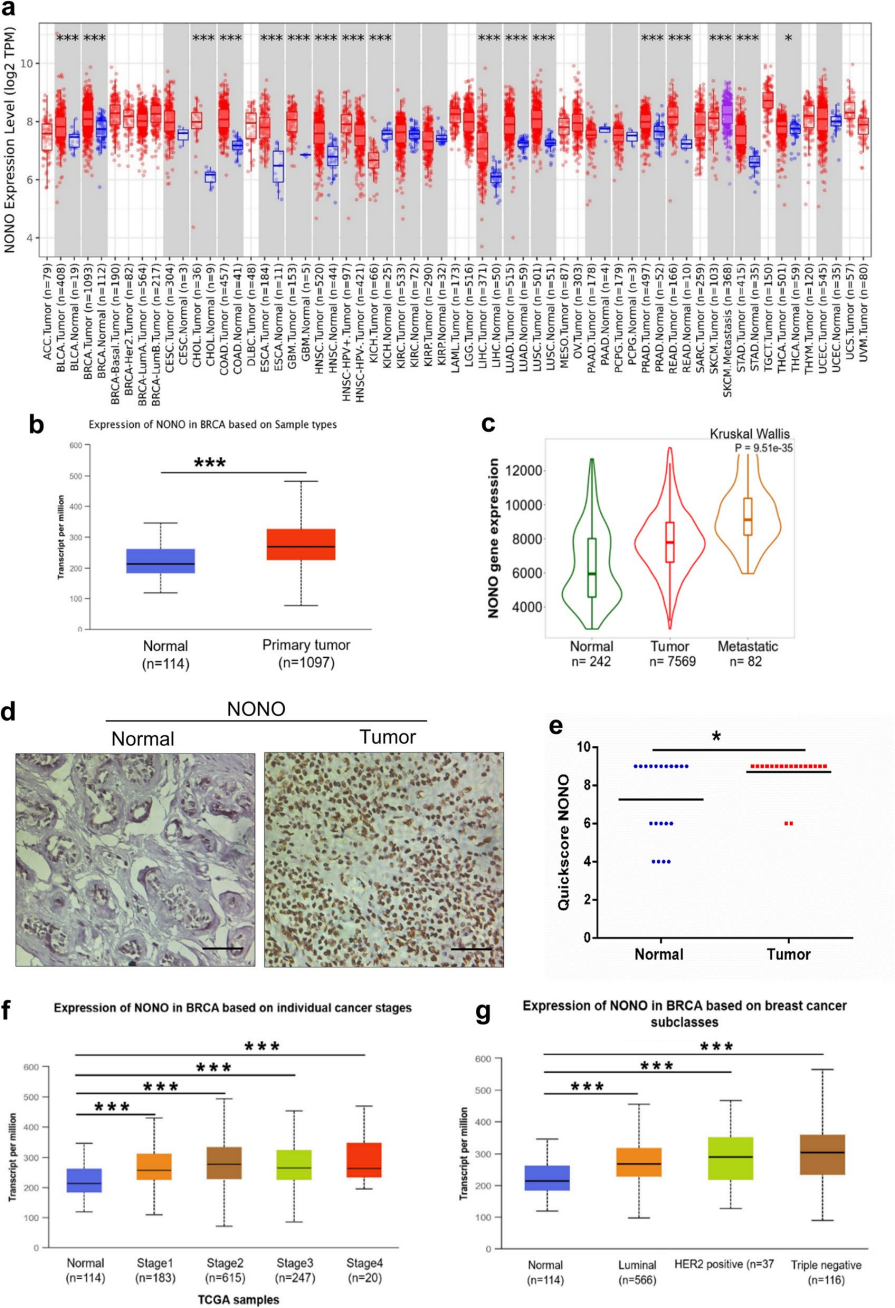
NONO is significantly expressed in breast cancer. **a** Expression levels of NONO in different cancers types and their respective normal tissues according to data retrieved from the TIMER database. **b** TCGA data from UALCAN suggested that NONO expression was significantly higher in breast cancer than in normal breast tissue. **c** NONO gene expression profile in normal breast tissue, breast tumor and metastatic breast tissue. Using the TNMplot web platform. **d** Representative IHC image of NONO protein expression in breast cancer tissue and adjacent normal tissue. Scale bar, 50 µm. **e** Quick score distribution of NONO in breast cancer tissue (*n* = 20) versus normal tissue (*n* = 20). **f**, **g** According to TCGA data from UALCAN, NONO is significantly overexpressed in different stages and subclasses of breast cancer. *p < 0.05, ***p < 0.001

To study the differential expression of NONO between tumor and normal tissue, the cancer genomic atlas program (TCGA) data expression analysis, was performed using the tumor IMmune estimation resource (TIMER) web tool. NONO expression was found to be significantly upregulated in several cancer types, including breast cancer (Fig. 1a). The data retrieved from TCGA using UALCAN showed higher mRNA expression in breast cancer tissue than in normal breast tissue (Fig. 1b). Mining of gene chip data from Gene Expression Omnibus (GEO) or RNA-seq data from TCGA, Therapeutically Applicable Research to Generate Effective Treatments (TARGET), and the Genotype-Tissue Expression (GTEx) databases with TNMplot web toll or gene chip data from GEO revealed a significant regulation of NONO in primary and metastatic carcinoma than in normal tissue (Fig. 1c).

To confirm the increased expression of NONO in breast cancer tissues as compared to adjacent normal tissues by immunohistochemistry (IHC), we first validated the specificity of antibodies by the siRNAs knockdown strategy. A single band of appropriate size with significantly reduced signal in siRNA-knockdown cells on immunoblots confirmed the specificity of antibodies (Supplementary Fig. S1A, B). The combination of siRNA knockdown strategy with immunofluorescence microscopy further validated the specificities of antibodies against NONO and PIN1 proteins (Supplementary Fig. S1C, D). Immunohistochemical analysis of patient tissue samples confirmed a significant increase in the expression of NONO protein in breast cancer tissues compared to their adjacent non-cancerous tissues (Fig. 1d, e). Immunohistochemical analysis of NONO protein expression further confirmed a significant increase in NONO expression in breast cancer tissues compared to the adjacent normal tissues in patient samples (Fig. 1d, e). These results indicated that NONO expression is upregulated in breast cancer and may play an essential role in breast cancer progression.

Next, the expression of NONO in the patients was examined based on various clinical parameters using the UALCAN online database. UALCAN database demonstrated that NONO expression levels of mRNA and protein were significantly upregulated across all the stages (stage 1–4) and subclasses (Luminal, HER2 positive, and TNBC) of breast cancer (Fig. 1f, g, supplementary Fig. S2A-C) These results showed that NONO expression and tumor progression are closely correlated and highlighting the potential of NONO as a promising target for the development of therapeutic strategies..

### NONO interacts specifically with PIN1, and this interaction is dependent on the N-terminal (WW) domain of PIN1

**Fig. 2:**
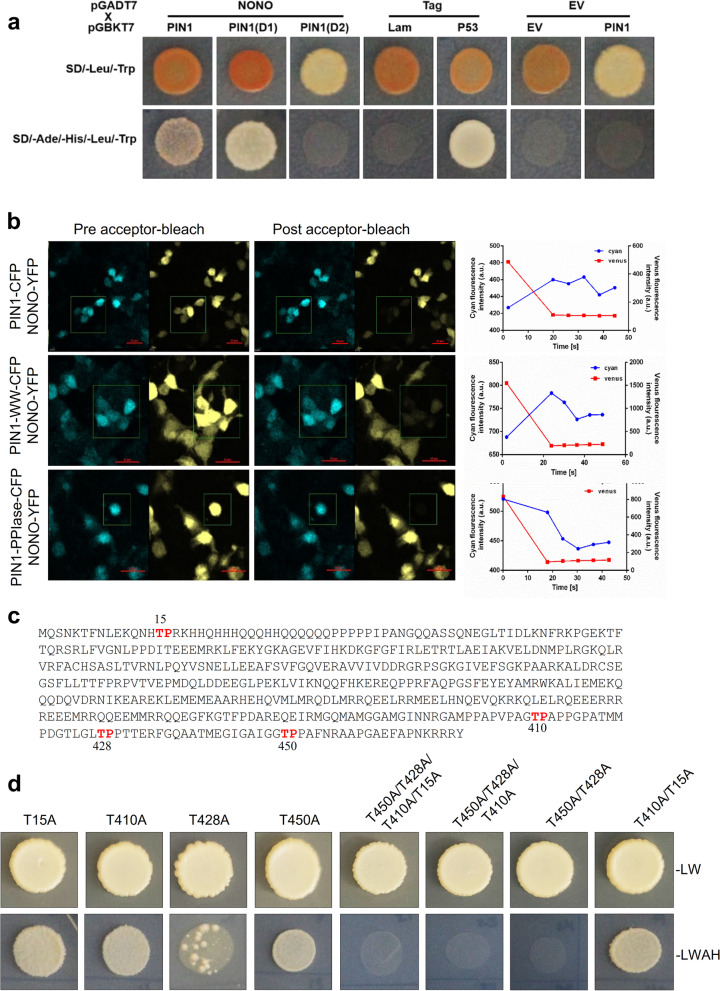
C-terminal thr-pro motifs in NONO specifically interact with the WW domain of PIN1. **a** Yeast two-hybrid analysis shows the protein-protein interactions of NONO with the PIN1 and PIN1-WW domain. The yeast strains containing bait and prey plasmids were mated. The diploids were selected on double-dropout medium –LT (SD-Leu/-Trp) and quadra-dropout medium –LTHA (SD/-Leu/-Trp/-Ade/-His) for yeast two-hybrid screening. P53 BD/T antigen AD and Lam BD/T antigen AD was used as positive and negative controls. **b** Detection of FRET by acceptor photobleaching. HEK293T cells were co-transfected with PIN1-CFP/NONO-YFP, PIN-WW-CFP/NONO-YFP, PIN1-PPIase-CFP/NONO-YFP. After 48 h of transfection, the cells were fixed, the images of CFP and YFP were acquired using CFP and YFP channels, and the intensities were measured before and after acceptor photobleaching. Scale bar 25 µm. The bar graph depicting the FRET efficiency of various cyan/yellow pairs. Data are presented as mean ± standard deviation with *n* ≥ 3. p* < 0.05. **c** Presence of four the pro motifs in NONO ORF (CCDS14410.1). *Source*: National Canter for Biotechnology Information (https://www.ncbi.nlm.nih.gov/). **d** Site-directed mutagenesis identifies the thr-pro motifs essential for the interaction of NONO with the PIN1 WW domain. The effect of substituting threonine with glycine at thr-pro motifs within the NONO ORF was analyzed by yeast two-hybrid screening. The two thr-pro motifs at the C-terminus of NONO are crucial for the interaction of NONO with the PIN1 WW domain

In the previous study [28], NONO is ranked among the top proteins with functions similar to those of peptidylprolyl cis/trans isomerase (PIN1). PIN1 is an oncoprotein that is often associated with cancer and PIN1’s function is defined by its two domains: the WW domain and the PPIase domain. It has been shown that the WW domain has a stronger affinity for its substrate than the PPIase domain [35] and the binding of the WW domain to the substrate allows PIN1 to perform molecular functions through the PPIase domain [36]. Previously, the interaction between PIN and NONO was revealed in vitro [37]. Using an independent yeast two-hybrid assay, we confirmed the interaction between human NONO and PIN1. We identified the functional WW domain (1–138 amino acids) of PIN1, which is essential for direct interaction with NONO (Fig. 2a).

The interaction of NONO with PIN1 and the WW domain of PIN1 was confirmed, specifically with the WW domain of PIN1 using the acceptor photobleaching FRET technique. During acceptor photobleaching, the intensities of CFP (FRET donor: cyan fluorescence protein) and YFP (FRET acceptor: yellow fluorescence protein) were monitored, and the images were taken before and after photobleaching of YFP within a region of interest (ROI) recorded. It was observed that for the FRET pair NONO-YFP/PIN1-CFP and NONO-YFP/PIN1-WW-CFP, the CFP intensities increased at multiple time points after acceptor photobleaching. In contrast, no increase in CFP intensity was observed for NONO-YFP/PIN1-PPIase pair (Fig. 2b, Supplementary Fig. S3A-C). As shown in Fig. 2b right panel, the average FRET efficiency was 7.72% and 12.63% in bleaching (post-bleaching) in PIN1-CFP/NONO-YFP and PIN1-WW-CFP/NONO-YFP samples respectively compared to control (samples with empty CFP and YFP vectors). As expected, the FRET efficiency of samples co-transfected with PIN1-PPIase-CFP/NONO-YFP showed no increase in CFP fluorescence intensity, further confirming that the WW domain but not the PPIase domain of PIN1, is essential for direct interaction with NONO.

Since the human NONO ORF (CCDS14410.1) has four threonine-proline (thr-pro) sites (Fig. 2c), we attempted to identify the thr-pro binding site(s) responsible for interacting with of the PIN1-WW domain are crucial. Four threonine residues at positions 15, 410, 428 and 450 of NONO were replaced with glycine, and the yeast two-hybrid was performed. Our results showed that a single mutation did not abolish the interaction of NONO with the PIN1WW domain. However, reduced growth in response to protein-protein interaction was observed in yeast diploids harboring the T428A substitution mutation, indicating a moderate affinity of Thr_428_-Pro_429_ for the WW domain of PIN1. Next, the substitution of glycine for threonine residues at all four thr-pro sites completely abolished the NONO interaction with the PIN1WW domain.

We also analyzed thr-pro motifs that are significant for the interaction of NONO with the PIN1WW domain. No interaction was observed between NONO-containing T410A, T428A, and T450A mutations and the PIN1-WW domain. Substitution mutationsT410A and T15A generated the mutant NONO protein that is still able to interact with the PIN1WW domain. However, the substitution of T428A and T450A completely abolishes the interaction of NONO with the PIN1WW domain, indicating that two thr-pro binding sites on the C-terminal of NONO protein are required for the interaction of NONO with the WW domain of PIN1 are essential (Fig. 2d). These findings suggest that the interaction between PIN1 and NONO may be important for regulating breast cancer progression, potentially through modulating the function of NONO protein.

### The stability of NONO is regulated by PIN1

**Fig. 3:**
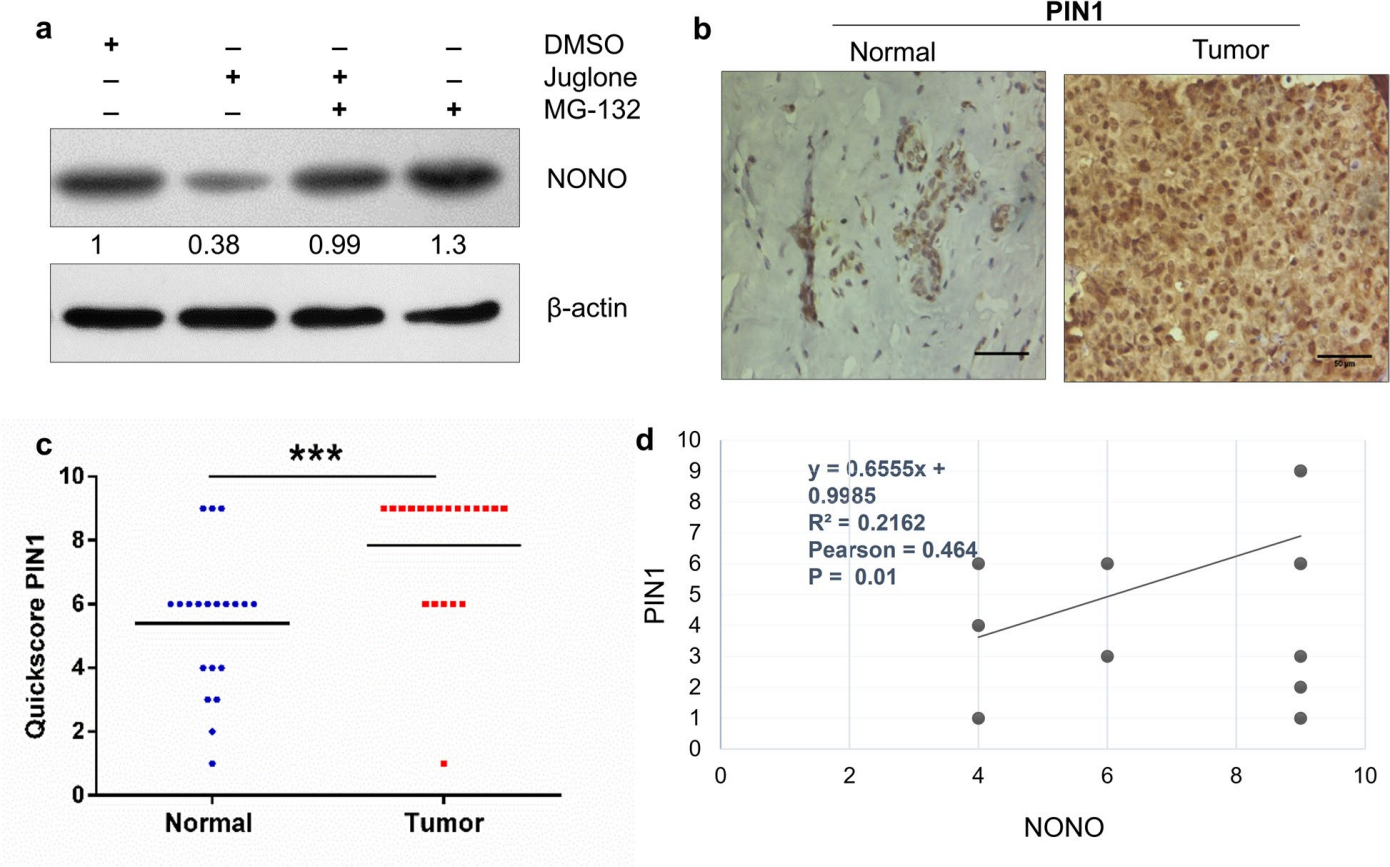
The stability of NONO protein is enhanced by PIN1. **a** MDA-MB-231 cells were treated with juglone or DMSO. About 48 h later, the cells were treated with MG132 for 8 h. Western blot analysis revealed the involvement of PIN1 in NONO protein abundance and regulation via the proteasomal inhibition pathway. β-actin was used as an internal control and ImageJ software for densitometry analysis. **b** Representative IHC image of PIN1 protein expression in breast tumors and normal tissue. Scale bar, 50 µm. **c** The distribution of PIN1-IHC signals in breast and normal tissue was analyzed using the quick score method. The unpaired Mann-Whitney test was used to compare IHC quick scores in tumor tissue with versus normal. **d** A significant association (Spearman’s rank correlation coefficient *r* = 0.464, *p* = 0.01, *n* = 20) between NONO and PIN1 proteins was observed in breast cancer. p* < 0.05, ***p < 0.001

To determine the effect of PIN1 inhibition on NONO, we used the PIN1 inhibitor juglone [38]. Since NONO binds directly to PIN1, we then asked whether the protein-protein interaction between NONO and PIN1 promotes NONO stability. For this, we treated the MDA-MB-231 cells with Juglone and examined the effect of the proteasome inhibitor MG-132 in juglone-treated cells. Our results revealed that treatment with the PIN1 inhibitor Juglone led to a decrease in the levels of NONO protein and this effect was reversed with the treatment of proteasome inhibitor MG132 (Fig. 3a). This indicated that PIN1-mediated stabilization of NONO occurs through the inhibition of proteasomal degradation.

Since the expression of NONO protein was upregulated in human breast cancer tissues (Fig. 1d) and its binding with PIN1 promotes its stability. Next, we performed the IHC to examine the expression of the PIN1 protein in the same groups of human breast cancer sections in which IHC NONO was carried out. We observed a significant increase in PIN1 protein expression in breast cancer tissues (14 out of 20, 70%) compared to normal adjacent tissues (Fig. 3b, c). Statistical analyses further revealed a significant correlation (Spearman’s Rank Correlation (r_s_) = 0.4645, *P* = 0.01) between PIN1 and NONO (Fig. 3d) in human breast cancer tissues, indicating that binding of PIN1 to NONO is involved in the oncogenic behavior of breast cancer cells.

### Silencing of NONO gene expression decreases breast cancer cell viability and colony-forming ability

**Fig. 4:**
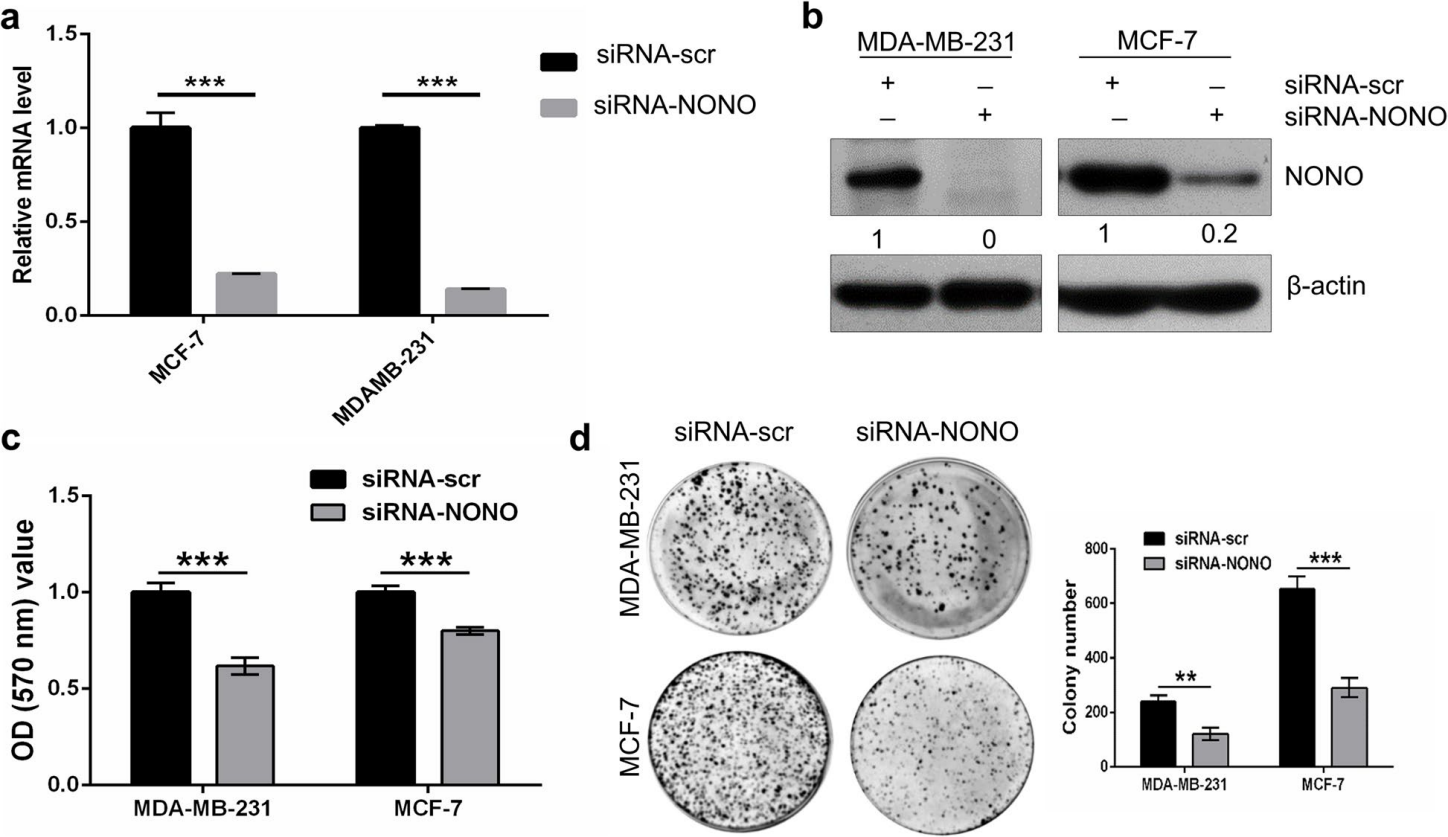
Silencing of NONO inhibits the tumorigenesis and colony formation ability of MDA-MD-231 and MCF-7 breast cancer cells. **a**, **b** siRNA-NONO is efficient in knocking down the NONO gene at the transcriptional level and the corresponding protein level, as shown by RT-PCR and Western blot, respectively. **c** Crystal violet assay demonstrated the proliferative ability of breast cancer cells transfected with siRNA-NONO and siRNA-scr. **d** The clonogenicity of NONO-silenced breast cancer cells was determined by colony formation assay. The bars show the mean standard deviation ± SD of at least triplicate replicates. **p < 0.01, ***p < 0.001

To gain fundamental insights into the function of the NONO gene, we first examined the knockdown efficiency of siRNA-NONO at the transcription level of the target gene and the corresponding protein level using RT-PCR and Western blot, respectively. siRNA-NONO was shown to efficiently reduce the mRNA and protein levels of the NONO gene in both MDA-MB-231 and MCF-7 cells (Fig. 4a, b). Next, the rescue experiment was performed to determine the specificity of siRNA. NONO human cDNA was cloned into pcDNA3.1(+), and three silent mutations were introduced into NONO ORF by site-directed mutagenesis to make the construct resistant to siRNA (supplementary Fig. S4a, b). Transfection of exogenous mutant NONO constructs rescued the expression of NONO and PCNA when co-transfected with the corresponding siRNA NONO (Supplementary Fig. S4c), indicating that the siRNA is highly selective and specific for the endogenous NONO gene. In addition, the expression profile of the NONO protein was observed in different breast cancer cells. Western blot analysis revealed that NONO expression was particularly overexpressed in human breast cancer cell lines MCF-7, MDA-MB-231, T47D, and MDA-MB-453 compared to normal human mammary epithelial cells (MCF10A). The result suggests that NONO is crucial in the development of human breast cancer (Supplementary Fig. S5).

The oncogenic behavior of breast cancer cells was analyzed after knocking down NONO gene expression using crystal violet and colony formation assays. The crystal violet assay showed a significant decrease in the viability of siRNA-NONO-transfected MDA-MB-231 and MCF-7 cells compared to siRNA-scr after 72 h of transfection (Fig. 4c). Next, we examined the colony-forming ability of NONO-silencing breast cancer cells. Compared to siRNA-scr-transfected breast cancer cells, NONO-knockdown MDA-MB-231 and MCF-7 showed reduced colony-forming ability as demonstrated by the colony-forming assay (Fig. 4d). These results suggest the involvement of NONO in regulating the tumorigenic properties of breast cancer cells.

### Silencing of NONO induces the S-phase cell cycle arrest in breast cancer cells

**Fig. 5:**
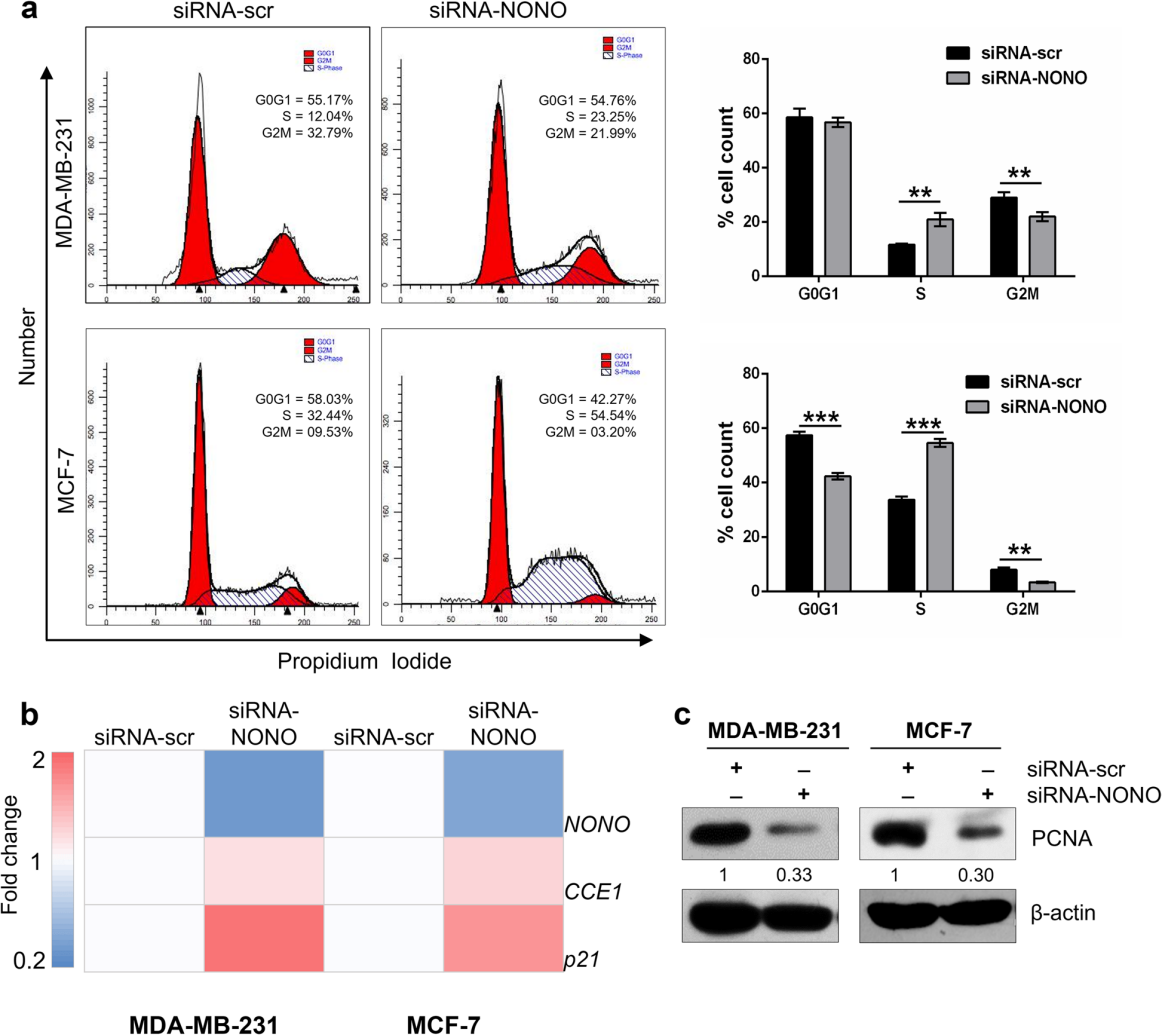
Silencing of NONO gene expression induces S-phase cell cycle arrest in MDA-MB-231 and MCF-7 breast cancer cells. **a** silencing of NONO induced accumulation of cells in the S phase as shown by flow cytometry. **b** Quantification of cell cycle-regulating genes using qRT-PCR in MDA-MB-231 and MCF-7 cells. NONO silencing affects the expression of cell cycle-related genes. CCE1 (CyclinE) was significantly downregulated while CDKN1A (P21) was strongly upregulated in MDA-MB-231 and MCF-7 breast cancer cells. **a**, **b** Data presented as mean standard deviation ± SD of three independent experiments. p* < 0.05, **p < 0.01, ***p < 0.001. **c** Western blot analysis of PCNA in control and NONO-silenced MDA-MB-231 and MCF-7 cells. PCNA expression was inhibited in both cell lines. β-actin was used as an internal control, ImageJ software for densitometry analysis

Flow cytometry was used to analyze cell cycle phase distribution in siRNA-transfected breast cancer cells to investigate the effect of NONO knockdown on cell cycle progression. Compared to siRNA-scr transfected cells, the silencing of NONO significantly arrested the cell cycle at the S-phase and decreased the percentage of cells in the G2/M phase in MDA-MB-231 cells, as well as in G0/G1 and G2/M phase in MCF-7 cells (Fig. 5a). After cell-cycle distribution analysis, we examined the effect of the silencing of NONO on the expression of cell cycle-related markers. Real-time PCR analysis showed that silencing NONO significantly increased cyclin E and CDKN1A (p21) mRNA expression levels (Fig. 5b). p21 has the unique ability to inhibit cyclin-dependent kinase, block PCNA-dependent DNA replication, and cause arrest in the S phase of the cell cycle [39]. Our results showed that at the protein level, expression of PCNA was reduced in both MDA-MB-231 and MCF-7-NONO knockdown cells (Fig. 5c). Taken together, the above results suggest that the accumulation of NONO-silenced cells in the S phase may be due to induction of p21 and inhibition of PCNA.

### Silencing of NONO induces apoptosis and promotes changes in mitochondrial membrane potential

**Fig. 6:**
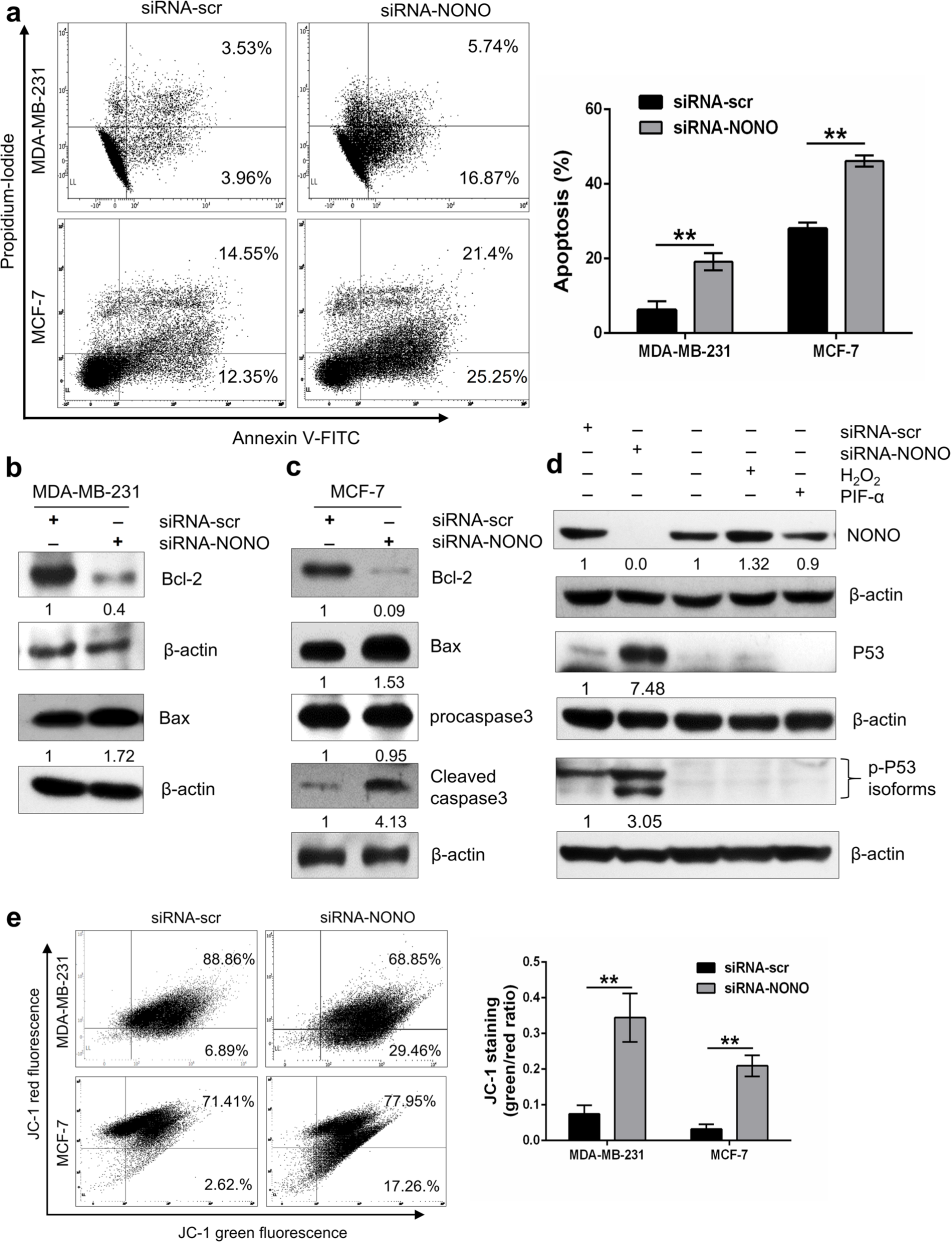
Silencing of NONO induces apoptosis in MDA-MB-231 and MCF-7 cells. **a** PI and Annexin V staining of NONO-depleted MDA-MB-231, and MCF-7 cells were determined by flow cytometry after 72 h of transfection. The percentage of apoptotic cells was plotted for MDA-MB-231 and MCF-7 cells. **p < 0.01. **b**, **c** Expression of Bcl-2 and Bax in MDA-MB-231 and MCF-7 and procaspase 3 and cleaved caspase 3 in MCF-7 were analyzed by Western blot analysis. **d** Expression of the apoptotic proteins p53 and p-p53 in NONO-depleted MCF 7 cells. Treatment with 50 µM H_2_O_2_ for 6 h promotes NONO expression, while treatment with the p53 inhibitor PIF-α for 24 h does not affect NONO expression. **e** The effect of NONO silencing on mitochondrial membrane potential alteration (MMP) by JC-1. **a**, **e** Data presented as mean ± SD of three independent experiments. **p < 0.01. **b**, **c**, **d** Representative blots from at least two independent experiments are shown. β-actin was used as an internal control

To analyze the impact of NONO silencing on cell death by apoptosis, flow cytometric analysis was performed using an annexin-V/propidium-iodide assay. A significant increase in the apoptosis index in breast cancer cells with knockdown of NONO was observed in both MDA-MB-231 and MCF-7 cell lines compared to siRNA-scr-transfected cells (Fig. 6a). After 72 h of transfection, the percentage of apoptotic cells increased to 22.61 ± 1.8 percent in MDA-MB-231 and 46.1 ± 1.19 percent in MCF-7 cells, while the siRNA-scr transfected cells showed a lower percentage of apoptotic cells, with 7.49 ± 1.9 percent in MDA-MB-231 cells and 26.8 ± 1.4 percent in MCF-7 cells. In addition, we examined the effect of NONO knockdown on the expression of apoptosis-regulating molecules; Western blotting analysis showed that NONO gene silencing effectively increased the level of pro-apoptotic Bax in MDA-MB-231 and MCF-7 cells and cleaved caspase 3 in MCF-7 cells while reducing the level of anti-apoptotic Bcl-2 proteins in both MDA-MB-231 and MCF-7 cells after 72 h of transfection (Fig. 6b, c). We, next determined the effect of NONO silencing on the expression of p53 in MCF-7 cells. It was observed that the silencing of NONO increased the expression of p53 and p-p53 proteins. Furthermore, treatment of MCF-7 cells with the p53 inhibitor pifithrin-α does not affect NONO expression. In contrast, treatment of MCF-7 cells with H_2_O_2_ at a concentration of 50 µM for 6 h induces the expression of NONO (Fig. 6d). This suggests that NONO may be a new drug target for selectively promoting p53-mediated cell cycle arrest and apoptosis in breast cancers with wild-type p53.

Furthermore, we used flow cytometry to study the effect of NONO depletion on the change in mitochondrial membrane potential of MDA-MB-231 and MCF-7 cells using the JC-1 dye. It was shown that silencing NONO in MDA-MB-231 and MCF-7 caused a reduction in mitochondrial membrane potential as seen by an increase in the green-to-red ratio(monomer/aggregate) compared to scramble. After the knockdown of NONO in MDA-MB-231, the JC-1 green/red ratio was significantly increased from 0.07 ± 0.05 to 0.344 ± 0.01 in a scrambled group. Similarly, upon NONO knockdown in MCF-7 cells, the green/red ratio of JC-1 was increased from 0.03 ± 0.01 to 0.20 ± 0.02 in the scrambled group. These results suggest that NONO silencing causes mitochondrial dysfunction by modifying the mitochondrial membrane potential (MMP) (Fig. 6e). Since the loss of mitochondrial membrane potential (ΔΨm) during redox stress is associated with ROS-induced apoptosis. We examined the effect of silencing NONO on ROS generation using the red fluorescent dye MitoSOX by flow cytometry. It was observed that NONO depletion significantly increased ROS potency in MDA-MB-231 cells compared to scramble (Supplementary Fig. S6), indicating that NONO prevents apoptosis by inhibiting ROS production.

### Silencing of NONO reduces the migratory and invasive potentials of breast cancer cells

**Fig. 7:**
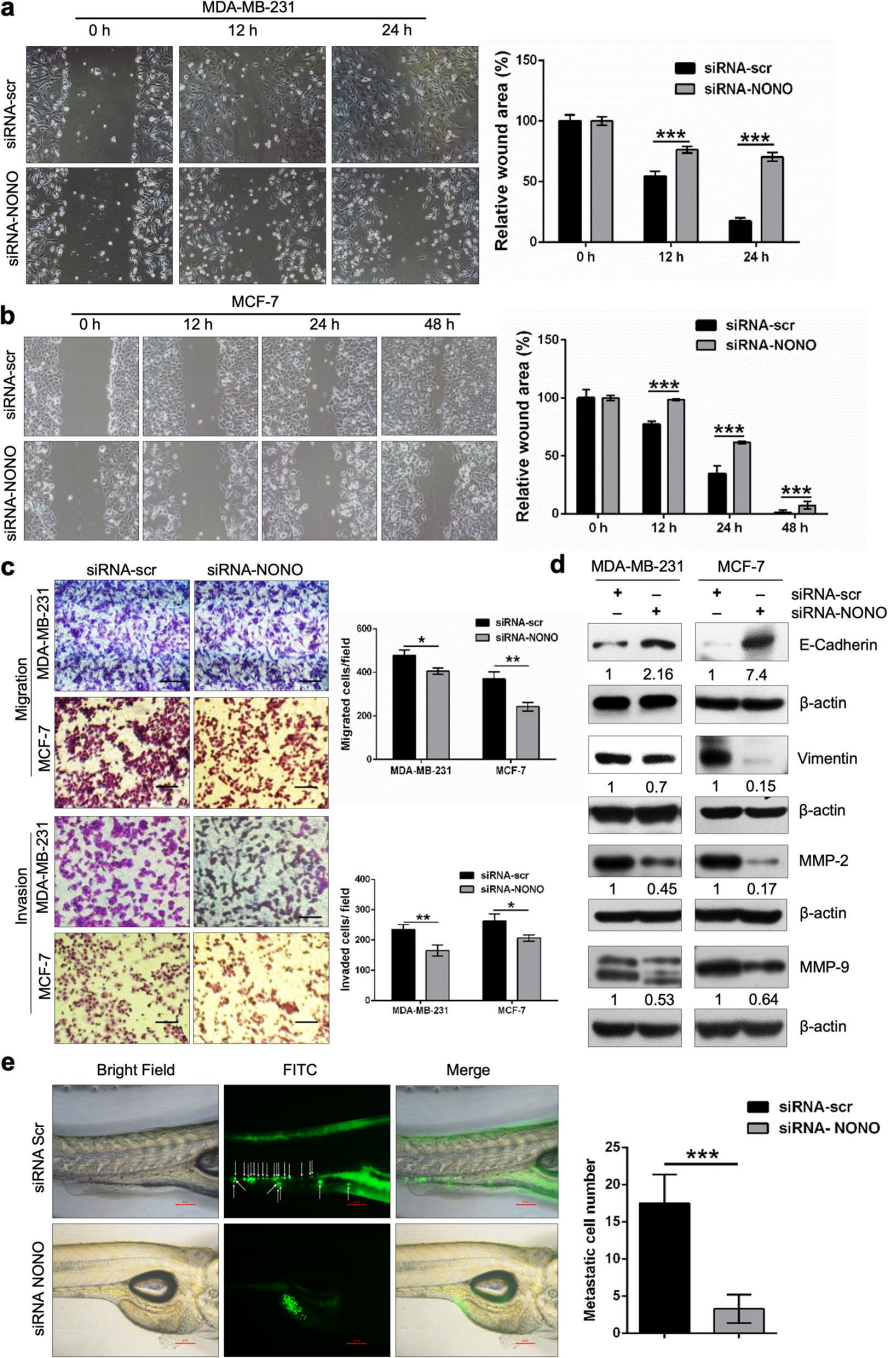
NONO knockdown inhibits the migration and invasion potential of breast cancer cells *invitro* and in vivo. **a**, **b** The migration ability of siRNA-scr and siRNA-NONO transfected breast cancer cells was investigated using a wound healing assay. ImageJ 64 was used to measure wound area. Magnification, 10X. **c** The Transwell Migration and Invasion Assay determined the migratory and invasive capacities of MDA-MB-231 and MCF-7 breast cancer cells transfected with siRNA-scr and siRNA-NONO. Scale bar, 100 µm and magnification, 20X. **a**-**c** Data are representations of three independent experiments. *p < 0.05, **p < 0.01, p*** < 0.001. **d** Western blot analysis of the expression of E-cadherin, vimentin, MMP-2 and MMP-9 proteins in the siRNA-scr and siRNA-NONO-transfected breast cancer cells. β-actin served as a protein loading control. The epithelial marker E-cadherin was upregulated, and the mesenchymal marker vimentin was downregulated in NONO knockdown cells. Endogenous MMP-2 and MMP-9 expression levels were reduced by NONO knockdown. Representative blots from at least two independent experiments are shown. β-actin was used as an internal control. **e** The migration of siRNA-scr and siRNA-NONO-transfected MDA-MB-231 cells was studied by staining the cells with Green CMFDA and injecting them into the perivitelline space of 48 hpf wild-type zebrafish embryos, and the images were taken with a fluorescence microscope, as described in the Materials and methods section. The number of migrated cells per zebrafish embryo was counted manually using FIJI software. Representative images from 72 h post-injection are shown. Scale bar, 100 µm and magnification, 10X. The graph represents the mean ± SD from *n* > 25 embryos. Statistical analysis was performed using non-parametric two-tailed Mann-Whitney *U*-test

To demonstrate the effect of NONO silencing on the migratory potential of breast cancer cells. First, the wound healing assay was performed. As in Fig. 7a, b; depletion of NONO in MDA-MB-231 and MCF-7 resulted in reduced recovery of the artificial wound area compared to siRNA-scr-transfected cells during examination time points after 12, 24, and 48 h simultaneously.

Next, with/without Matrigel Transwell inserts were used to study the invasive and migratory potential of siRNA NONO knockdown MDA-MB-231 and MCF-7 cells. It was found that NONO knockdown significantly decreased the ability of breast cancer cells to migrate and invade (Fig. 7c). In addition, Western blot analysis was performed to examine the expression levels of migration-related proteins. NONO silencing was shown to alter protein expression levels of matrix metalloproteinases (MMPs) and epithelial-mesenchymal transitions (EMT)-related molecules in breast cancer cells. The expression of MMP-2 and MMP-9 was inhibited. In contrast, the expression of the epithelial marker protein E-cadherin was increased. The expression of the mesenchymal marker vimentin was reduced after silencing NONO gene (Fig. 7d). Next, MDA-MB-231 cells with known invasion/metastatic abilities were investigated whether high NONO expression would cause MDA-MB-231 breast cancer cells to spread throughout zebrafish using the experimental settings described [40, 41]. Green CMFDA-stained scrambled and NONO-siRNA-transfected cells were injected into the perivitelline space at 48 h post-fertilization (hpf) of zebrafish embryos and examined at 72 h post-injection (hpi). Quantitative analysis of fluorescent tumor cells per zebrafish embryo using a modified version of the Fiji software showed that NONO silencing significantly reduced the number of migrated cells in zebrafish embryos compared to the control group (Fig. 7e). This data is consistent with another independent experiment (Supplementary Fig. S7), thereby supporting the role of NONO in the proliferation and metastasis of breast cancer cells in the zebrafish xenograft model. These results suggest that NONO is a positive regulator in cell migration and invasion and induces the EMT of breast cancer cells.

### NONO promotes the population of cancer stem cells (CSCs) in breast cancer

**Fig. 8:**
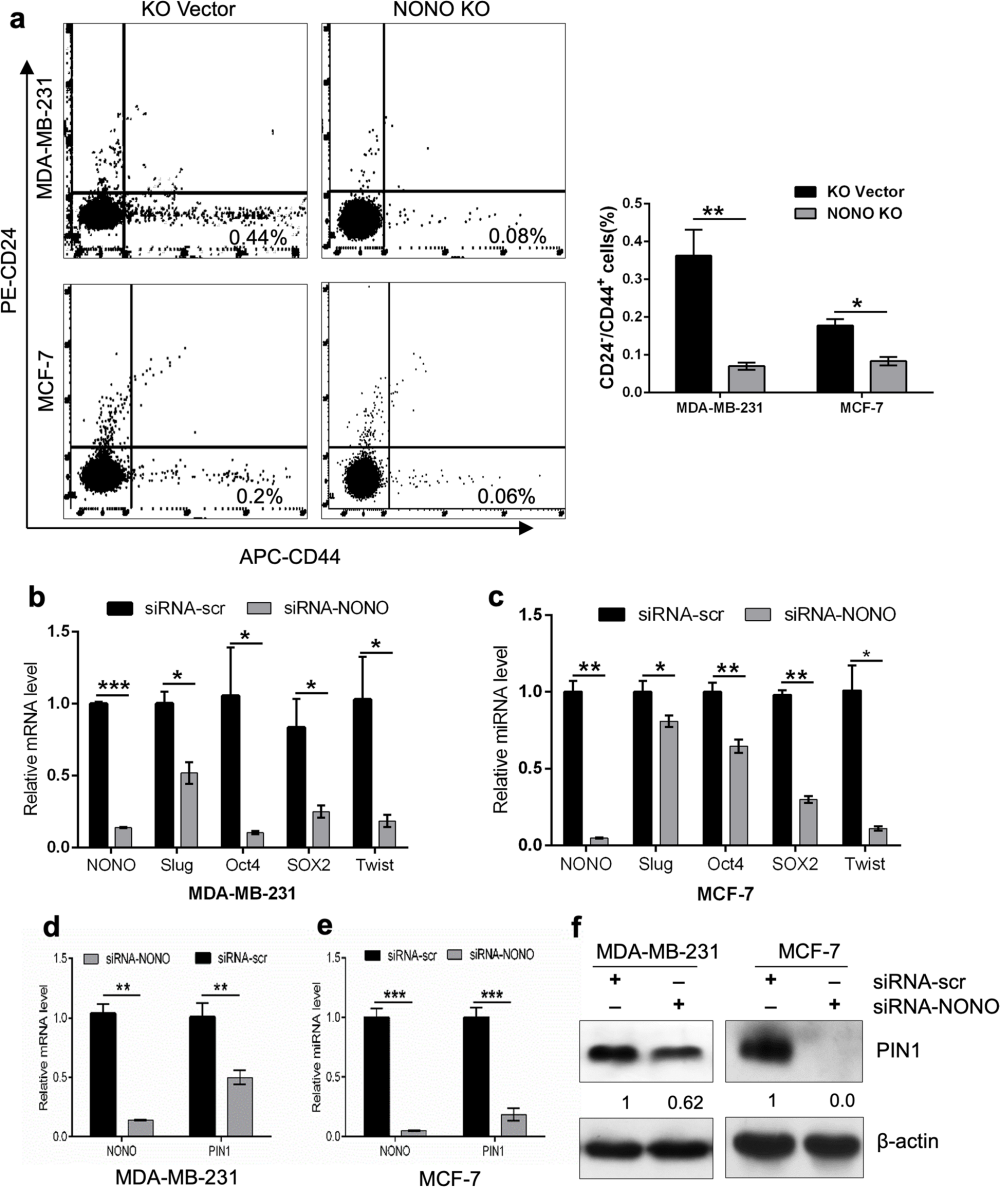
NONO depletion inhibits the cancer stem cell formation in breast cancer cells. **a** FACS measured the subpopulation of cells with CD24^**−**^/CD44^+^ phenotype in MDA-MB-231 and MCF-7 cells. The bar graph shows the quantification of CD24^**−**^/CD44^+^ cells. **b**, **c** qRT-PCR expression analysis of stemness-associated markers, including SLUG, OCT4, SOX2, Twist in MDA-MB-231 and MCF-7 cells. Data presented as mean standard deviation ± SD of three independent experiments. **d**, **e** Quantification NONO- and PIN1 expression in NONO-depleted MDA-MB-231 and MCF-7 cells were analyzed by qPCR. **f** Western blotting analysis of NONO and PIN1 protein in NONO and PIN1-depleted MDA-MB-231 and MCf-7 cells. β-actin was used as a control for equal loading and ImageJ software for densitometry analysis. **a**-**e** Data represented as mean standard deviation ± SD of three independent experiments. ***p < 0.001, p** < 0.01, *p < 0.05

Cancer stem cells have been shown to promote tumor initiation and development, metastasis, relapse, and resistance to treatment. To determine the role of NONO in breast cancer cell stemness, we generated NONO-knockout MDA-MB-231 and MCF-cells using the CRISPR-Cas9 system. Successful knockout of the *NONO* gene in MDA-MB-231 and MCF-7 cell lines was confirmed by Western blotting (Supplementary Fig. S8). Breast cancer cells expressing the phenotypic CD24^**−**^/CD44^+^ markers on their surface have stem cell-like properties, including the ability to self-renew and initiate tumors [42, 43]. Flow cytometric analysis revealed that knockout of the *NONO* gene significantly reduced the population of CD24^**−**^/CD44^+^ cells in MDA-MB-231 and MCF-7 cells (Fig. 8a). In addition, NONO silencing reduced mRNA levels of several central pluripotency stem cell regulators (*OCT4, SOX2, Slug,* and *Twist*) in MDA-MB-231 and MCF-7 cells (Fig. 8b, c). Since PIN1 is highly expressed in most cancers, particularly cancer stem cells [40, 41], we investigated the effect of NONO silencing on PIN1 expression. RT-qPCR and Western blot analysis showed that NONO silencing significantly reduced PIN1 mRNA and protein levels suggesting that PIN1, indicating that NONO promotes CSC-like properties in breast cancer cells via regulation of PIN1 expression (Fig. 8d-f).

### Silencing of NONO increases PD-L1 expression at the surface of breast cancer cells

**Fig. 9:**
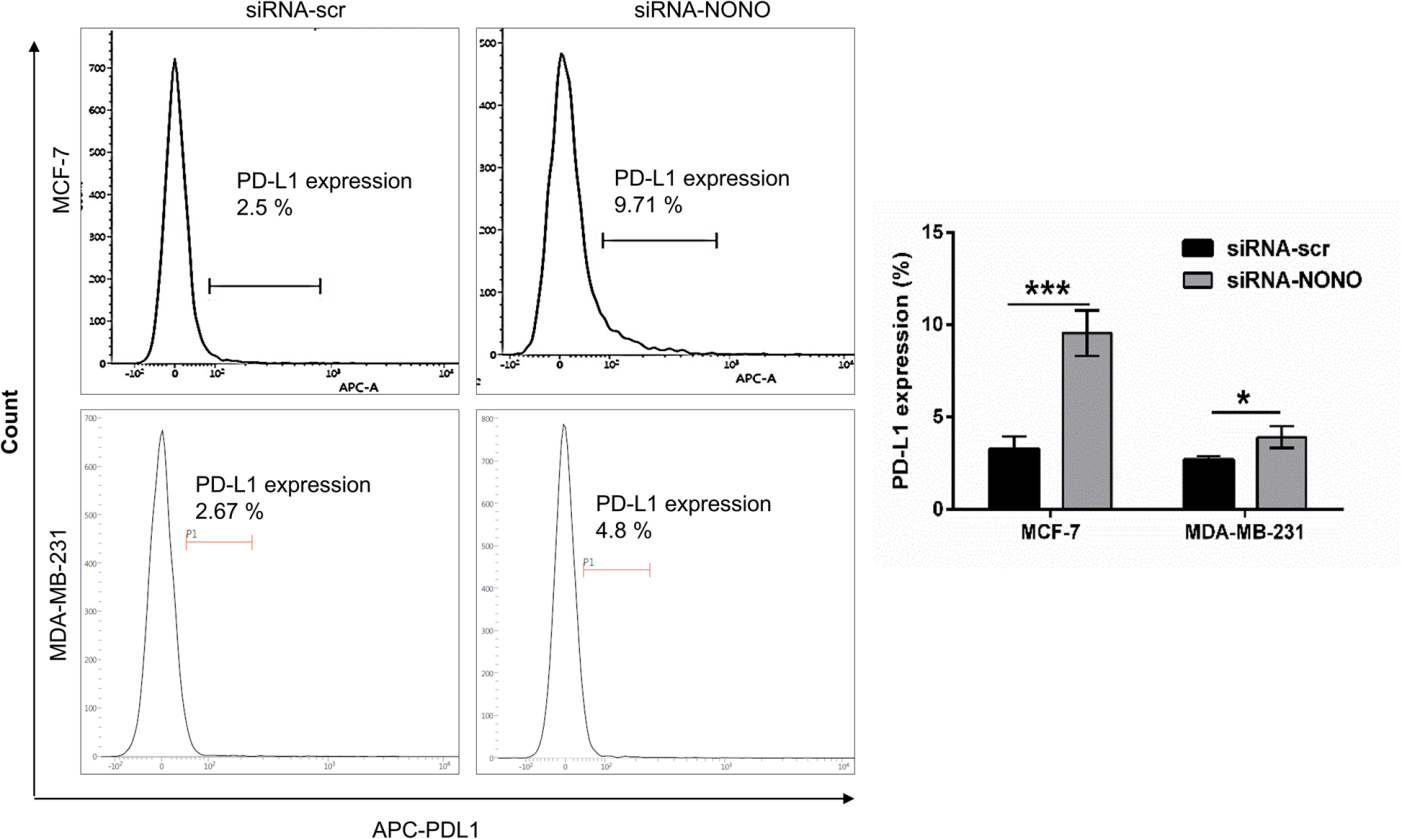
NONO inhibits the expression of PD-L1 in breast cancer cells. NONO Silencing promotes PD-L1 protein expression on the surface of MDA-MB-231 and MCF-7 breast cancer cells. Bar graphs presented as the mean standard deviation ± SD of three independent experiments. ***p < 0.001, *p < 0.05

Programmed cell death protein 1 ligand (PD-L1) is an inhibitory molecule expressed by tumor cells to induce anergy on tumor-responsive cells [44–46]. Increased expression of PD-L1 in cancer cells using multiple approaches (e.g. interferon and radiation therapy) amplifies the response of immune checkpoint block therapy in experimental models [46, 47]. To determine the effect of NONO silencing on PD-L1 expression, flow cytometric analysis was performed using an anti-PD-L1 APC antibody. NONO silencing was shown to significantly increase the PDL-1 expression in MDA-MB-231 and MCF-7 cells (Fig. 9). Therefore, targeting of NONO in breast cancer could bring effectiveness of ICB and could improve the survival of breast cancer patients.

### Silencing of NONO suppresses the activation of the Akt/MAPK/β-catenin signaling pathway

**Fig. 10:**
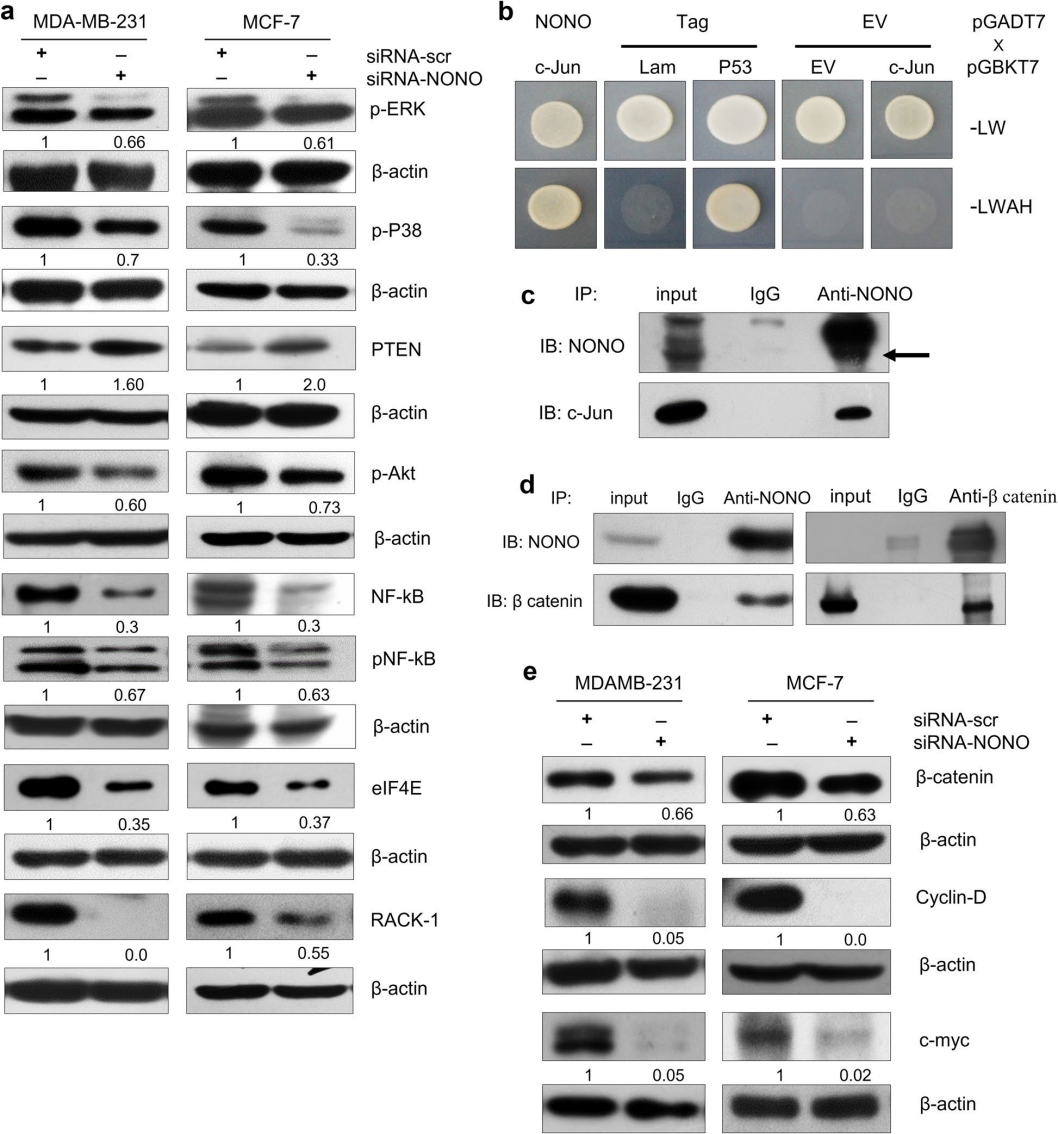
NONO activates MAPK/β-catenin signaling pathway in breast cancer cells. **a** Total cellular protein (50 µg) from siRNA-transfected MDA-MB-231 and MCF-7 cells was analyzed by Western blotting with Akt/MAPK antibodies to the proteins as indicated. **b**, **c** The interaction of NONO and c-Jun was examined by yeast two-hybrid and immunoprecipitation/Western blotting. **d** The immunoprecipitated complex of MDA-MB-231 cell lysates with anti-NONO or anti-β-catenin antibody analyzed by Western blotting (IB). **e** Silencing of NONO in MDA-MB-231 and MCF-7 cells suppresses β-catenin and related downstream molecules such as c-myc and cyclin D1. **a**, **e** Representative blots from two independent experiments are shown. β-actin was used as a control for equal loading and ImageJ software for densitometry analysis

To examine the relationship between NONO and MAPK signaling pathway, a Western blotting assay was performed to measure protein expression of PTEN, p-Akt, p-p38, p-Erk, eIF4E, NF-kB, p-NF-kB, and RACK-1. It was shown NONO silencing promotes the PTEN protein expression while reducing expression levels of p-Akt, p-Erk, p-P38, NF-kB, p-NF-kB, eIF4E, and RACK-1 compared to siRNA-scr-transfected in both in MDA-MB-231 and MCF-7 cells (Fig. 10a). In addition, we characterized the interaction of NONO with c-Jun using a yeast two-hybrid system (Fig. 10b). The interaction was further validated by NONO immunoprecipitation/Western blot analysis using MCF-7 lysate (Fig. 10c). In addition, a physical interaction between endogenous NONO and β-catenin proteins was also observed in MCF-7 cells using IP/Western blotting analysis (Fig. 10d), suggesting that NONO complexed with β-catenin may be involved in tumor progression of breast cancer cells by affecting the β- catenin signaling. Since β-catenin signaling is considered to be an important regulator of cancer stem cell (CSC) self-renewal, we hypothesized that NONO silencing might inactivate β-catenin signaling and thus inhibit the stem cell-like properties and tumorigenicity in breast cancer cells. To test this hypothesis, we investigated that NONO silencing inhibits β-catenin expression. Consequently, the expression of known downstream targets of β-catenin, such as c-myc and CCND1 was downregulated in NONO-silenced MDA-MB-231 and MCF-7 cells (Fig. 10e). Taken together, these results suggest that NONO promotes tumorigenicity of breast cancer cells via the activation of the Akt/MAPK/β-catenin signaling pathway.

## Discussion

Breast cancer is a prevalent cancer in the women population that is associated with high mortality rates and significant socioeconomic burdens [1]. Therefore, a deeper understanding of the molecular mechanisms underlying breast cancer progression is critical for the development of effective clinical therapies and improving patient outcomes. In this study, we identified PIN1 as a positive regulator of NONO and characterized the downstream targets of NONO that drive the breast cancer progression. We found that binding of PIN1 with the c-terminal thr-pro motifs of NONO leads to increased stability and abundance of NONO, which in turn promotes tumorigenesis by activating cancer-promoting genes and inactivating tumor suppressors.

In our bioinformatic analysis of TCGA data and immunohistochemical analysis of breast cancer tissues, we examined the higher expression of NONO in breast cancer tissues. We further elucidated the important role of NONO in cell viability, cell proliferation, cell cycle, migration and invasion, apoptosis, and stemness of breast cancer cells in vitro. The inhibitory effect of NONO knockdown on the migration potential of breast cancer cells was further demonstrated in in vivo zebrafish xenograft model. In addition, our study revealed the novel interactions of NONO with c-Jun and β-catenin and demonstrated the role of NONO in regulating Akt/MAPK/β-catenin pathways. These findings highlight the importance of NONO in breast cancer development and progression and demonstrated its potential as a therapeutic target for the disease.

There has been a recent realization that NONO is involved in every multiple steps of gene regulation [8–10]. It is thus important to understand how this versatile protein is regulated. Yeast-two hybrid assay demonstrated that NONO directly interacts with PIN1, and this interaction is mediated through the WW domain of PIN1. The interaction of NONO with the WW domain of PIN1 is further supported by our FRET analysis. PIN1 is known to bind phospho serine/threonine-proline (pS/T-P) motifs in other protein targets, and NONO protein has four T-P motifs. Therefore, we generated mutant NONO with T-to-A substitutions to investigate T-P motif(s) important for binding with PIN1-WW domain. In our yeast-two hybrid colony formation assay the point mutations in NONO protein, namely T428A and T450A substitutions, render NONO unable to produce interacting colonies with WW-domain of PIN1. Taken together, our study demonstrated that WW-domain of PIN1 specifically binds with two T-P motifs at the C-terminal region of NONO. We further identified that PIN1 is critical for the stabilization of NONO protein. In our experiment we observed a decrease in NONO protein levels following treatment with PIN1 inhibitor juglone and this effect was rescued by using the proteasomal inhibitor MG132, indicating that the interaction of PIN1 with NONO blocks the ubiquitination of NONO protein and promotes stability.

Previous studies have shown that NONO is strongly expressed in multiple cancer types. In addition, NONO has been reported to regulate the growth of MCF-7 cells that depends on the interaction of NONO with sterol regulatory element binding protein 1 (SREBP1) [12]. However, the upstream modulatory role of NONO in the expression of a network of molecules that are associated with various cancer cell processes has not been characterized. In this study, the knockdown of NONO showed anti-proliferation function and S-phase arrest in breast cancer cell lines. Disruption in the cell cycle control mechanism is a hallmark of many common malignancies, leading to unchecked cell division [48]. We then hypothesized whether silencing of NONO has any effect on the expression of cell cycle-related genes including cyclin D, p21, and PCNA. Studies have shown that cyclin D1 is highly expressed in the mid-to-late G1 phase and exhibits an up-regulated kinase activity when bound to cdk4 in cancer cells [49]. The maximal level of cyclin D during the G1 phase is required for the cell to initiate DNA synthesis but must be repressed at a low level during the S-phase for effective DNA synthesis [50]. Our results revealed that silencing of NONO resulted in the reduction of cyclin D1 levels in MDA-MB-231 and MCF-7 cells which indicates a transition of cells from G1 to S-phase of the cell cycle. Studies have further demonstrated that inhibiting cyclin D upregulates p21 expression, thereby inhibiting cancer cell proliferation and growth. Our observation that silencing of NONO induced S-phase arrest and p21 expression which is consistent with a previous report that overexpression of p21 gene resulted in S-phase arrest [51]. p21 can also inhibit DNA replication by directly binding to proliferating cell nuclear antigen (PCNA) [52]. We observed a reduction in the levels of PCNA protein upon the silencing of NONO in breast cancer cells, indicating a disruption in DNA replication and supporting an idea that p21 may be involved in mediating this effect.

Arresting cells in S-phase of the cell cycle provides an opportunity for cells to undergo DNA repair or initiate apoptosis. It has been reported that silencing of NONO induces apoptosis in Esophageal squamous cell carcinoma (ESCC) [20]. However, its role in apoptosis in breast cancer cells is not well understood. Our flow-cytometry data indicated a significant stimulation of apoptosis in breast cancer cells with the knockdown of NONO. Moreover, we examined the downregulation of Bcl-2 with the silencing of NONO in breast cancer cells. Given many reports, Bcl-2 binds and inhibits the activities of Bax, thereby preventing mitochondrial membrane permeabilization and apoptosis [53]. In our present study, we found that silencing of NONO induced the loss of mitochondrial membrane potential (ΔΨm) and increased the Bax/Bcl2 ratio that correlates with the sensitivity of cancer cells to chemotherapy drugs [54]. The growth suppressor p53 is a tightly controlled transcription factor that depending on its expression levels, can cause cell cycle arrest or apoptosis [55]. We analyzed the expression level of p53, a potent inducer of p21, in MCF-7 and found that silencing of NONO promotes the upregulation of p53 and p-p53 proteins. p53 is functional in MCF-7 cells but mutated in MDA-MB-231. Based on this finding, we can suggest that silencing of NONO induces S-phase arrest independent of p53, and the arrest might be mainly due to p21 overexpression. Therefore, it can be speculated that in breast cancers with functional p53, targeting NONO can be a novel strategy for promoting p53- mediated cell cycle arrest and apoptosis.

Further, we studied the effect of silencing of NONO on the migration and invasion potential of breast cancer cells. The current study demonstrated that NONO silencing inhibits the migration and invasion capacities of breast cancer cells in vitro*,* and in vivo zebrafish embryo model. The inhibition in migration potential was further proved by a reduction in the levels of MMP-2 and MMP-9 proteins. MMP-2 and MMP-9 are the major enzymes involved in the degradation of extracellular matrix (ECM) and plays crucial role in the metastatic dissemination of cancer cells [56]. Epithelial-mesenchymal transition (EMT) is believed to be a crucial process in cell metastasis. Studies have shown that high vimentin expression with loss of E-cadherin is associated with the EMT of various cancers, including breast cancer [57]. Our results indicated that silencing of NONO reversed the high expression of vimentin and low expression of E-cadherin in breast cancer cells, thereby inhibiting the metastasis and EMT of breast cancer cells.

EMT has been linked to the increased stemness of tumors and displays the cancer stem cell (CSC) phenotype [58]. CSCs are a small number of self-renewing cells within the tumors that contributes to tumor heterogeneity, therapy resistance, and distant metastasis [42, 59]. The CD24^−^/CD44^+^ phenotype in the cell population is commonly used to characterize CSCs in breast cancer [42]. Our results showed that knockout of NONO in breast cancer cells significantly reduced the number of CD24^−^/CD44^+^ expressing cells. Also, the mRNA profile of stemness markers SLUG, OCT4, SOX2, and Twist were significantly reduced in NONO knockdown cells. All these results depicted that NONO is a positive regulator of CSCs in breast cancer. In breast cancer, the increased levels of PIN1 have been linked to the acquisition of stem cell-like traits [43]. Our results have indicated that silencing of NONO inhibits the PIN1 expression thereby inhibiting the CSC formation in breast cancer.

In addition, the association of NONO with Akt/MAPK/β-catenin pathways was investigated in this study. Akt, MAPK and β-catenin pathways have been reported to be involved in breast cancer cell survival, proliferation, migration and invasion and, stemness [60, 61]. This study showed that silencing of NONO inhibited the levels of p-Erk, p-Akt, and p-P38, thereby inhibiting the signaling cascade involved in various cellular processes. The PTEN/Akt signaling pathway is frequently disrupted in cancer. Loss of PTEN leads to the constitutive activation of downstream signaling pathways, including Akt, and fuel the cancer progression [62]. Interestingly, an increase in PTEN protein expression was observed in NONO knockdown breast cancer cells, indicating that NONO regulates PTEN levels and Akt signaling. Akt signaling is well known as a major upstream element of the NF-kB signaling pathway. We then hypothesized that NF-kB could be regulated by NONO. At present study, it revealed that NONO knockdown exhibited a significant decrease in the protein expression of NF-kB and p-NF-kB, indicating that NONO contributes to cancer cell growth through the activation of the Akt/NF-kB signaling pathway in breast cancer cells. eIF4E (eukaryotic translation initiation factor 4E) and RACK1 (Receptor for Activated C kinase 1) are proteins involved in protein translation [63], and their interaction with Akt is functionally linked in the regulation of cellular processes, including protein synthesis and signal transduction. Our results demonstrated that silencing of NONO reduced the protein levels of eIF4E and RACK1, suggesting that NONO may have a role in regulating the protein translation process in cancer cells. The depletion of NONO and the subsequent reduction in β-catenin protein expression, as well as the downregulation of its downstream targets (c-myc, cyclinD1, and CD44) suggest the role of NONO in modulating the oncogenic properties associated with the β-catenin signaling. In addition, the current study revealed NONO as an interacting partner of β-catenin and c-Jun providing evidence for the direct involvement of NONO in the regulation of β-catenin and MAPK pathways.

Cancer immunotherapy has been recently developed to enhance the ability of an immune system to effectively target and eliminate cancer cells [64]. Targeting the PD-1/PD-L1 axis has emerged as an effective strategy in cancer immunotherapy. Immune checkpoint inhibitors that block this interaction can restore T cell activity and enhance anti-tumor immune response. However, the success of immune checkpoint inhibition is limited in cancer with lower levels of PD-L1. Our findings indicated that silencing of NONO promotes the upregulation of PD-L1 expression levels. By increasing the expression of PD-L1 on cancer cells, NONO silencing could potentially overcome the limitations of immune checkpoint inhibition in cancers with lower PD-L1 levels. This finding is consistent with the recent finding that inhibition of PIN1 elevated the expression of PD-L1 in human cancers and potentiates immune checkpoint blockade [47]. Further research is needed to elucidate the underlying mechanism by which NONO regulates PD-L1 expression and to evaluate the potential synergistic effect of combining NONO silencing with immune checkpoint inhibitors.

In conclusion, this study provides insights that NONO functions as an oncogene in breast cancer, contributing to tumorigenicity by regulating the expression/activation of genes involved in key cellular processes such as cell proliferation, cell survival, migration and invasion, EMT and stem cell formation. Furthermore, NONO is implicated in activating Akt/MAPK/β-catenin signaling pathways which are well known to play critical roles in cancer progression. Thus, targeting NONO in breast cancer could be a potential therapeutic approach, and could show better antitumor efficiency in combination with immunotherapy.

## Supplementary Information

**Additional file 1: Supplementary Fig. S1.** Validation of antibody specificity by western blotting and immunocytochemistry. A, B Watern blot analysis of NONO and PIN1 silenced cell lysate, the knockdown is shown along with control and scrambled-siRNA transfected cells. β-actin was used as internal control. C, D Immunocytochemistry analysis of NONO and PIN1 knockdown cells along with siRNA-scr transfected cells. Cells were fixed, permeabilized and incubated with mouse monoclonal anti-NONO and anti-PIN1 antibodies, followed by goat anti-mouse IgG FITC secondary antibody incubation and counter-stained with DAPI. **Supplementary Fig. S2.** Data sets retrieved from CPTAC demonstrated a significant increase in the levels of NONO proteins in breast cancer tissues as well as in different stages and subclasses as compared to normal breast tissues. **Supplementary Fig. S3.** Acceptor photobleaching FRET analysis revealed WW-domain dependent interaction of PIN1 with NONO. Flourescence intensities of CFP and YFP of HEK293T cells co-transfected with NONO-YFP/PIN1-CFP,NONO-YFP/PIN1-WW-CFP, and NONO-YFP-PPIase-CFP plasmids were measured after acceptor photobleaching. The intensities of CFP are elevated in FRET pair NONO-YFP/PIN1-CFP, and NONO-YFP/ PIN1-WW-CFP while no increase in the intensity of CFP was observed in NONO-YFP/PIN1-PPIase pair. **Supplementary Fig. S4.** Rescue of knockdown phenotype. A Restriction digestion confirmation of successful cloning of human NONO cDNA into the pcDNA3.1plasmid. B Verification of silent mutations generated in NONO construct by Sanger sequencing. C Rescue of siRNA knock-down phenotype with the exogenous expression of resistant construct. **Supplementary Fig. S5.** The expression of NONO is elevated in breast cancer cell lines. Western Blotting was used to compare the proteins levels of NONO in breast cancer cell lines with normal human mammary epithelial cells. **Supplementary Fig. S6.** Flow cytometric analysis demonstrated that silencing of NONO induces the ROS generation in MDA-MB-231 breast cancer cells. **Supplementary Fig. S7.** The proliferation and metastasis potential of MDA-MB-231 breast cancer cells in zebrafish xenograft model was inhibited by silencing the NONO gene expression. CMFDA Green-labelled MDA-MB-231 cells transfected with siRNA-scr and siRNA-NONO were injected into the perivitelline space of 48 h post-fertilization embryos, and the proliferation and metastasis of cells were detected under fluorescent microscopy at different time point after injection. **Supplementary Fig. S8.** Immunoblot screening of CRISPR-Cas9 mediated knockout of NONO gene in the colonies of MDA-MB-231 and MCF-7 cells.

**Additional file 2.**

## Data Availability

The datasets used and analyzed during the current study are available from the corresponding authors upon reasonable request.
