# Supplementary material for: Non-POU Domain-Containing Octomer-Binding (NONO) protein expression and stability promotes the tumorigenicity and activation of Akt/MAPK/β-catenin pathways in human breast cancer cells
Source: Cell Commun Signal. 2023 Jun 27;21:157. doi: 10.1186/s12964-023-01179-0 (PMC10294335; doi:10.1186/s12964-023-01179-0)
Supplement: Supplementary file 2 — Additional file 1: Supplementary Fig. S1. Validation of antibody specificity by western blotting and immunocytochemistry. A, B Watern blot analysis of NONO and PIN1 silenced cell lysate, the knockdown is shown along with control and scrambled-siRNA transfected cells. β-actin was used as internal control. C, D Immunocytochemistry analysis of NONO and PIN1 knockdown cells along with siRNA-scr transfected cells. Cells were fixed, permeabilized and incubated with mouse monoclonal anti-NONO and anti-PIN1 antibodies, followed by goat anti-mouse IgG FITC secondary antibody incubation and counter-stained with DAPI. Supplementary Fig. S2. Data sets retrieved from CPTAC demonstrated a significant increase in the levels of NONO proteins in breast cancer tissues as well as in different stages and subclasses as compared to normal breast tissues. Supplementary Fig. S3. Acceptor photobleaching FRET analysis revealed WW-domain dependent interaction of PIN1 with NONO. Flourescence intensities of CFP and YFP of HEK293T cells co-transfected with NONO-YFP/PIN1-CFP,NONO-YFP/PIN1-WW-CFP, and NONO-YFP-PPIase-CFP plasmids were measured after acceptor photobleaching. The intensities of CFP are elevated in FRET pair NONO-YFP/PIN1-CFP, and NONO-YFP/ PIN1-WW-CFP while no increase in the intensity of CFP was observed in NONO-YFP/PIN1-PPIase pair. Supplementary Fig. S4. Rescue of knockdown phenotype. A Restriction digestion confirmation of successful cloning of human NONO cDNA into the pcDNA3.1plasmid. B Verification of silent mutations generated in NONO construct by Sanger sequencing. C Rescue of siRNA knock-down phenotype with the exogenous expression of resistant construct. Supplementary Fig. S5. The expression of NONO is elevated in breast cancer cell lines. Western Blotting was used to compare the proteins levels of NONO in breast cancer cell lines with normal human mammary epithelial cells. Supplementary Fig. S6. Flow cytometric analysis demonstrated that silencing of NONO induces the ROS [file 12964_2023_1179_MOESM1_ESM.docx]

**Non-POU Domain-Containing Octomer-Binding (NONO) protein expression and stability promotes the tumorigenicity and activation of Akt/MAPK/β-catenin pathways in human breast cancer cell**

**Running title:** NONO promotes the tumorigenicity of breast cancer

Bilal Ahmad Lone^1^, Fouzia Siraj^2^, Preeti Nagar^1^, Ira Sharma^2^, Shweta Verma^3,4^, Shibendra Kumar Lal Karna^1^, Faiz Ahmad^1^, Chetana Sachidanandan^3,4^ and Yuba Raj Pokharel^1^*

^1^Cancer Biology Laboratory, Faculty of Life Science and Biotechnology, South Asian University, Akbar Bhawan, Chanakyapuri, New Delhi-110021, India

^2^National Institute of Pathology, Safdarjung Hospital Campus, Room No.610, 6^th^ Floor, Ansari Nagar, New Delhi, 110029, India

^3^CSIR-Institute of Genomics and Integrative Biology (CSIR-IGIB), New Delhi-110025, India

^4^Academy of Scientific and Innovative Research (AcSIR), Gaziabad- 201002, India

*Corresponding Author: Yuba Raj Pokharel, [yrp@sau.ac.in](mailto:yrp@sau.ac.in)


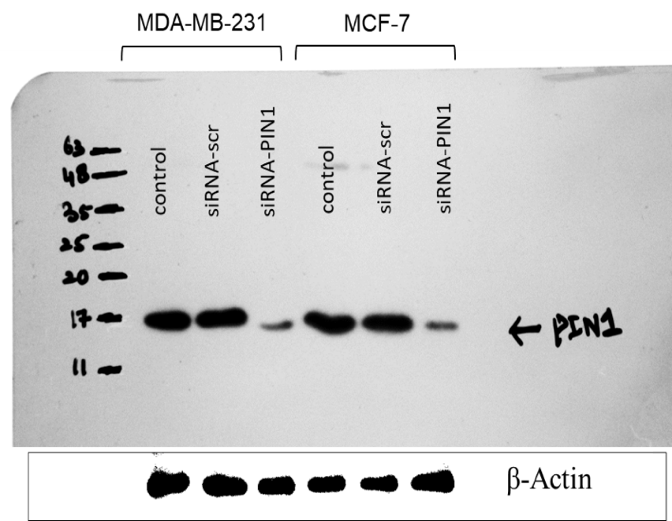

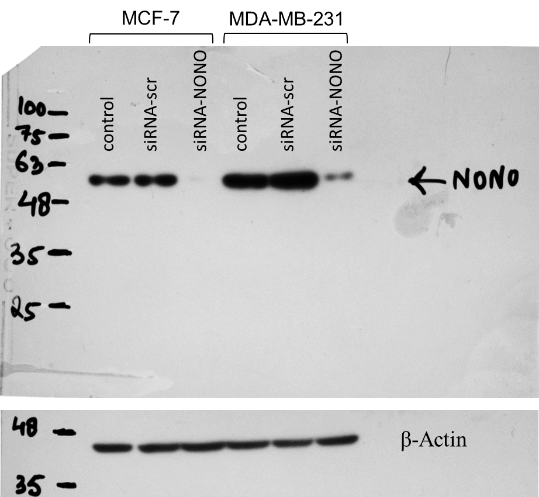


**A**

**B**

**C**

**D**


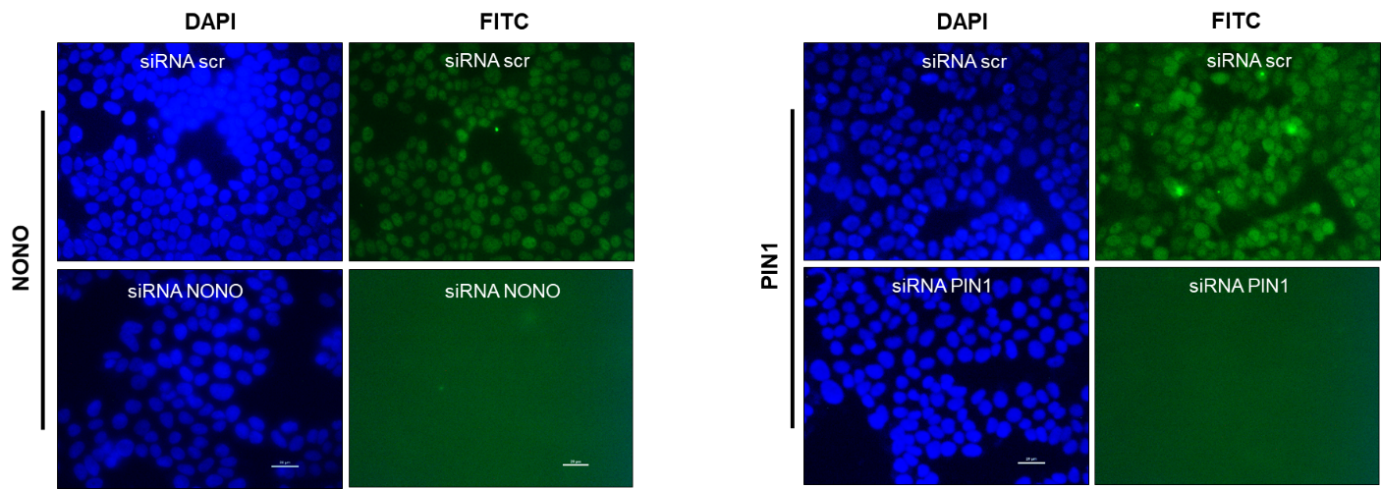


**Supplementary Fig. S1** Validation of antibody specificity by western blotting and immunocytochemistry. **A, B** Watern blot analysis of NONO and PIN1 silenced cell lysate, the knockdown is shown along with control and scrambled-siRNA transfected cells. β-actin was used as internal control. **C, D** Immunocytochemistry analysis of NONO and PIN1 knockdown cells along with siRNA-scr transfected cells. cells were fixed, permeabilized and incubated with mouse monoclonal anti-NONO and anti-PIN1 antibodies, followed by goat anti-mouse IgG FITC secondary antibody incubation and counter-stained with DAPI.


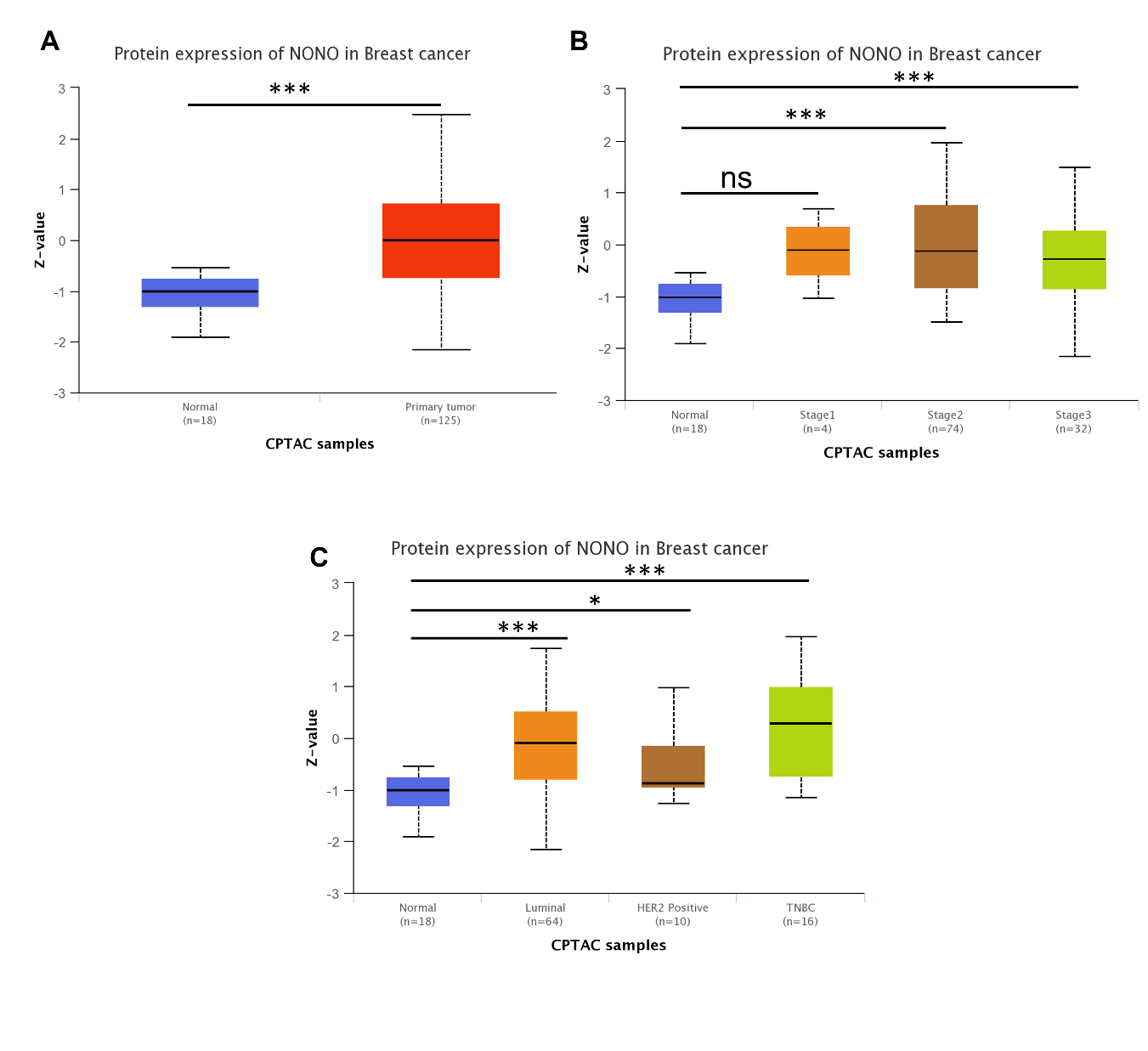


**Supplementary Fig. S2.** Data sets retrieved from CPTAC demonstrated a significant increase in the levels of NONO proteins in breast cancer tissues as well as in different stages and subclasses as compared to normal breast tissues.









**A**

**B**

**C**

NONO-YFP/CFP-PIN1

NONO-YFP/PIN1-WW-CFP

NONO-YFP/PIN1-PPIase-CFP

**Supplementary Fig.** **S3**. Acceptor photobleaching FRET analysis revealed WW-domain dependent interaction of PIN1 with NONO. Flourescence intensities of CFP and YFP of HEK293T cells co-transfected with (A) NONO-YFP/PIN1-CFP, (B) NONO-YFP/PIN1-WW-CFP, and (C) NONO-YFP-PPIase-CFP plasmids were measured afted acceptor photobleaching. The intensities of CFP are elevated in FRET pair NONO-YFP/PIN1-CFP, and NONO-YFP/ PIN1-WW-CFP while no increase in the intensity of CFP was observed in NONO-YFP/PIN1-PPIase pair


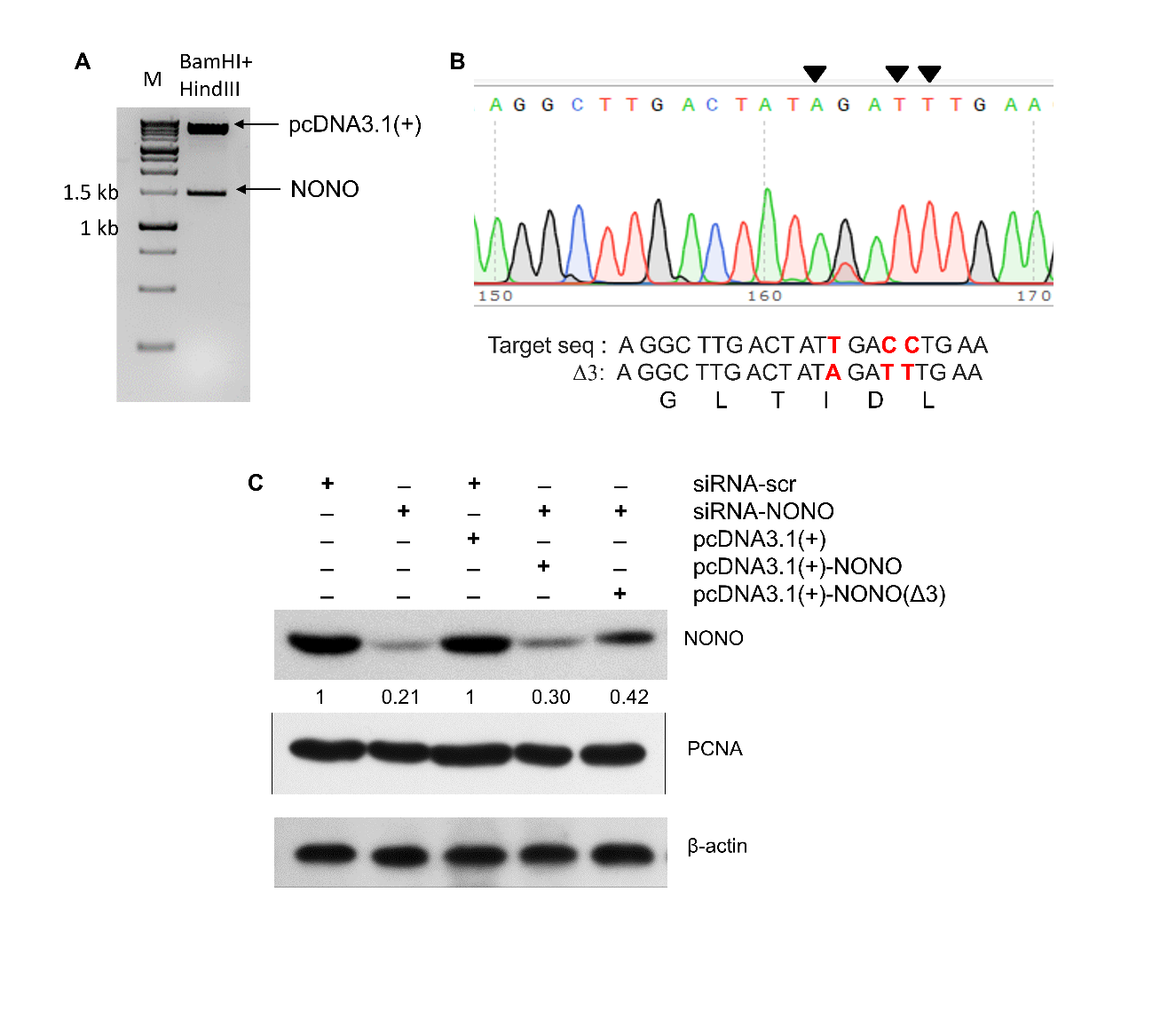


**Supplementary Fig. S4.** Rescue of knockdown phenotype. **A** Restriction digestion confirmation of successful cloning of human NONO cDNA into the pcDNA3.1(+) plasmid. **B** Verification of silent mutations generated in NONO construct by Sanger sequencing. **C** Rescue of siRNA knock-down phenotype with the exogenous expression of resistant construct.


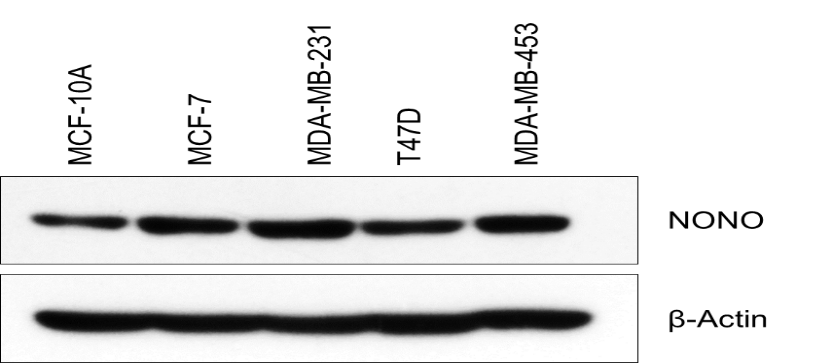


**Supplementary Fig. S5. The expression of NONO is elevated in breast cancer cell lines.** Western Blotting was used to compare the proteins levels of NONO in breast cancer cell lines with normal human mammary epithelial cells (MCF10A)


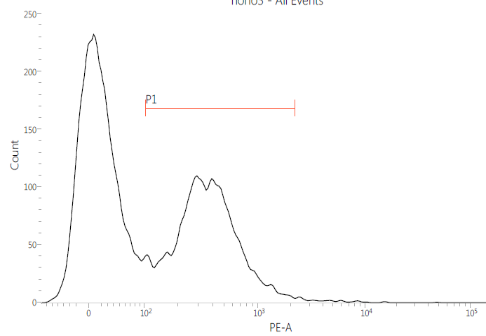

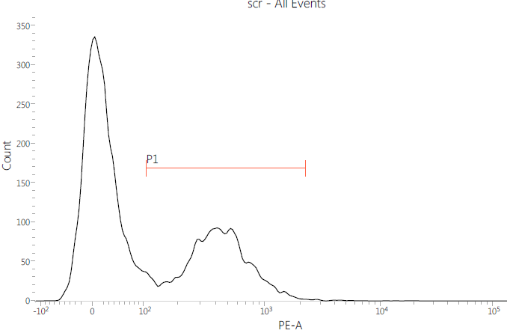




siRNA-NONO

siRNA-scr

36.42 %

44.04 %

ß

**Supplementary Fig. S6.** Flow cytometric analysis demonstrated that silencing of NONO induces the ROS generation in MDA-MB-231 breast cancer cells.


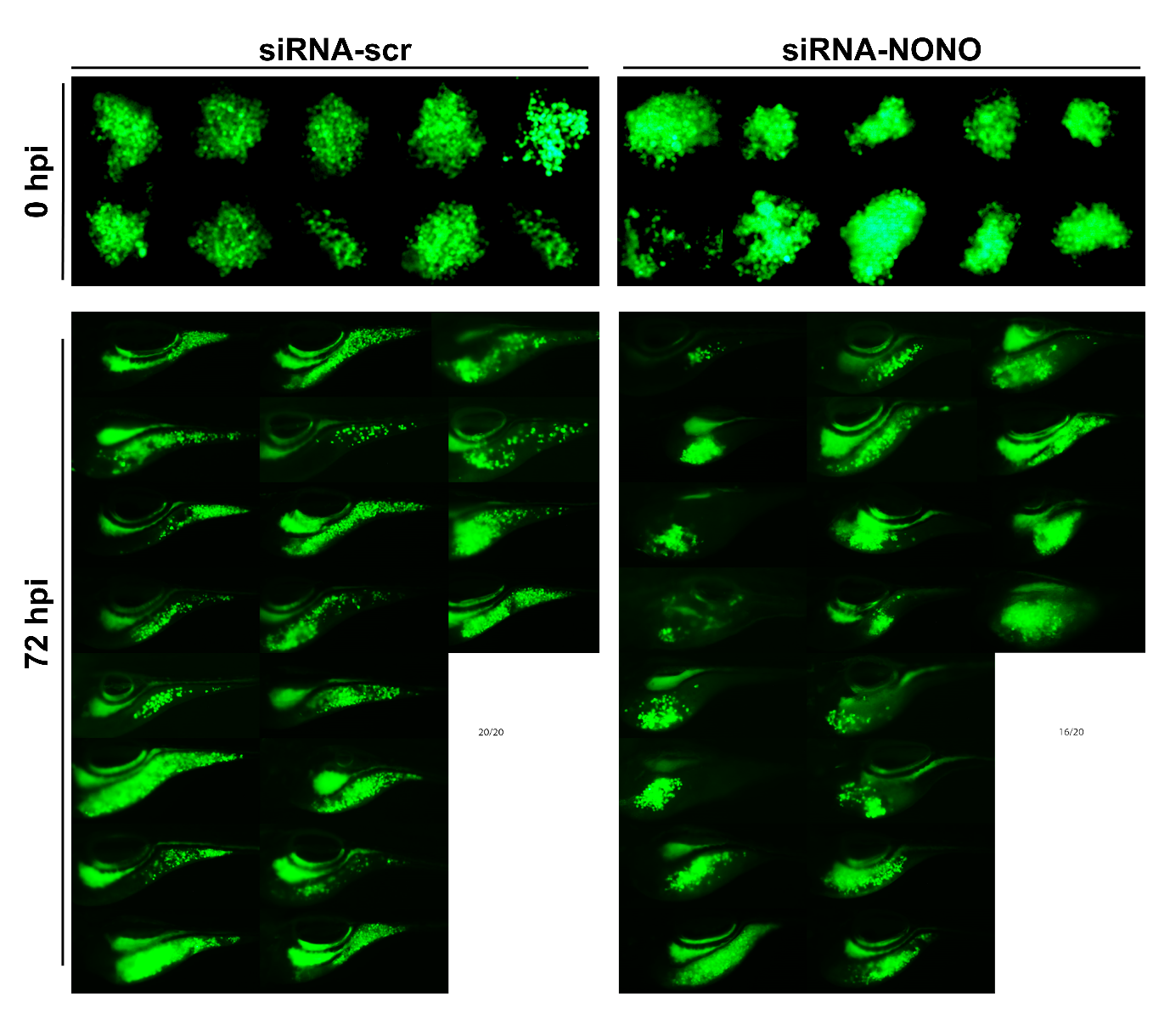


**Supplementary Fig. S7** The proliferation and metastasis potential of MDA-MB-231 breast cancer cells in zebrafish xenograft model was inhibited by silencing the NONO gene expression. CMFDA Green-labelled MDA-MB-231 cells transfected with siRNA-scr and siRNA-NONO were injected into the perivitelline space of 48 h post-fertilization embryos, and the proliferation and metastasis of cells were detected under fluorescent microscopy at different time point after injection.


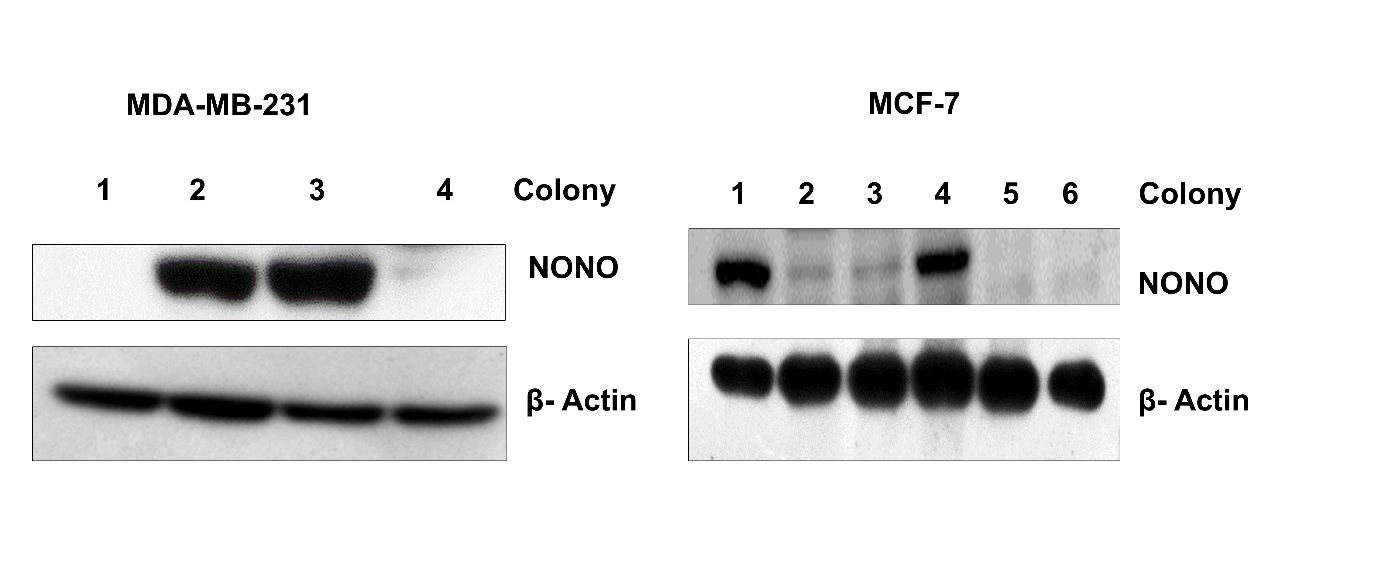


**Supplementary Fig. S8.** Immunoblot screening of CRISPR-Cas9 mediated knockout of NONO gene in the colonies of MDA-MB-231 and MCF-7 cells.
