# Supplementary material for: Non-POU Domain-Containing Octomer-Binding (NONO) protein expression and stability promotes the tumorigenicity and activation of Akt/MAPK/β-catenin pathways in human breast cancer cells
Source: Cell Commun Signal. 2023 Jun 27;21:157. doi: 10.1186/s12964-023-01179-0 (PMC10294335; doi:10.1186/s12964-023-01179-0)
Supplement: Supplementary file 3 — Additional file 2. [file 12964_2023_1179_MOESM2_ESM.pdf]

Fig. 3 A

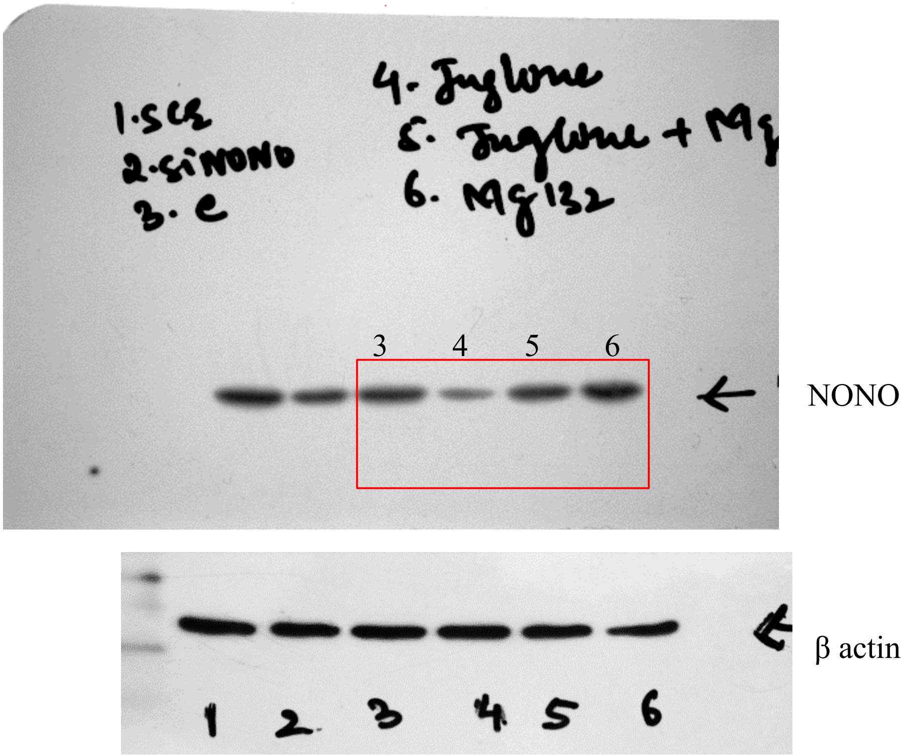
1. Scr
2. siNONO
3. C
4. Juglone
5. Juglone + Mg
6. Mg132
3
4
5
6
NONO
β actin
1 2 3 4 5 6

Fig. 4 b

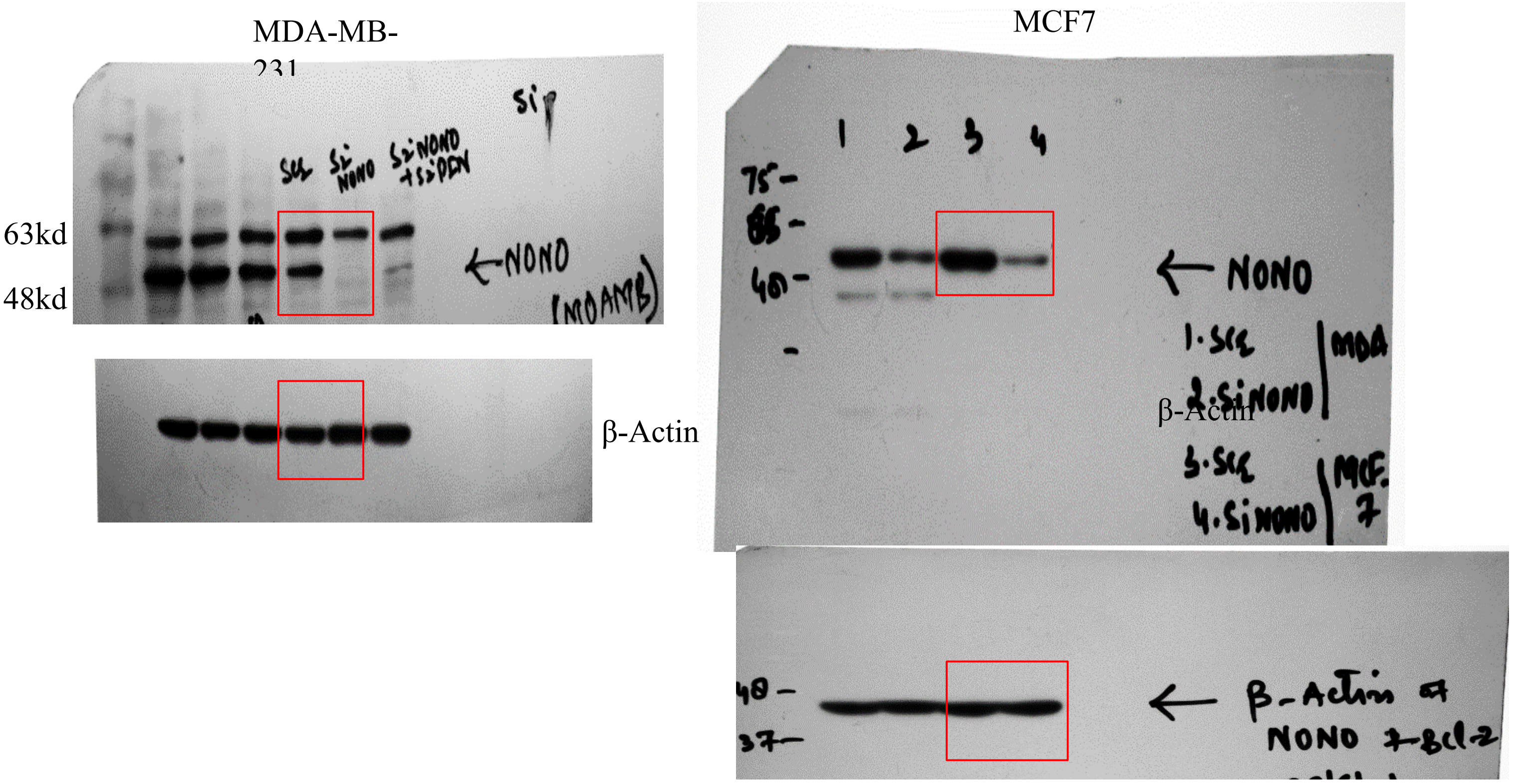
MDA-MB-231
63kd
48kd
Scr
Si NONO
SiNONO +SiPEN
←NONO
(MDAMB)
β-Actin
MCF7
1 2 3 4
75
40
← NONO
1. Scr
2. SiNONO
MDA
3. Scr
4. SiNONO
MCF 7
β-Actin
40
37
β-Actin of NONO + Bcl-2

Fig. 5C

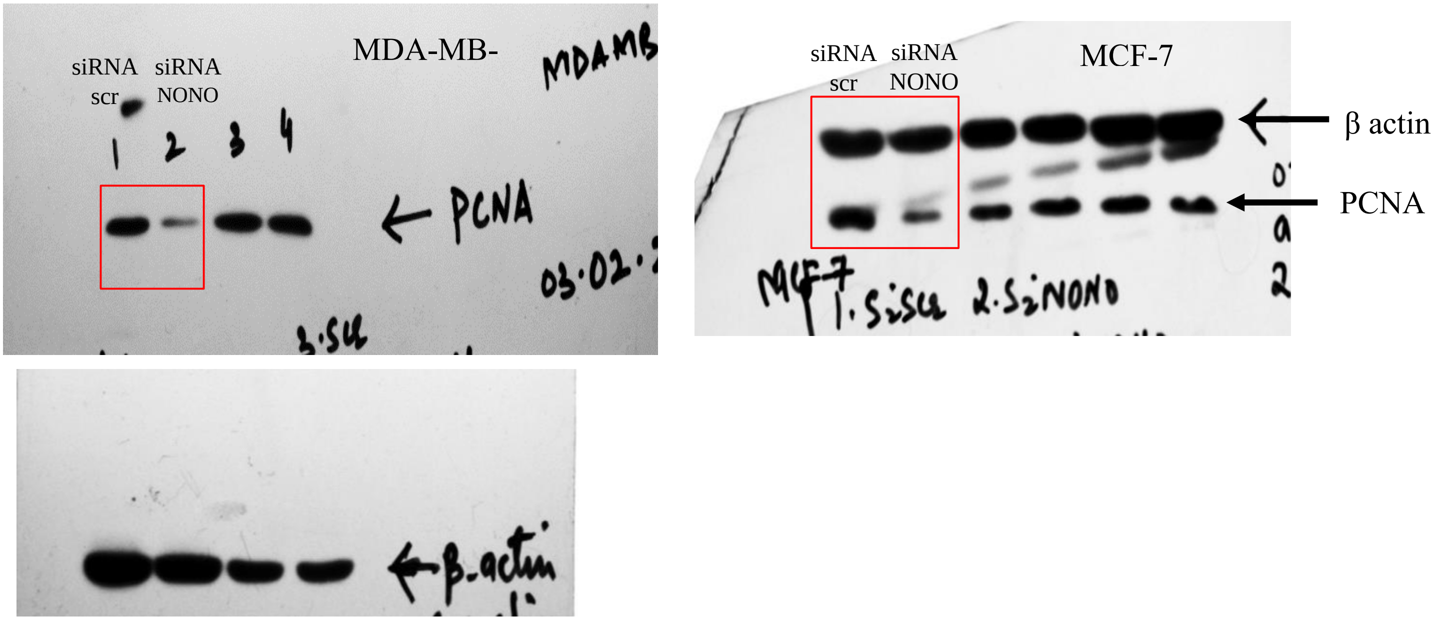
MDA-MB-
siRNA scr
siRNA NONO
MCF-7
siRNA scr
siRNA NONO
β actin
PCNA

Fig. 6

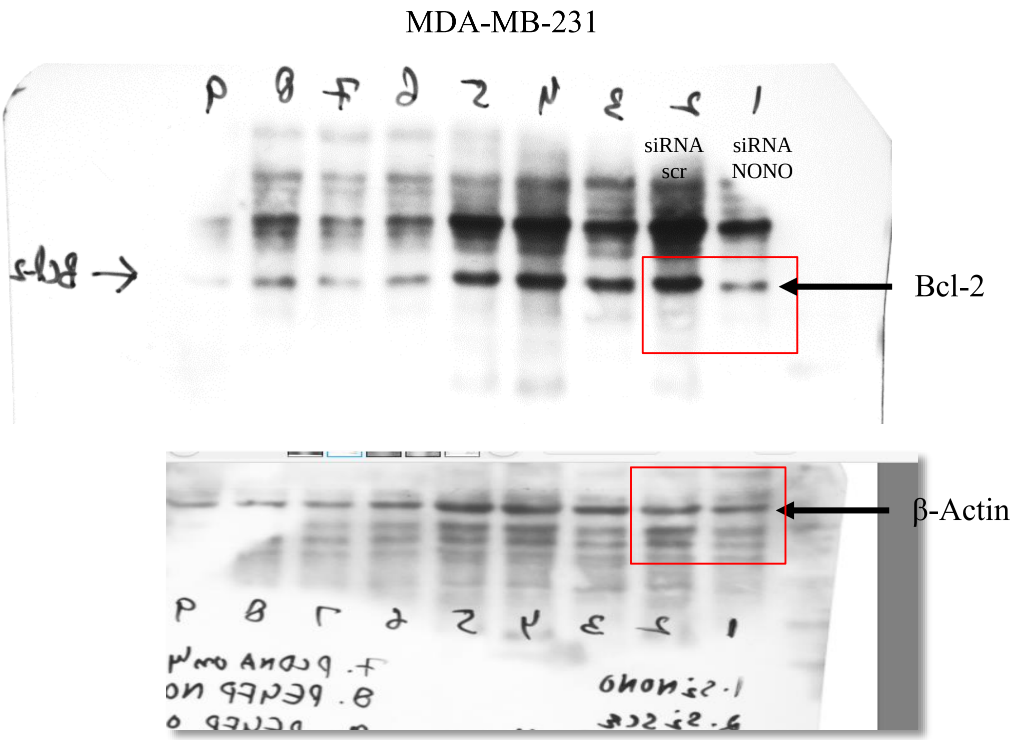
MDA-MB-231
siRNA scr
siRNA NONO
Bcl-2
β-Actin

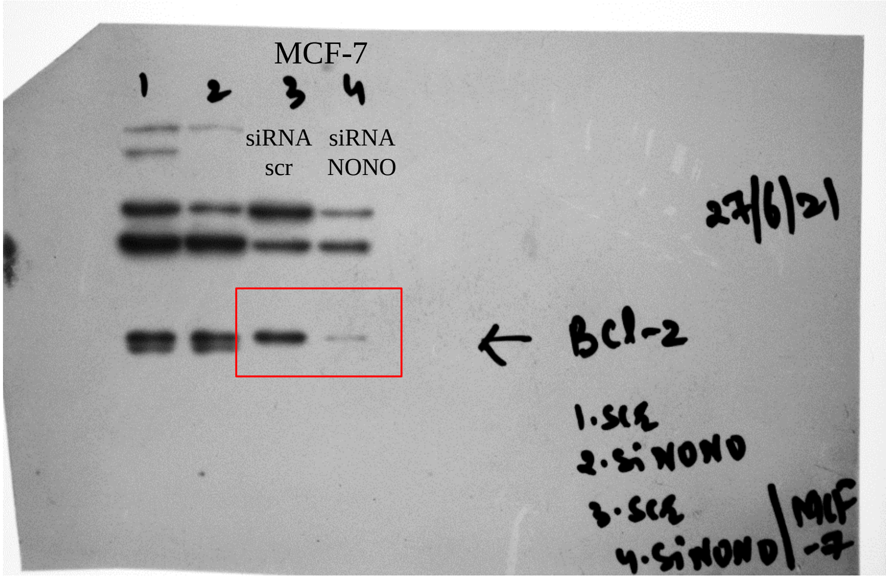
MCF-7
1 2 3 4
siRNA scr
siRNA NONO
27/6/21
Bcl-2
1. SCR
2. Si NONO
3. SCR
4. Si NONO
MCF-7

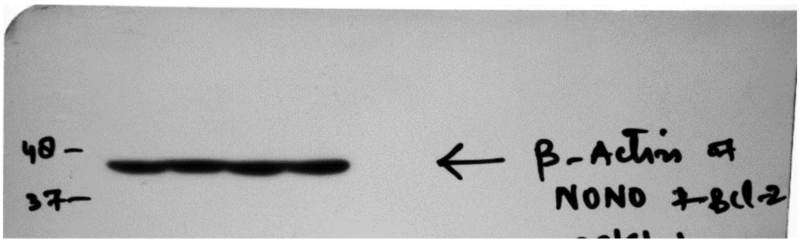
40 –
37 –
β-Actin of
NONO & Bcl-2

Fig. 6 B, C

MDA-MB-231

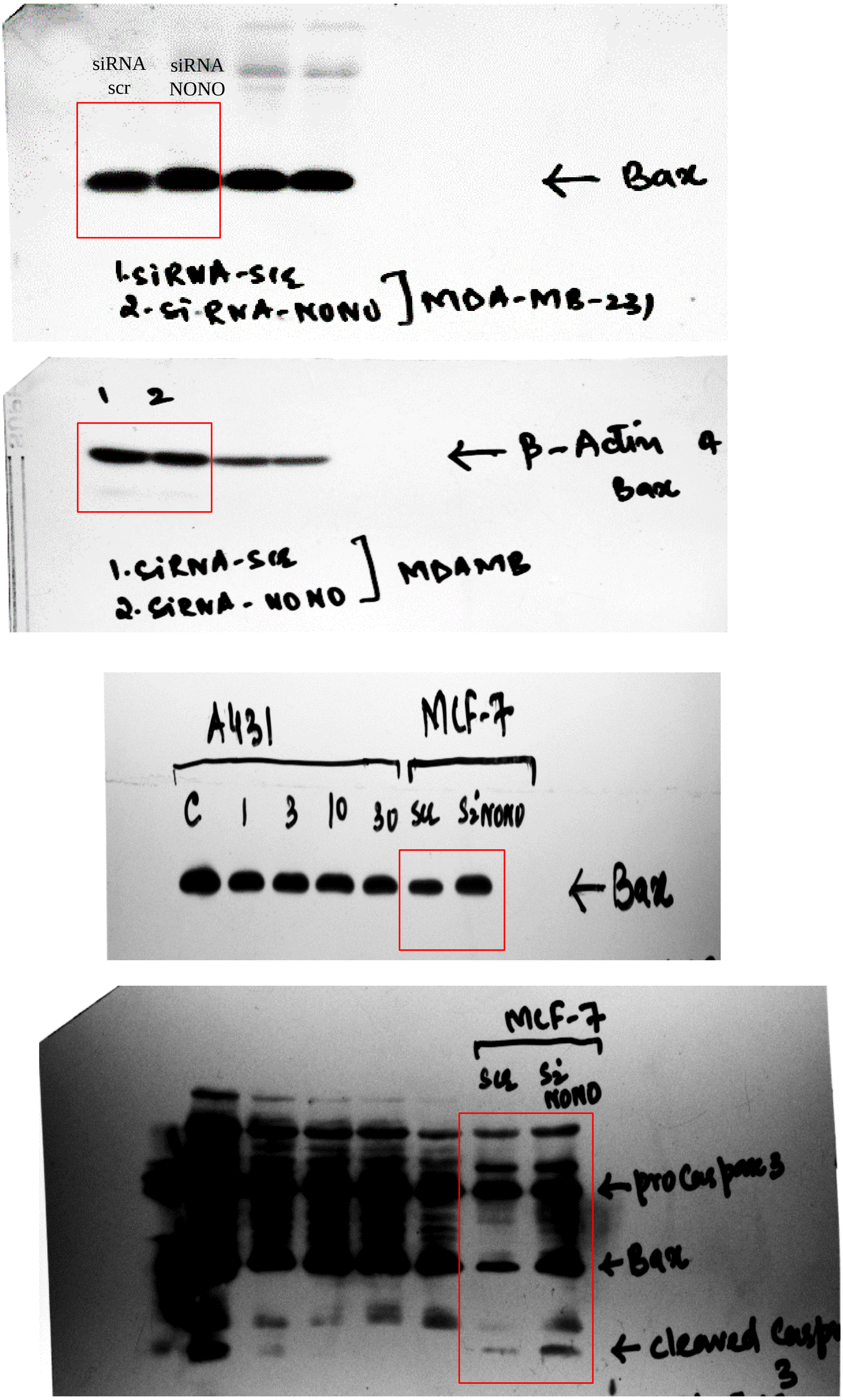

Fig. 6 D

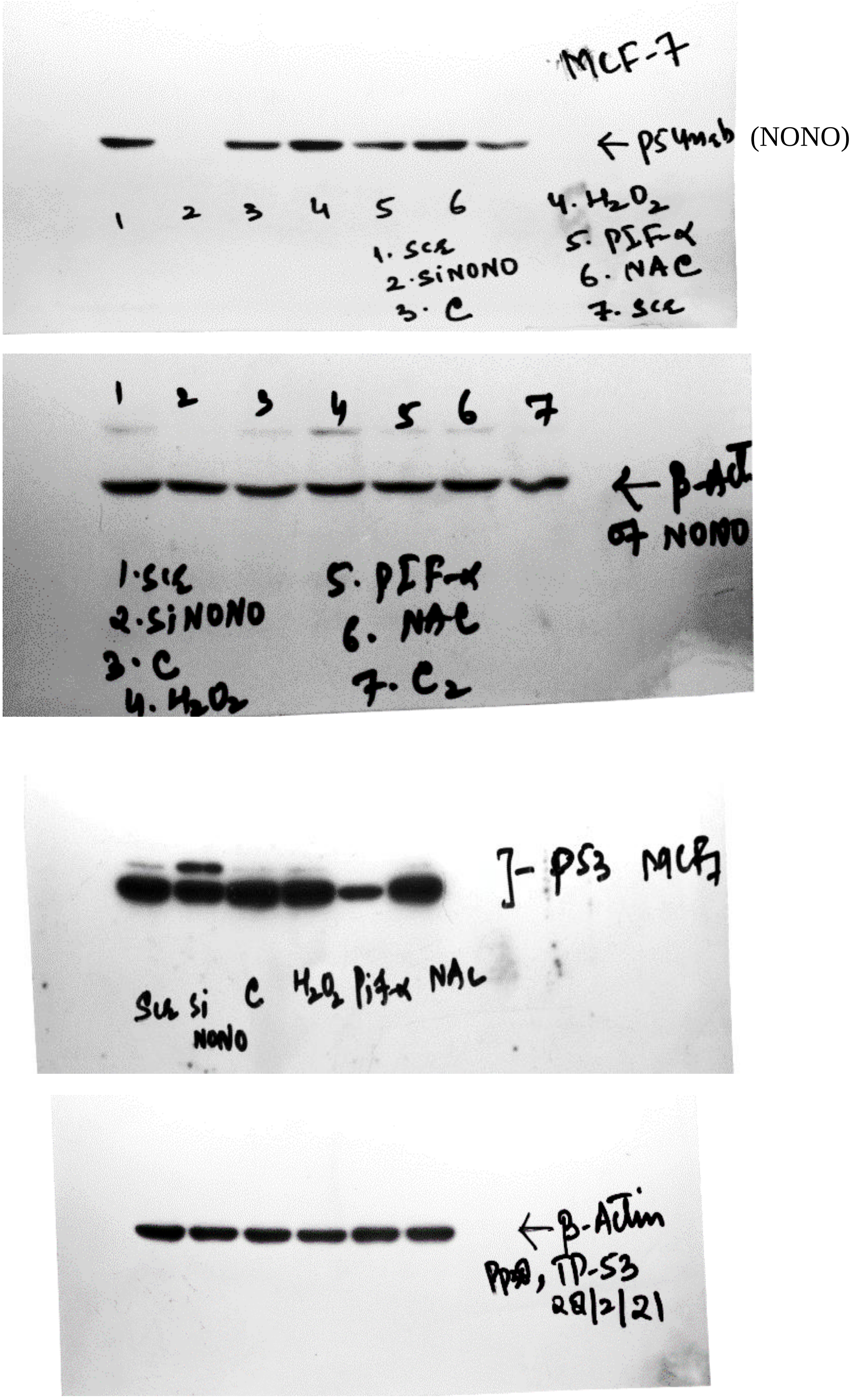
MCF-7
← p54nrb (NONO)
1 2 3 4 5 6
1. scr
2. siNONO
3. C
4. H2O2
5. PIF-α
6. NAC
7. scr
1 2 3 4 5 6 7
← β-Act
of NONO
1. scr
2. siNONO
3. C
4. H2O2
5. PIF-α
6. NAC
7. C2
]- p53 MCF7
Scr si NONO C H2O2 Pif-α NAC
← β-Actin
TP-53
28/2/21

MCF-7

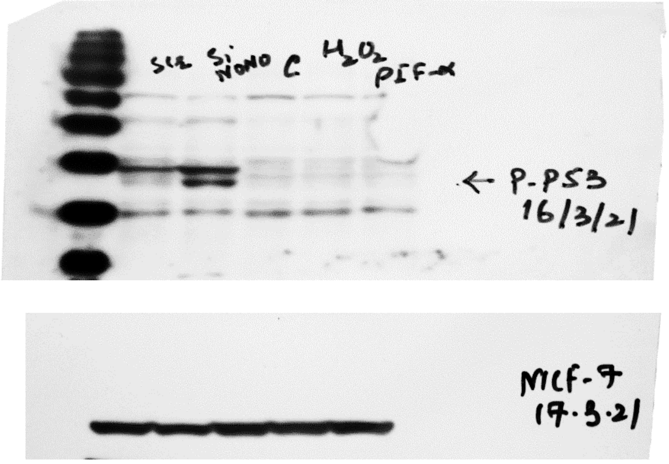

Fig. 7D

siRNA scr | siRNA NONO

MDA-MB-231

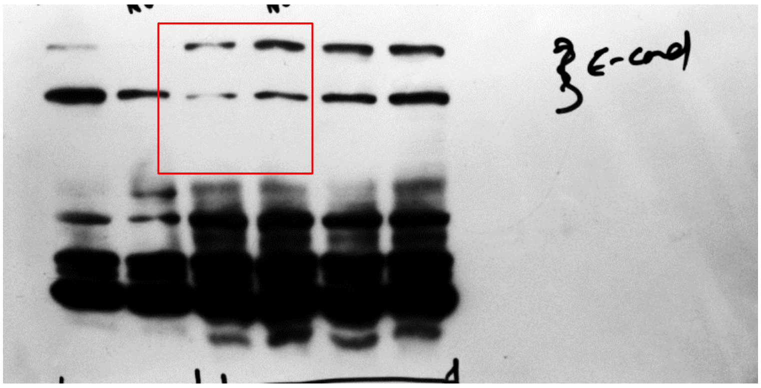

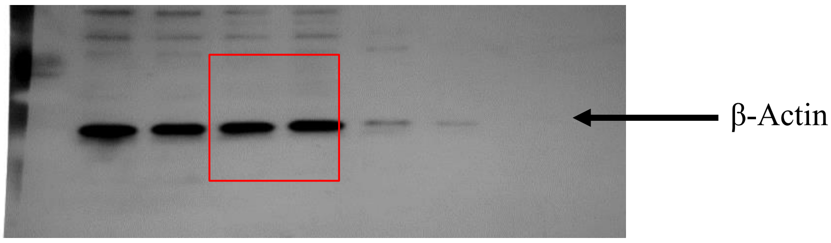

β-Actin

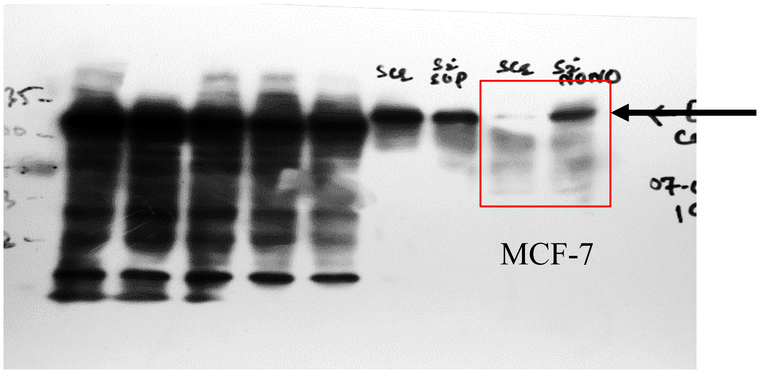

E-cadherin

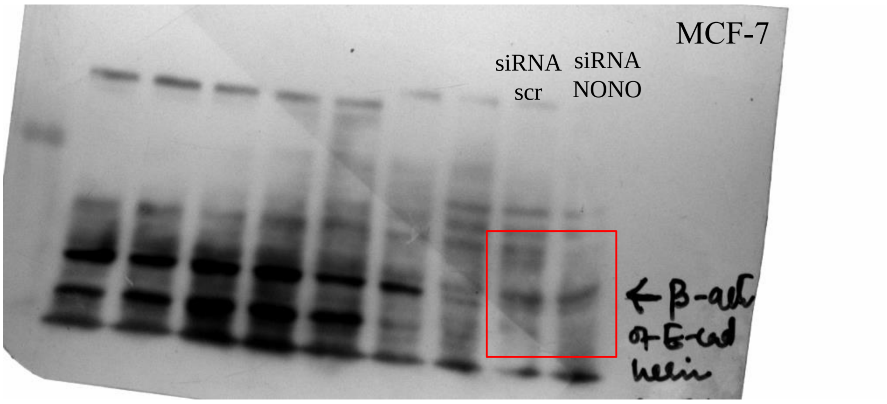
MCF-7
siRNA scr
siRNA NONO
β-act
of E-cad

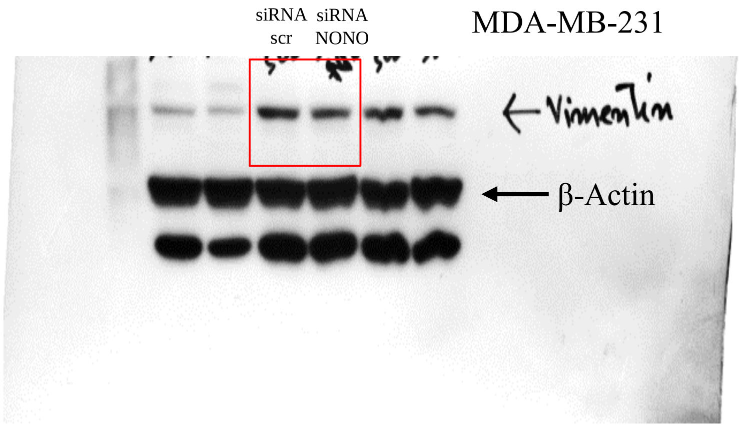
MDA-MB-231
siRNA scr
siRNA NONO
Vimentin
β-Actin

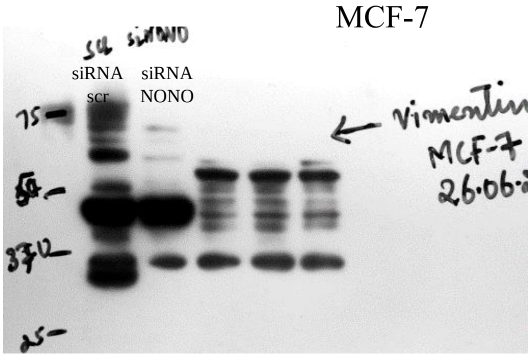
MCF-7
siRNA scr
siRNA NONO
75
50
37
25
Vimentin
MCF-7

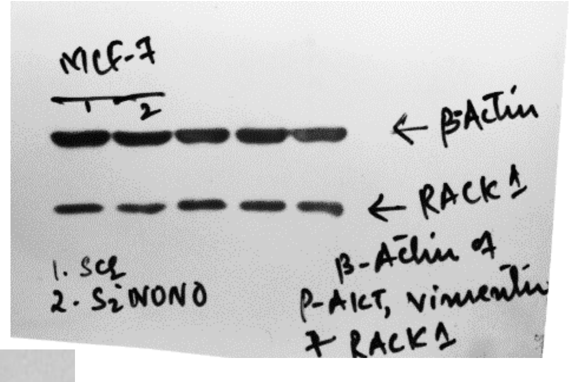
MCF-7
1
2
βActin
RACK1
1. Scr
2. SiNONO
β-Actin of
P-AKT, vimentin
+ RACK1

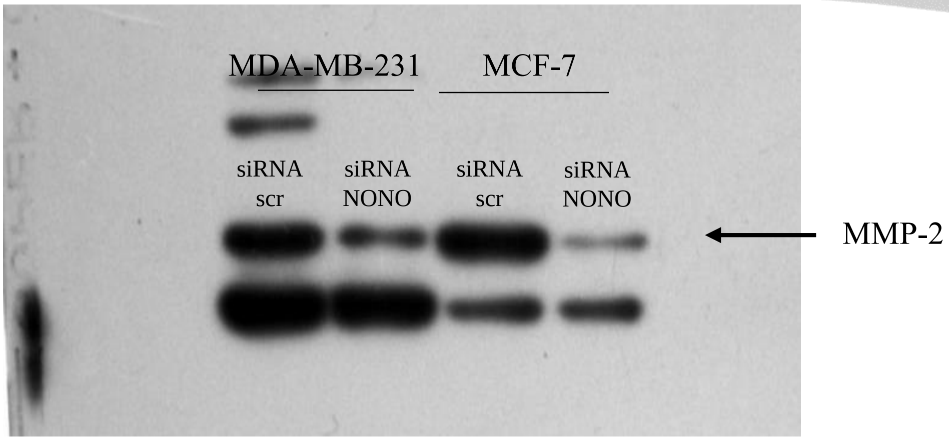
MDA-MB-231
MCF-7
siRNA scr
siRNA NONO
siRNA scr
siRNA NONO
MMP-2

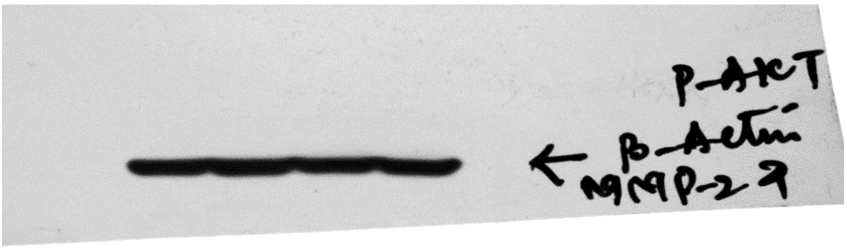
P-AKT
β-Actin
MMP-2

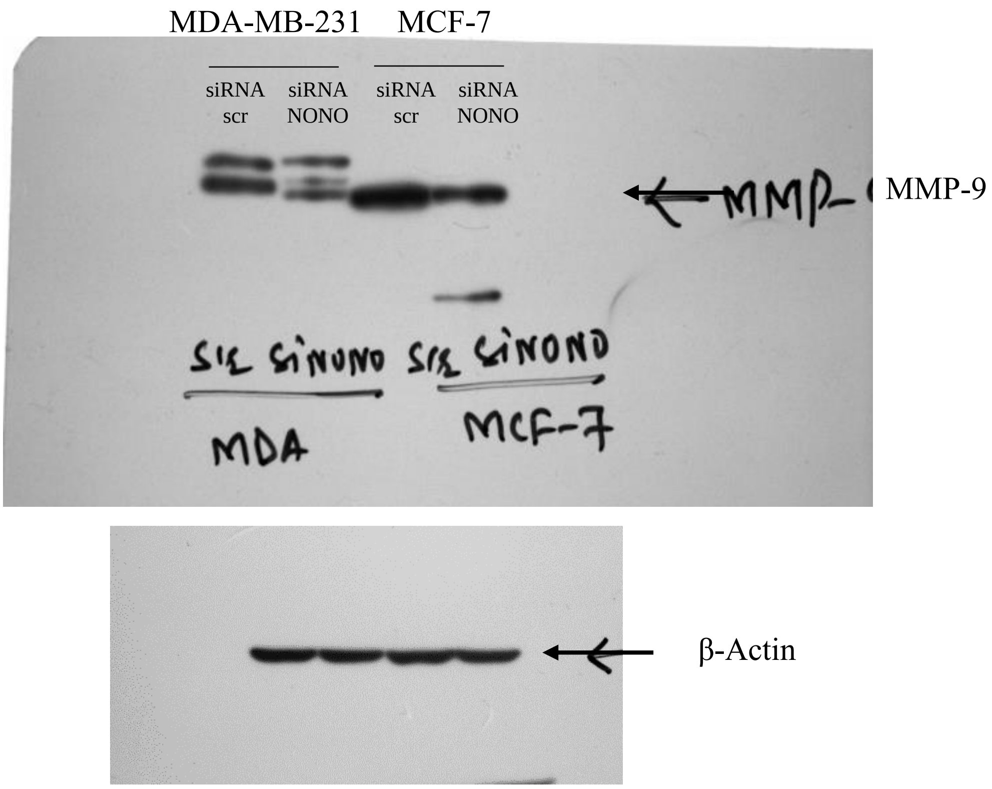
MDA-MB-231
MCF-7
siRNA scr
siRNA NONO
siRNA scr
siRNA NONO
MMP-9
MDA
MCF-7
β-Actin

Fig. 8F

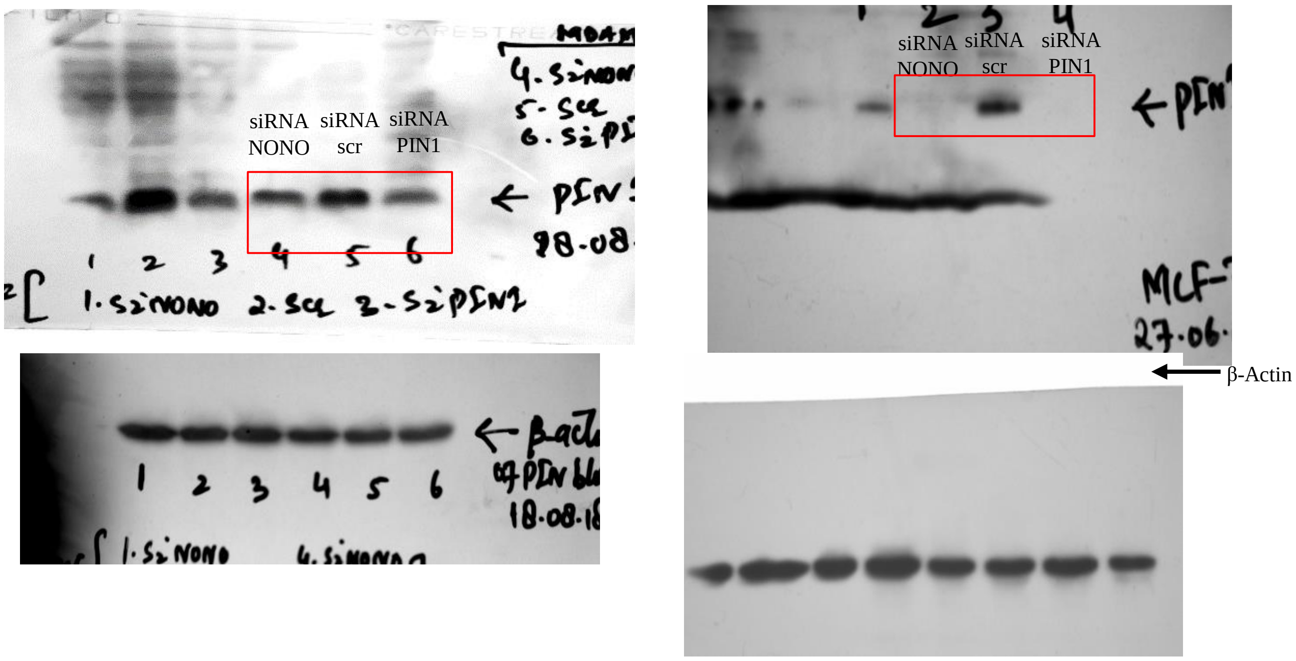
siRNA NONO
siRNA scr
siRNA PIN1
1 2 3 4 5 6
1 2 3 4 5 6
siRNA NONO
siRNA scr
siRNA PIN1
β-Actin

Fig. 10A

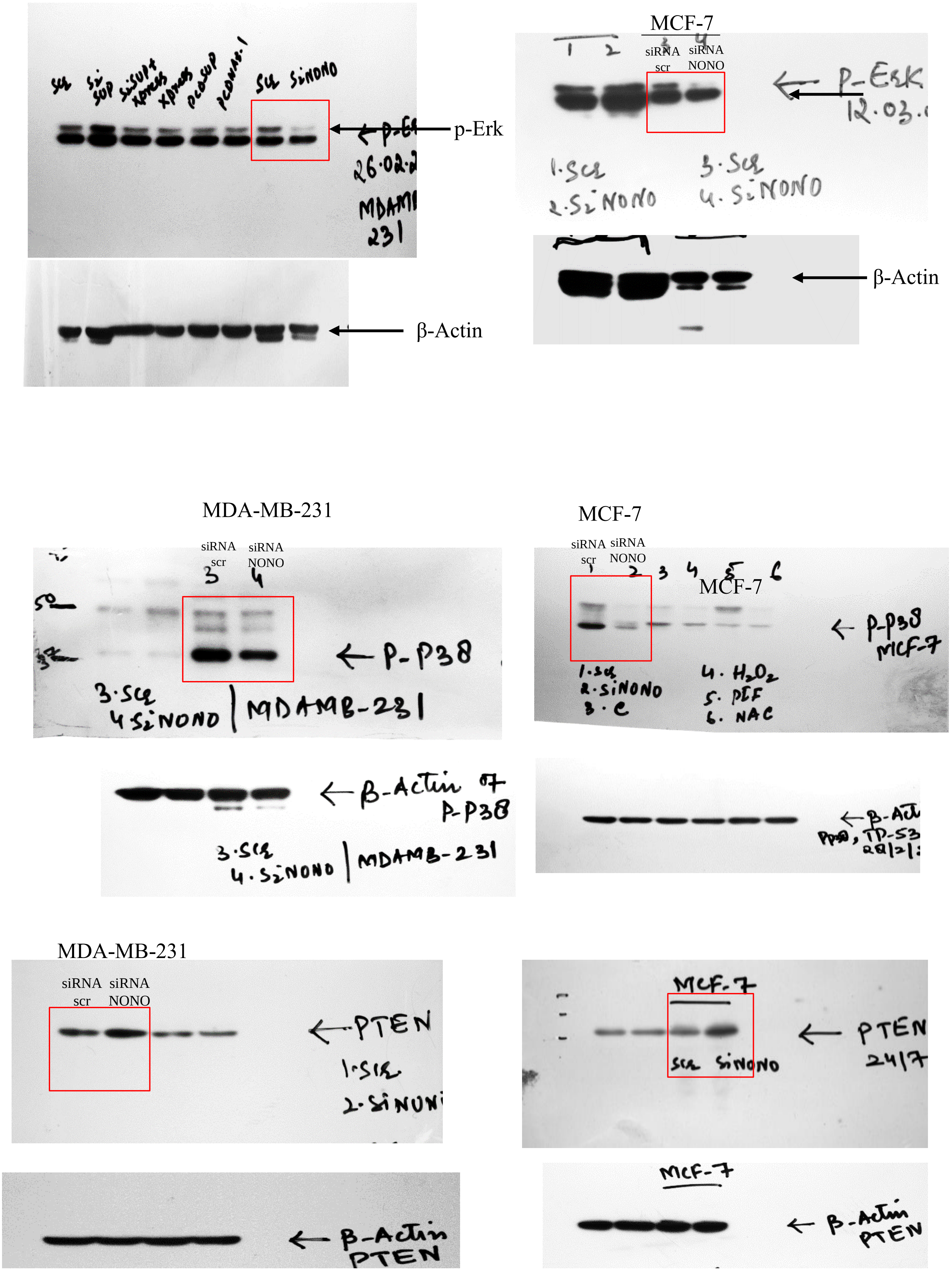
p-Erk
β-Actin
MCF-7
siRNA scr
siRNA NONO
β-Actin
MDA-MB-231
siRNA scr
siRNA NONO
MCF-7
siRNA scr
siRNA NONO
MCF-7
MDA-MB-231
siRNA scr
siRNA NONO

MDA-MB-231

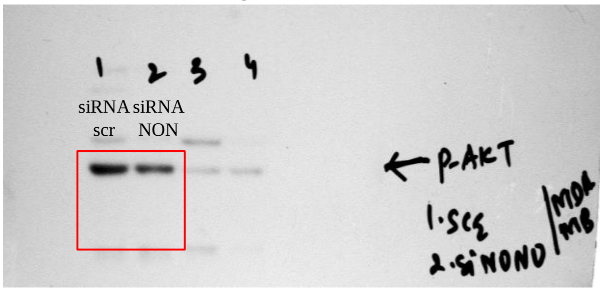

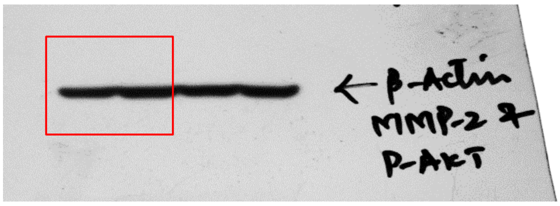

MCF-7

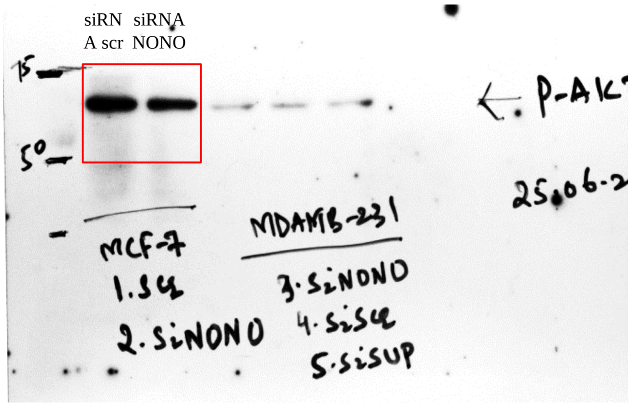

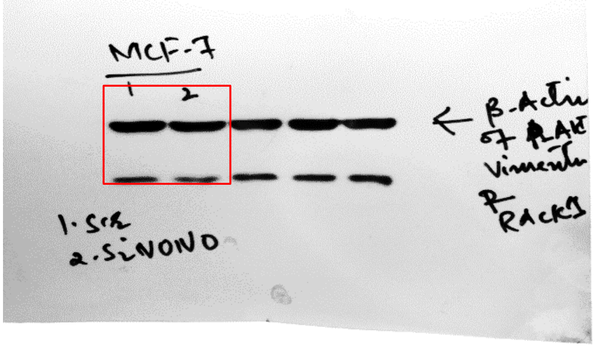

MDA-MB-231

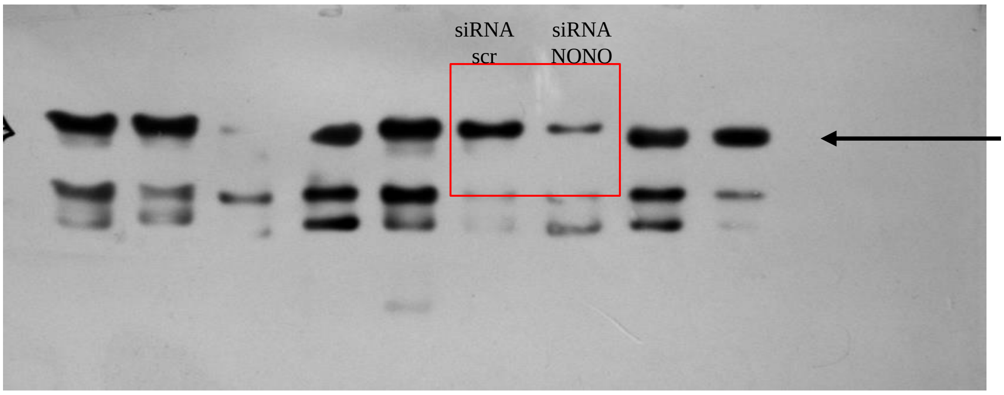

NF-kB

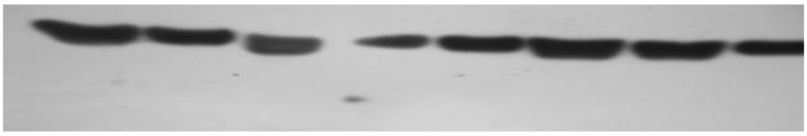

β-Actin

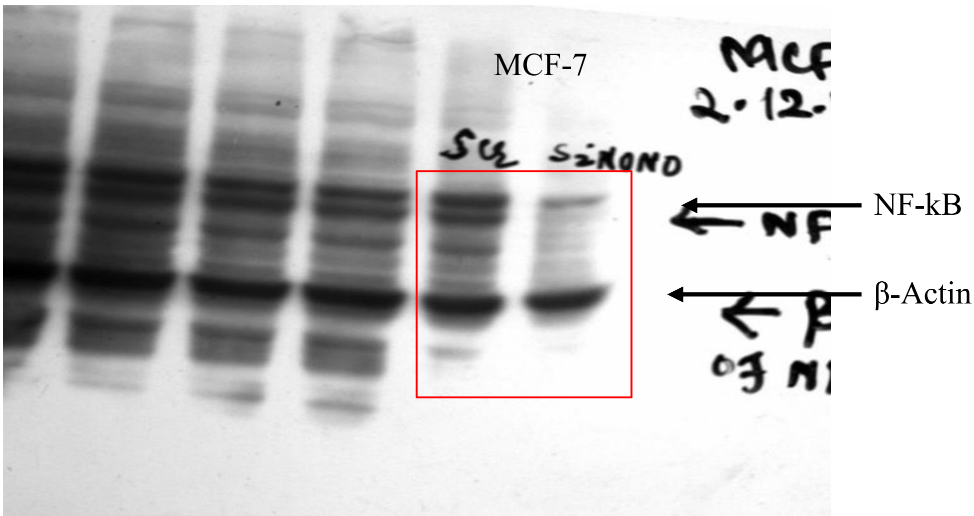

NF-kB

β-Actin

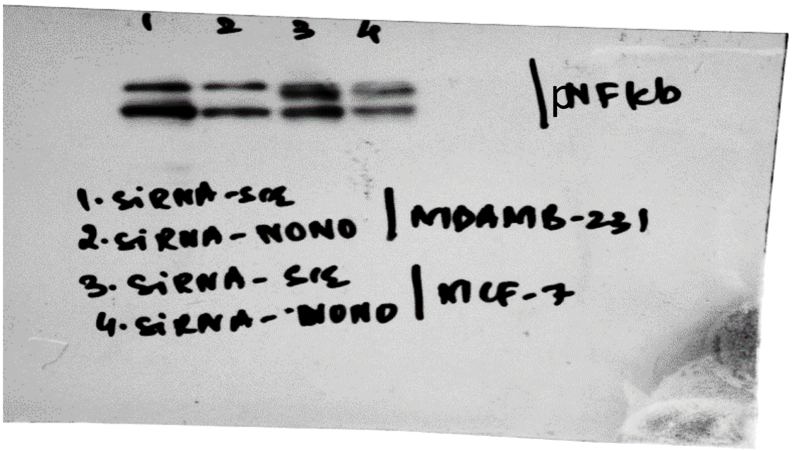

MDA-MB-231 MCF-7

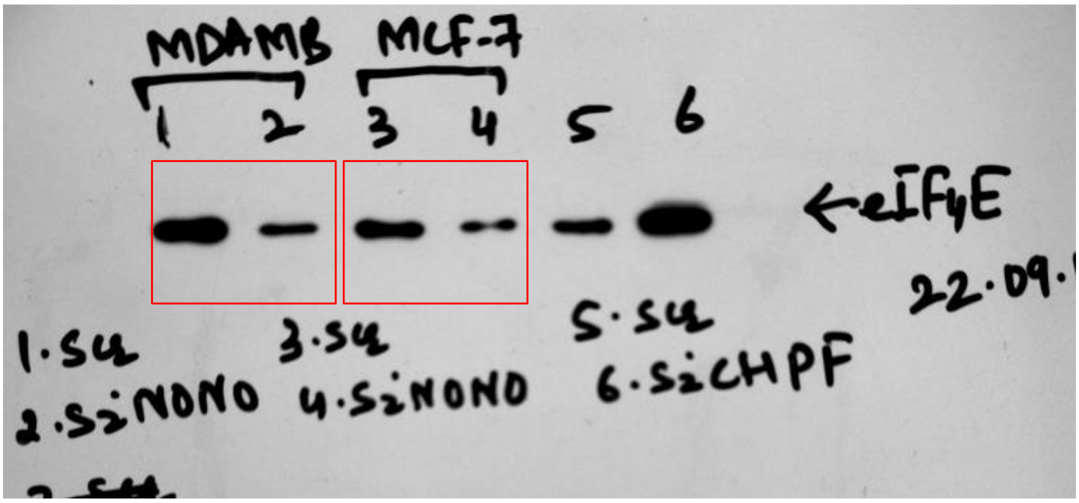

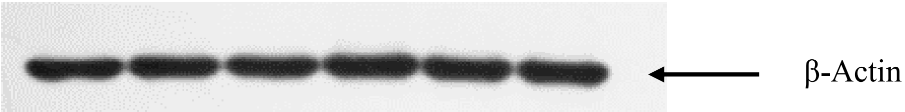

β-Actin

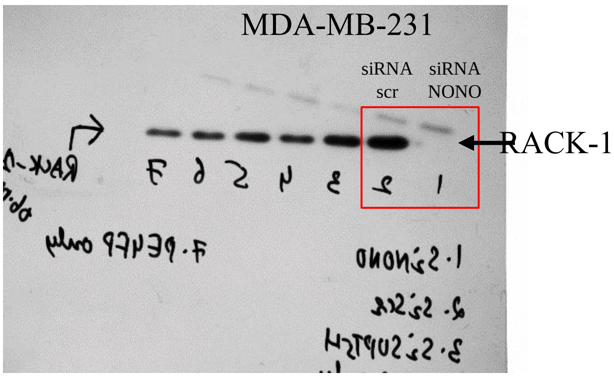

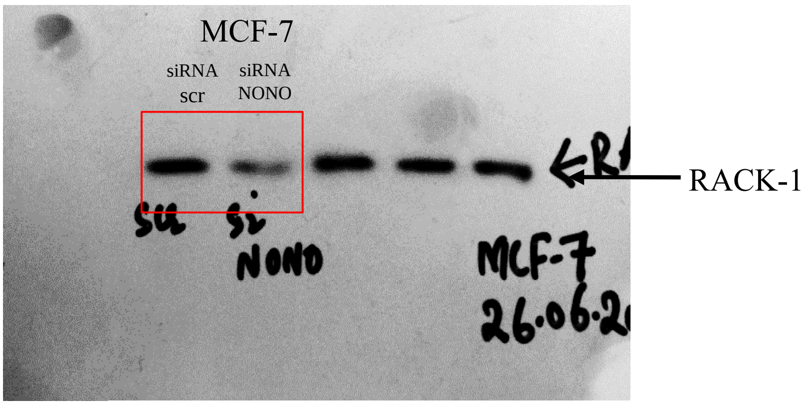

RACK-1

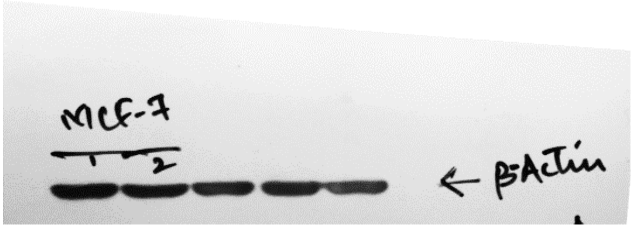

Fig. 10C

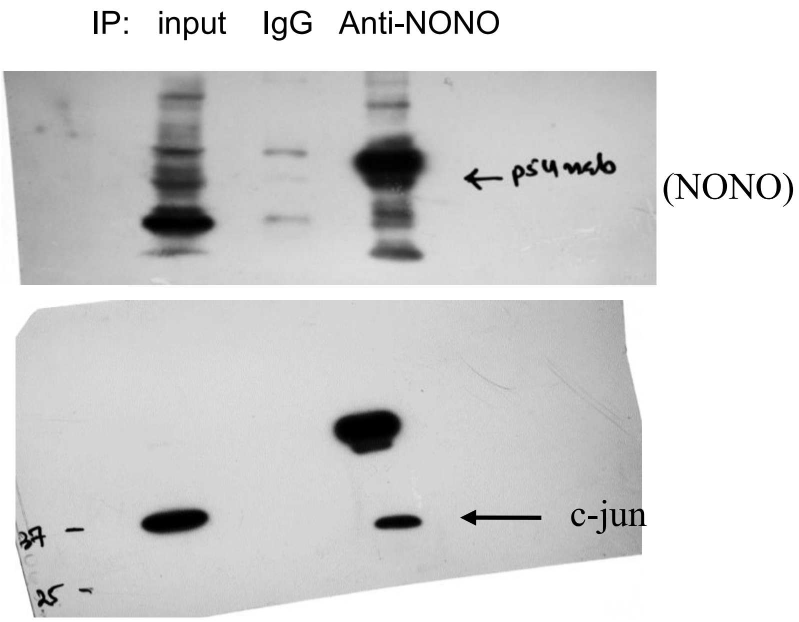

Fig. 10D

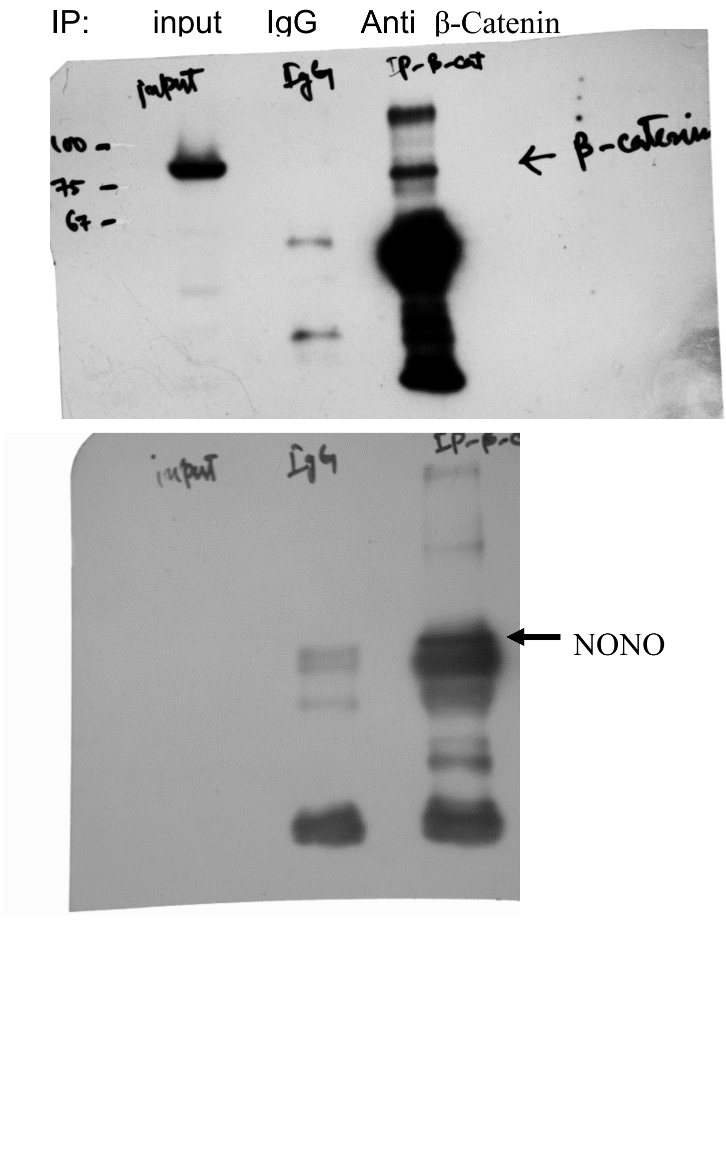

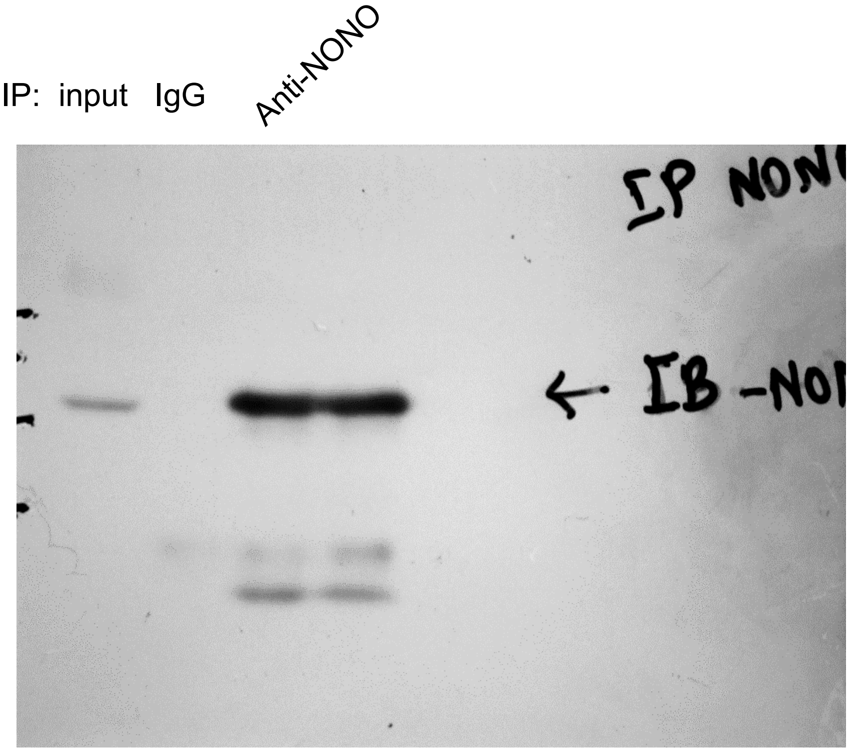
IP: input
IgG
Anti-NONO
IP NONO
IB -NO

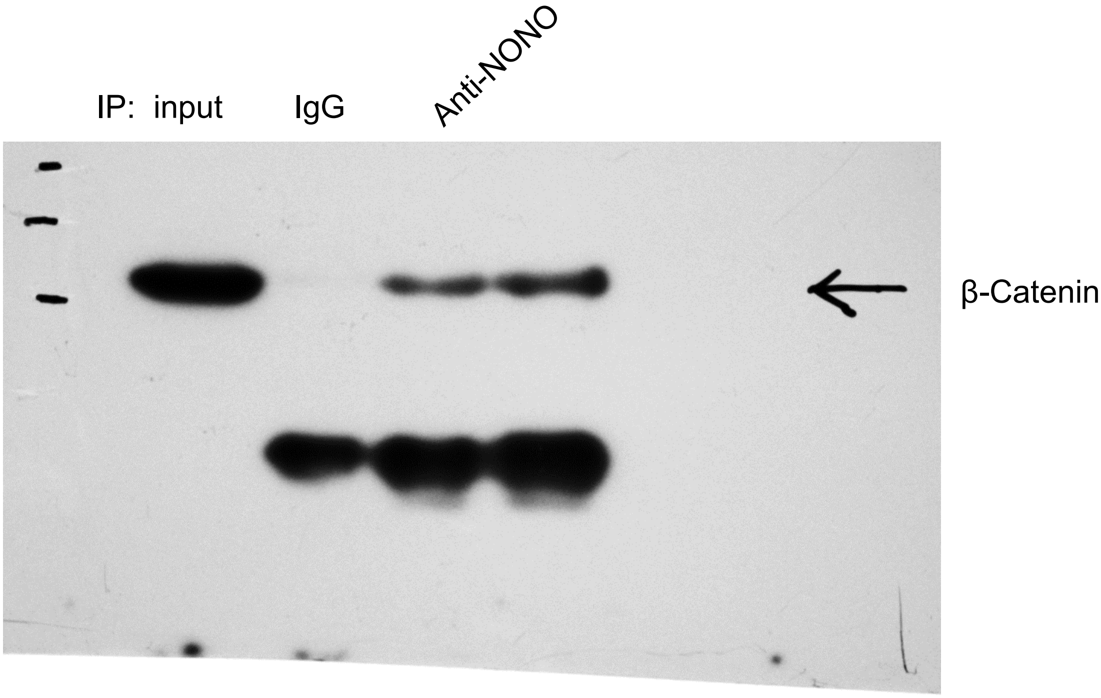
IP: input
IgG
Anti-NONO
β-Catenin

MDA-MB-231

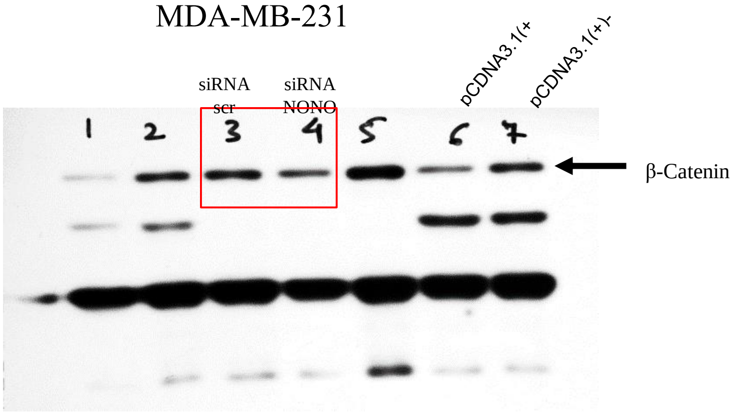

MCF-7

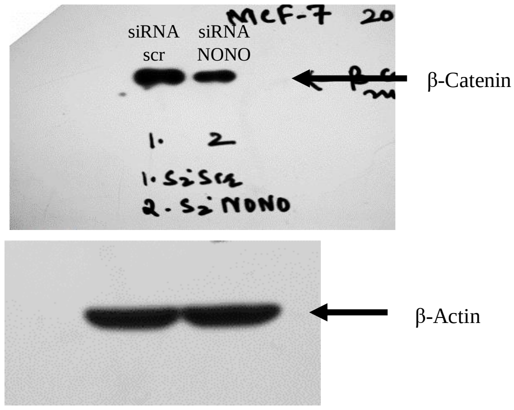

MDA-MB-231

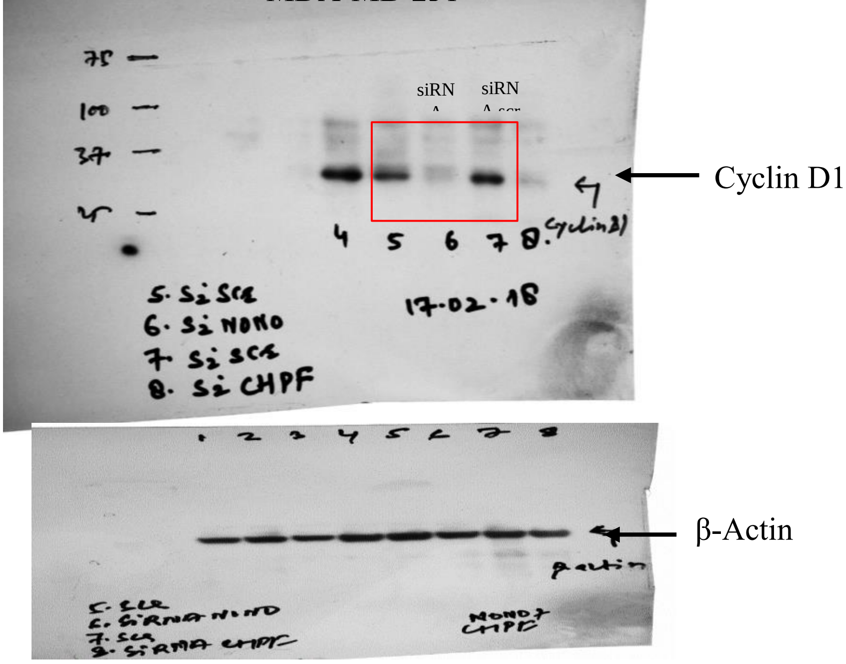

MCF-7

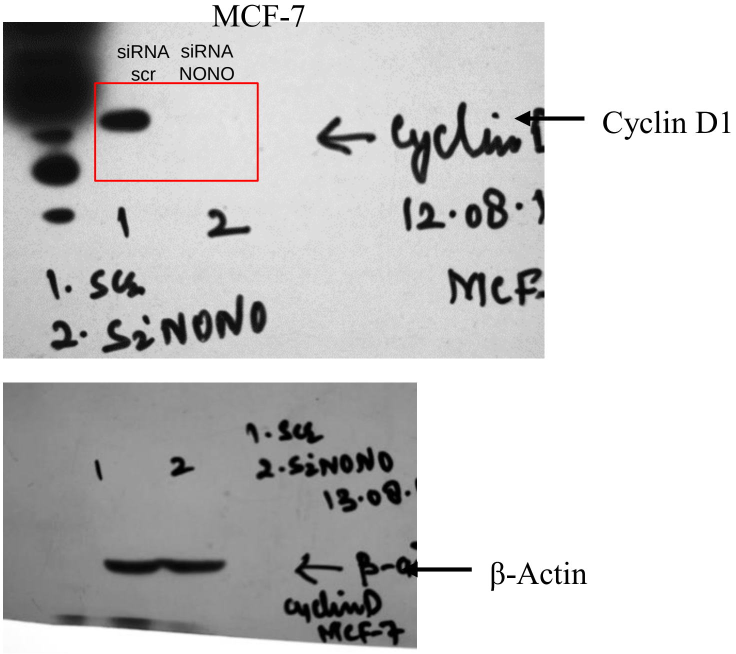

MDA-MB-231

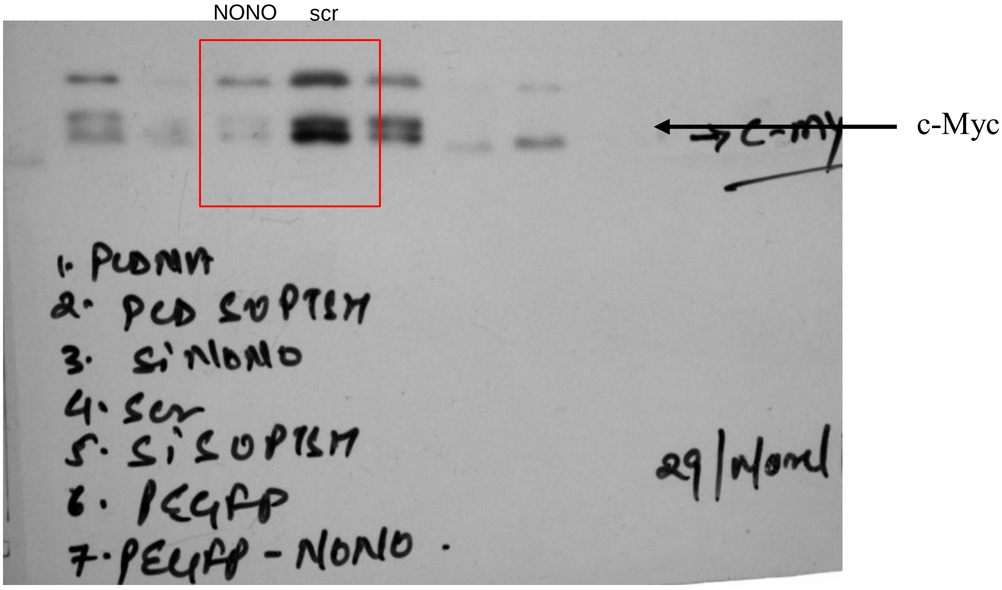

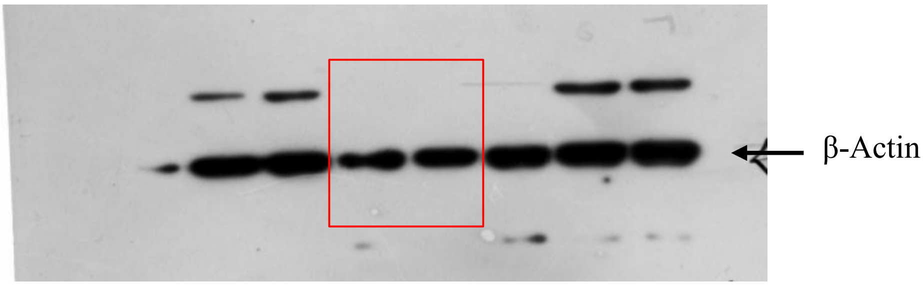

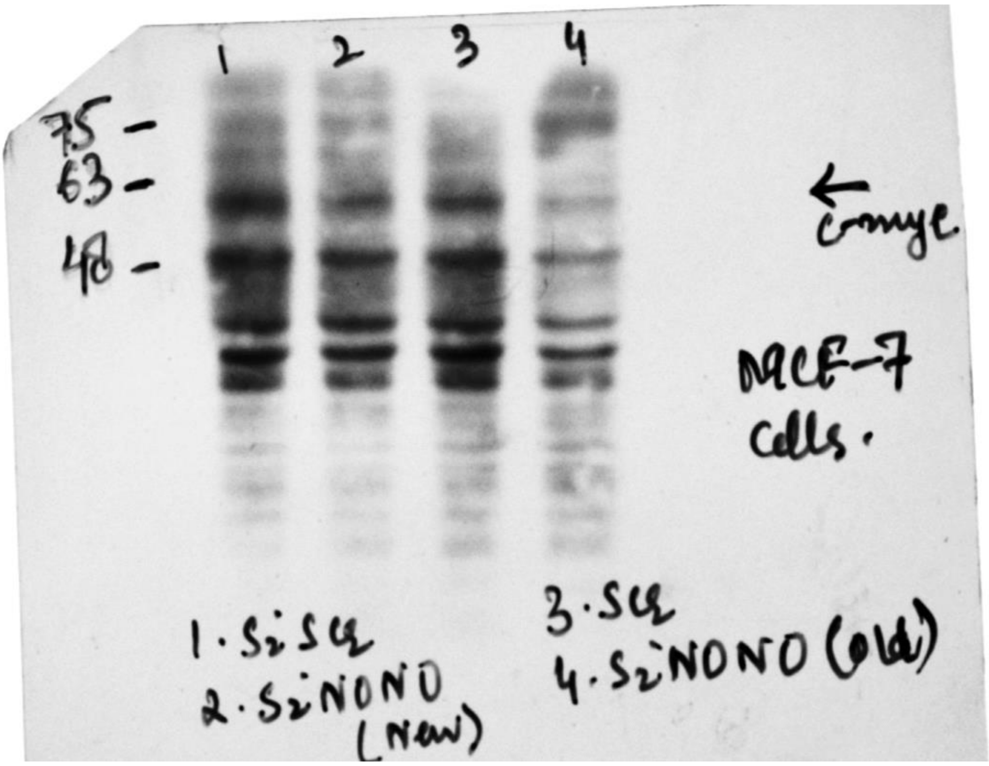

Fig. S1A

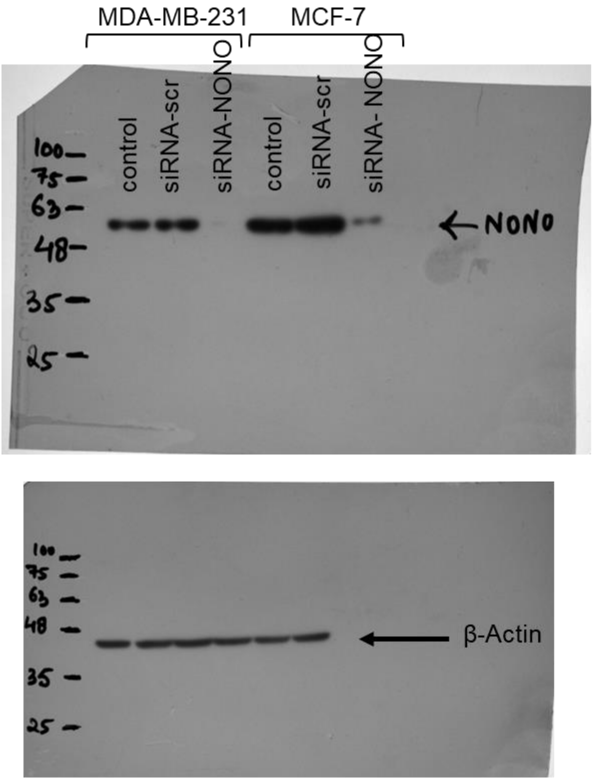

Fig. S1B

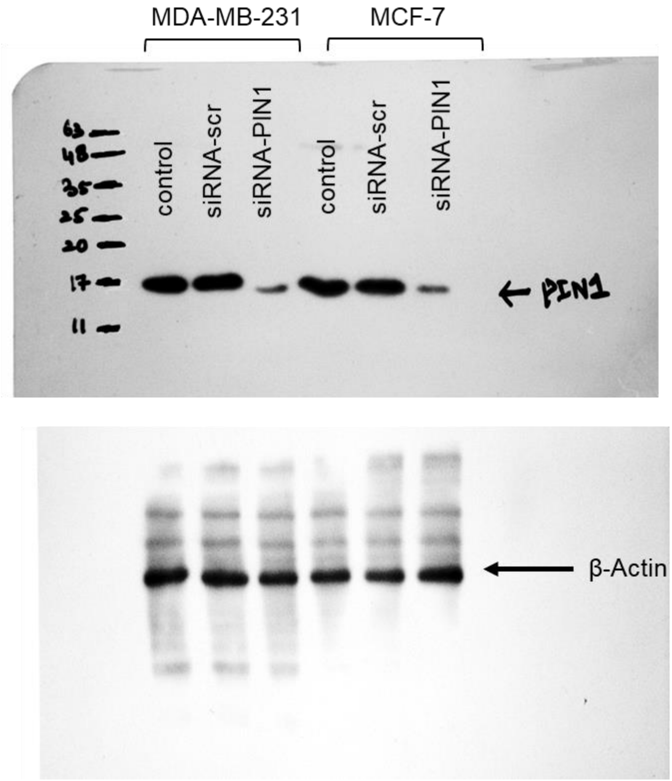

Fig. S4A

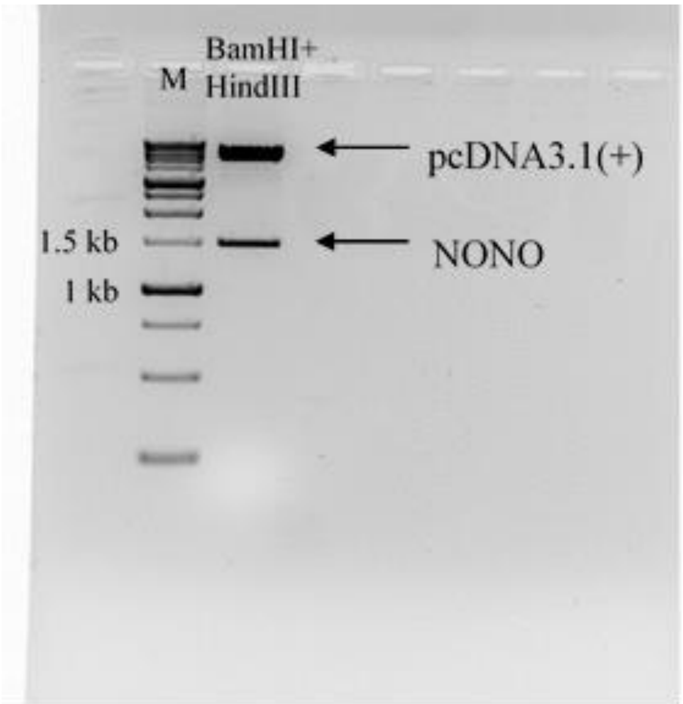

Fig. S4C

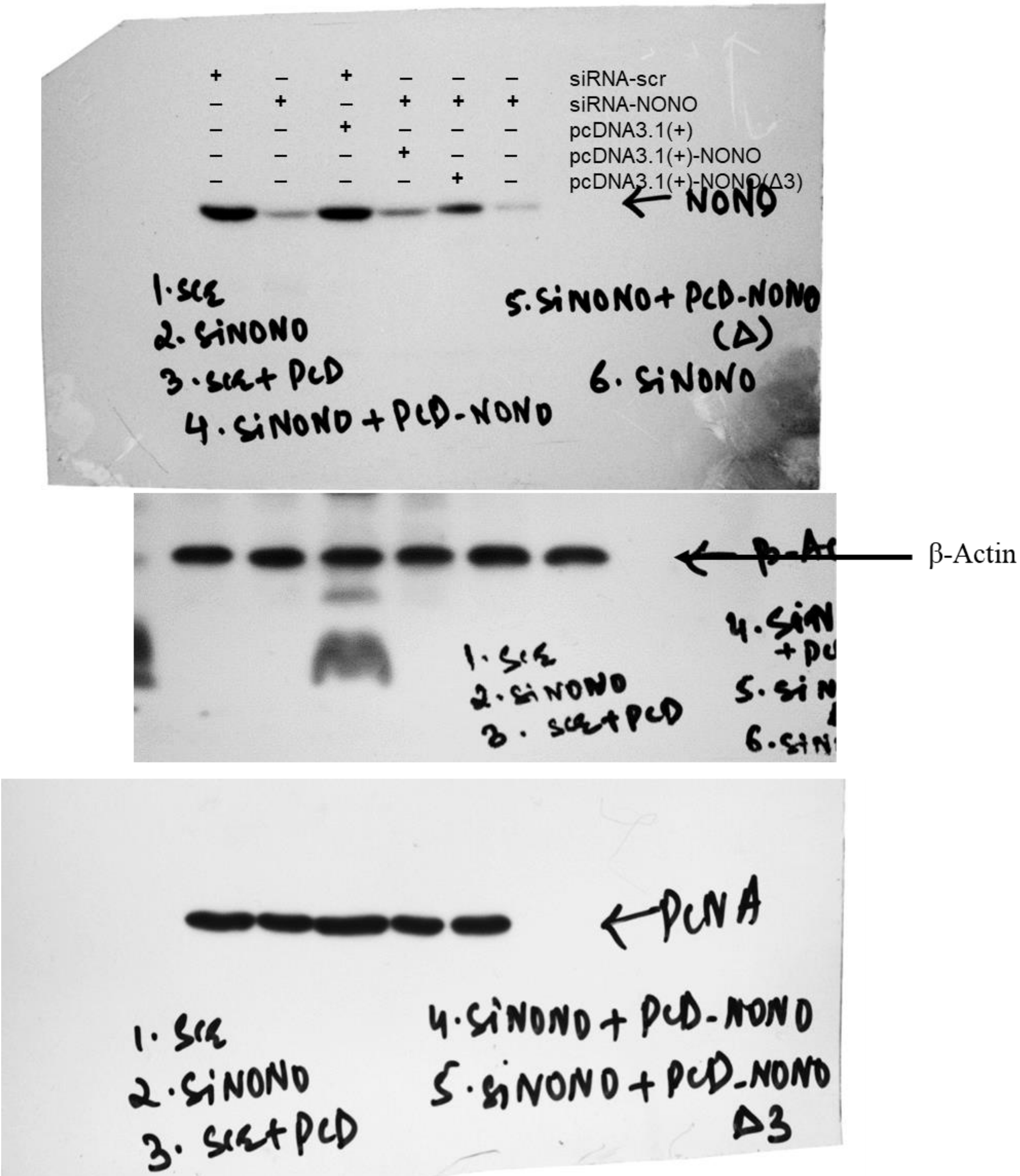

The same five out of six above mentioned samples were used to detect PCNA by western blotting, however after repeated attempts the β-actin was not detected in PCNA blot.

Fig. S5

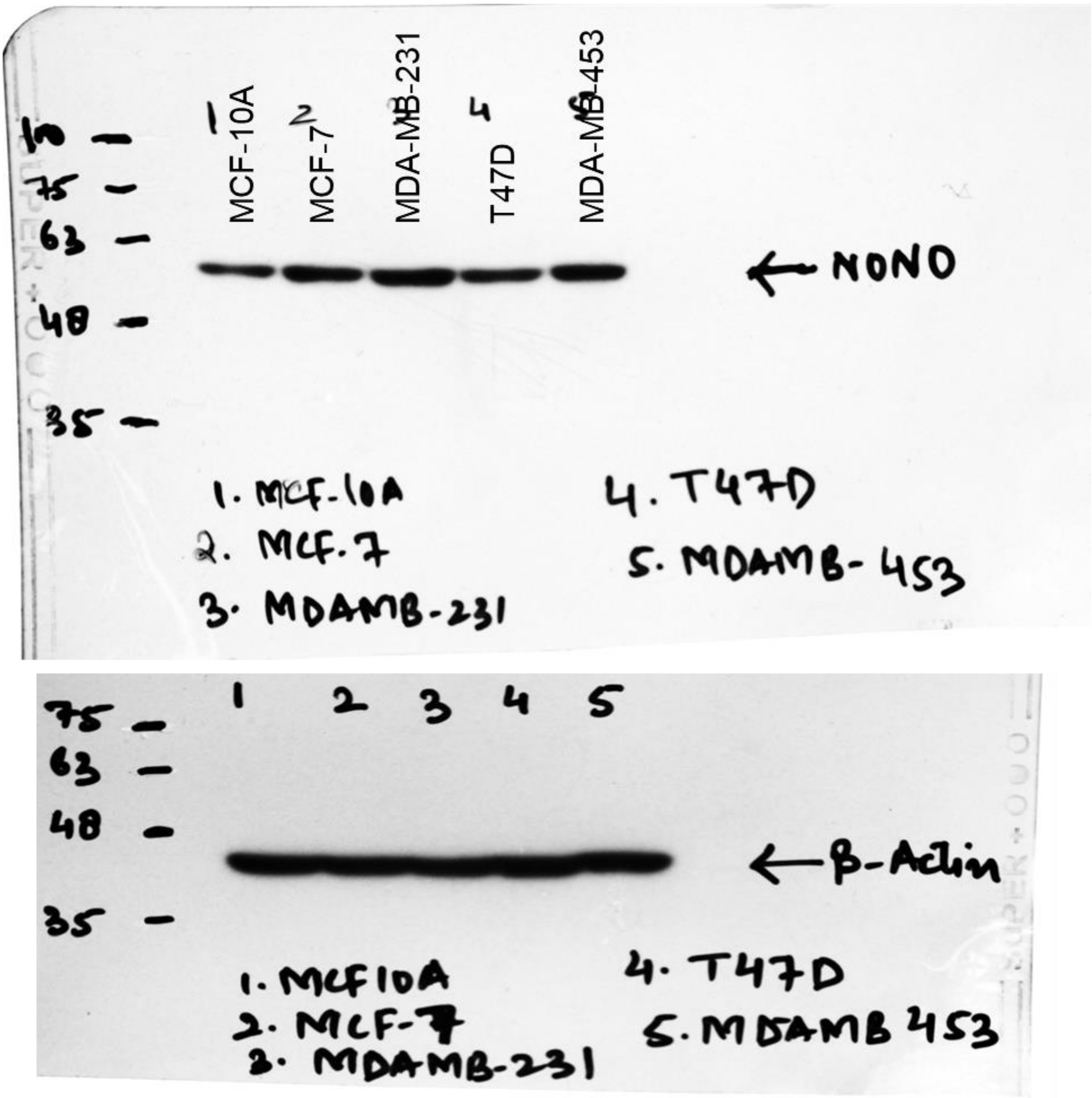

MCF-10A
MCF-7
MDA-MB-231
T47D
MDA-MB-453
1
2
4
10
75
63
48
35
← NONO
1. MCF-10A
2. MCF.7
3. MDAMB-231
4. T47D
5. MDAMB-453
1 2 3 4 5
75
63
48
35
← β-Actin
1. MCF10A
2. MCF-7
3. MDAMB-231
4. T47D
5. MDAMB 453

Fig. S8

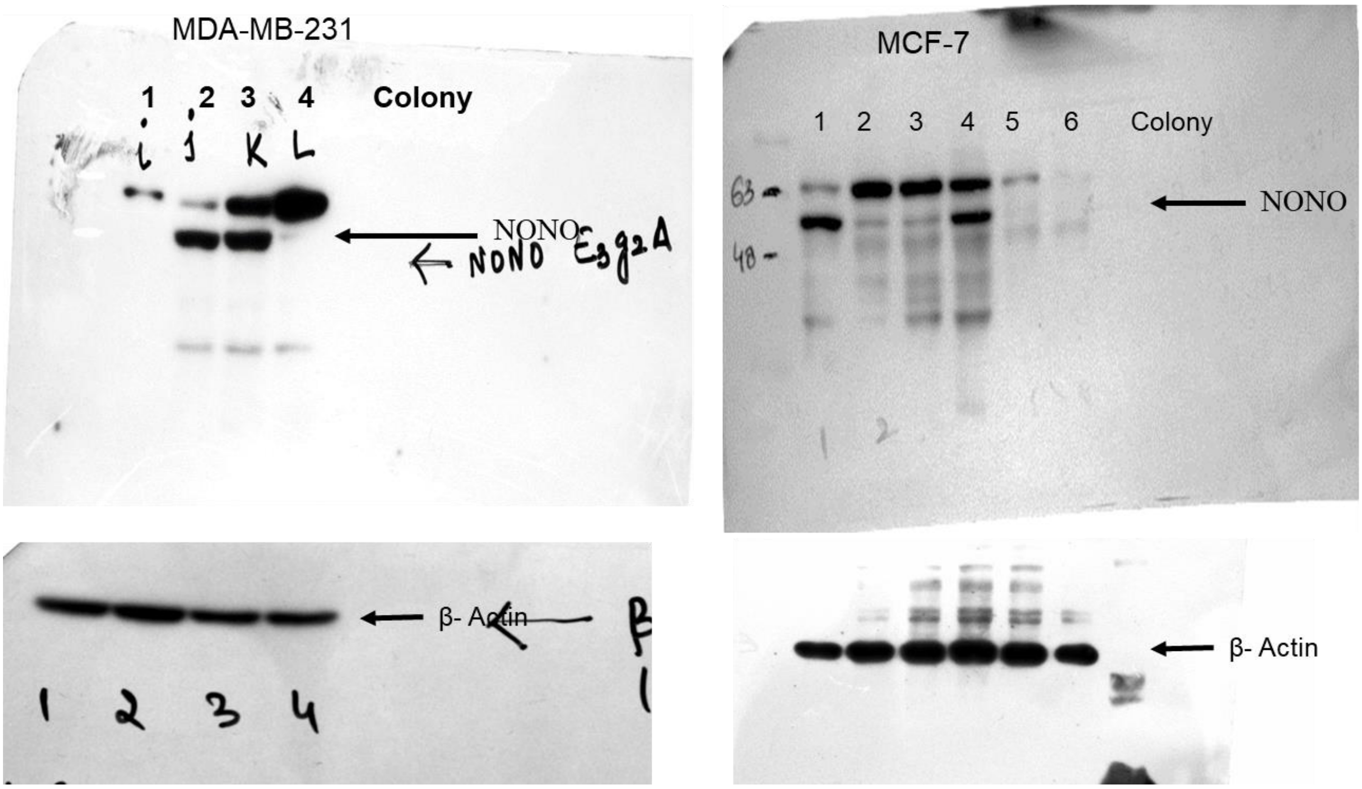
MDA-MB-231
1 2 3 4 Colony
NONO
β- Actin
1 2 3 4
MCF-7
1 2 3 4 5 6 Colony
63
48
NONO
β- Actin
